# Genes encoding norcoclaurine synthase occur as tandem fusions in the Papaveraceae

**DOI:** 10.1038/srep39256

**Published:** 2016-12-19

**Authors:** Jing Li, Eun-Jeong Lee, Limei Chang, Peter J. Facchini

**Affiliations:** 1 Department of Biological Sciences, University of Calgary, Calgary, Alberta T2N 1N4, Canada

## Abstract

Norcoclaurine synthase (NCS) catalyzes the enantioselective Pictet-Spengler condensation of dopamine and 4-hydroxyphenylacetaldehyde as the first step in benzylisoquinoline alkaloid (BIA) biosynthesis. NCS orthologs in available transcriptome databases were screened for variants that might improve the low yield of BIAs in engineered microorganisms. Databases for 21 BIA-producing species from four plant families yielded 33 assembled contigs with homology to characterized *NCS* genes. Predicted translation products generated from nine contigs consisted of two to five sequential repeats, each containing most of the sequence found in single-domain enzymes. Assembled contigs containing tandem domain repeats were detected only in members of the Papaveraceae family, including opium poppy (*Papaver somniferum*). Fourteen cDNAs were generated from 10 species, five of which encoded NCS orthologs with repeated domains. Functional analysis of corresponding recombinant proteins yielded six active NCS enzymes, including four containing either two, three or four repeated catalytic domains. Truncation of the first 25 N-terminal amino acids from the remaining polypeptides revealed two additional enzymes. Multiple catalytic domains correlated with a proportional increase in catalytic efficiency. Expression of *NCS* genes in *Saccharomyces cereviseae* also produced active enzymes. The metabolic conversion capacity of engineered yeast positively correlated with the number of repeated domains.

Norcoclaurine synthase (NCS; EC 4.2.1.78) catalyzes the enantioselective Pictet-Spengler condensation of the L-Tyr derivatives dopamine and 4-hydroxyphenylacetaldehyde (4-HPAA) yielding (*S*)-norcoclaurine, which serves as the central intermediate in benzylisoquinoline alkaloid (BIA) biosynthesis in plants^1^,^2^. BIAs are a large and structurally diverse group of natural products found primarily in four related plant families: Papaveraceae, Ranunculaceae, Berberidaceae and Menispermaceae. In contrast to the enzymatic condensation, uncatalyzed Pictet-Spengler reactions between various phenylethylamines and aldehydes yield racemic reaction products^3^. (*S*)-Norcoclaurine is subsequently converted via sequential 6-*O*-methylation, *N*-methylation, 3′-hydroxylation, and 4′-*O*-methylation to the branch point intermediate (*S*)-reticuline, which undergoes internal oxidative carbon-carbon coupling to generate an array of scaffold structures^4^. Metabolic end products derived from (*S*)-norcoclaurine possess a variety of pharmacological activities including the antimicrobial agents berberine and sanguinarine, the narcotic analgesic morphine, the antitussive and microtubule disruptor noscapine, and the vasodilator papaverine (Fig. 1).

A *NCS* gene was previously isolated based on empirical amino acid sequences of peptides obtained by tryptic digestion of the purified NCS enzyme from meadow rue (*Thalictrum flavum*; Ranunculaceae), which primarily accumulates protoberberine alkaloids^5^,^6^,^7^. *T. flavum* NCS (TFLNCS) was subsequently used to query opium poppy (*Papaver somniferum*) EST databases, resulting in the isolation of two isoforms (PSONCS1 and PSONCS2) both catalyzing the formation of (*S*)-norcoclaurine from dopamine and 4-HPAA, and displaying 89% conserved amino acid sequence identity, but only 40% identity compared with TFLNCS^8^. NCS variants from *T. flavum* and *P. somniferum* also showed 30–40% identity with members of the family of pathogenesis-related 10 (PR10) protein/Bet v1 allergens^9^. Investigations into the reaction mechanism leading to the formation of (*S*)-norcoclaurine from dopamine and 4-HPAA suggested the two-step cyclization of a putative iminium ion intermediate^10^,^11^. The general structure of TFLNCS was initially obtained by NMR spectroscopy coupled with homology modeling of Bet v1 proteins^12^, and the specific structural determinants responsible for stereoselective Pictet-Spengler cyclization were established by X-ray crystallographic analysis^13^,^14^. Interestingly, NCS is the only known PR10/Bet v1 protein shown unequivocally to exhibit a catalytic function.

The stereoselective activity of NCS has prompted applications of the enzyme in both *in vitro* biocatalysis^15^,^16^,^17^ and *in vivo* metabolic engineering for the purpose of synthesizing high-value BIAs^18^,^19^,^20^,^21^,^22^,^23^,^24^. However, measured kinetic parameters for NCS suggest an enzyme that is catalytically inefficient^6^,^7^,^14^,^25^,^26^. NCS has been reported to display an apparent catalytic efficiency (*k*_cat_/*K*_m_) of 1.0 mM^−1^ s^−1^, which is 100-fold lower than the median of 125 mM^−1^ s^−1^ calculated across all enzymes^27^, and a high apparent *K*_m_ for dopamine interpreted as an indication that high cellular substrate concentrations are required for significant turnover^26^. The purported catalytic inefficiency of NCS could explain the relatively low turnover capacity of the enzyme in engineered microorganisms.

The improvement of enzyme performance in metabolically engineered systems can be achieved through a variety of means^27^, including the identification and deployment of functionally conserved variants from related organisms^28^. In this paper, we report the isolation and characterization of NCS variants from several plant species related to *T. flavum* and *P. somniferum*, and we demonstrate their general functionality in yeast (*Saccharomyces cerevisiae*). Unexpectedly, orthologs encoding NCS variants in the Papaveraceae were found to occur naturally as tandem fusions consisting of two or more complete and active catalytic domains. Some paralogs of single- or multiple-domain proteins contained incomplete catalytic domains, or other features, and were not enzymatically active. The enzymological and metabolic implications of NCS variants from various plant species are investigated.

## Results

### Isolation and phylogeny of NCS candidates

**Figure 2:**
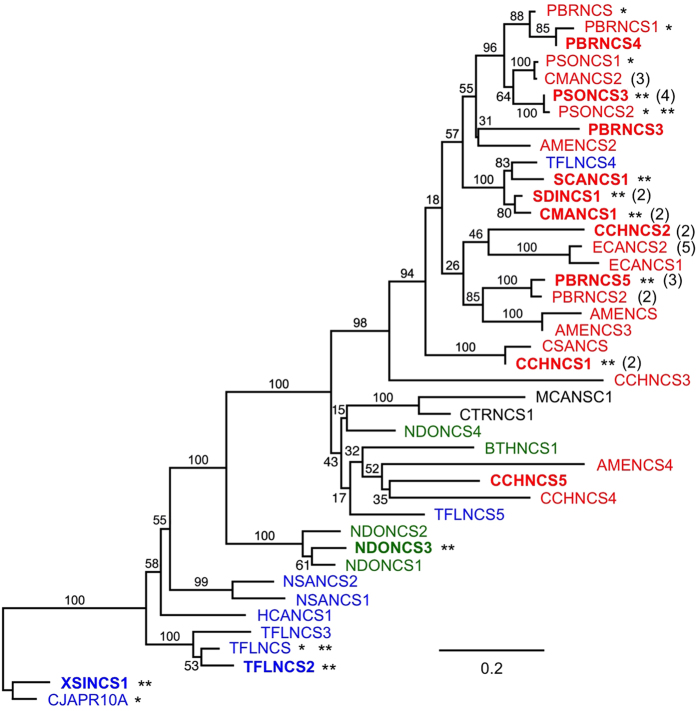
Predicted phylogenetic relationships among enzymatically characterized and putative NCS variants in 20 plant species from four families: Papaveraceae (red), Berberidaceae (green), Menispermaceae (black) and Ranunculaceae (blue). Fourteen recombinant enzymes tested for NCS activity are shown in bold. Single asterisks show previously characterized active NCS enzymes. Double asterisks indicate active NCS enzymes in *Escherichia coli* and *Saccharomyces cerevisiae*. Values in parentheses indicate the number of sequentially repetitive domains in enzyme fusions. Scale bar represents amino acid substitutions per site.

**Figure 3:**
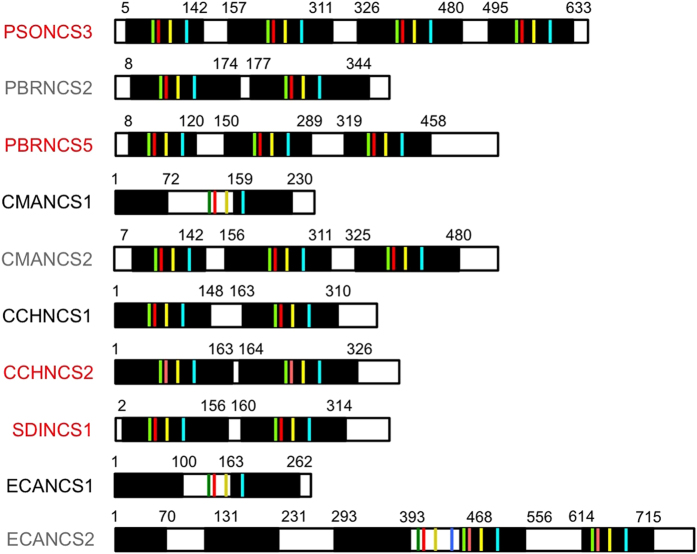
Organization of sequentially repeated domains and key catalytic residues in fused NCS enzymes. Black bars represent the location and relative length of duplicated regions in each protein. Green, red, yellow and blue lines represent four identified catalytic resides: Y, E, K and D, respectively^14^. Active NCS enzymes are named in red, whereas tested, but functionally inactive NCS homologs are named in black. NCS homologs named in grey were not successfully isolated by RT-PCR and, thus, were not tested.

BLASTx analysis of PhytoMetaSyn Project databases revealed 33 candidate genes encoding polypeptides displaying >30% amino acid identity with NCS from either *P. somniferum* (PSONCS) or *T. flavum* (TFLNCS) (Fig. 2). Phylogenetic analysis showed a strong relationship among NCS candidates from the same family. Other previously characterized NCS enzymes from *Papaver bracteatum*^29^ and *Coptis japonica*^30^ shared considerable identity with those from *P. somniferum* (PSONCS) and *T.flavum* (TFLNCS), respectively. Amino acid sequences from *P. bracteatum* and *C. japonica* represented the most distant branches on the phylogenetic tree, with the 33 NCS candidates showing intermediate levels of amino acid sequence identity between these two extremes. Ten of 33 candidates, all within the Papaveraceae family, were represented in the assembled Illumina and 454 sequence databases as genes encoding fusion proteins containing two to five tandem repeats of complete catalytic domains, or partial domains with lengths between 70 and 170 amino acids (Fig. 3). The crystallographic structure for TFLNCS showed that Y^108^, E^110^, K^122^ and D^141^ dominate catalysis in the active site^14^. Amino acid sequence alignments (Supplemental Fig. 1) showed that the repeated domains in most fusion proteins each contained a full set of these four proposed catalytic residues. However, two fusion proteins (CMANCS1 and ECANCS1), both containing two partial domains, each possessed only one complete set of catalytic residues (Fig. 3). Similarly, the predicted ECANCS2 polypeptide contained five tandem repeats, but displayed only three sets of NCS catalytic residues (Fig. 3). Moreover, catalytic residues in CMANCS1, ECANCS1 and ECANCS2 were not always located in repeated regions of conserved domains.

### Expression of NCS candidates in *Escherichia coli*

Fourteen genes encoding NCS candidates were amplified from corresponding cDNA libraries. Eight of the genes encoded proteins with single NCS domains (Fig. 2), whereas six genes encoded fusion proteins containing tandem repeats of two to four NCS domains (Figs 2 and 3). Amplification of the remaining three putative NCS fusion proteins (i.e. PBRNCS2, CMANCS2 and ECANCS2) was unsuccessful, potentially as the result of DNA sequence or assembly errors affecting annealing sites for the selected PCR primers (Table S1). Immunoblot analysis confirmed the production of soluble polypeptides in *E. coli* corresponding to 14 recombinant proteins, all of which showed empirical molecular weights in agreement with the theoretical size of predicted translation products (Supplemental Fig. 2A).

**Figure 4:**
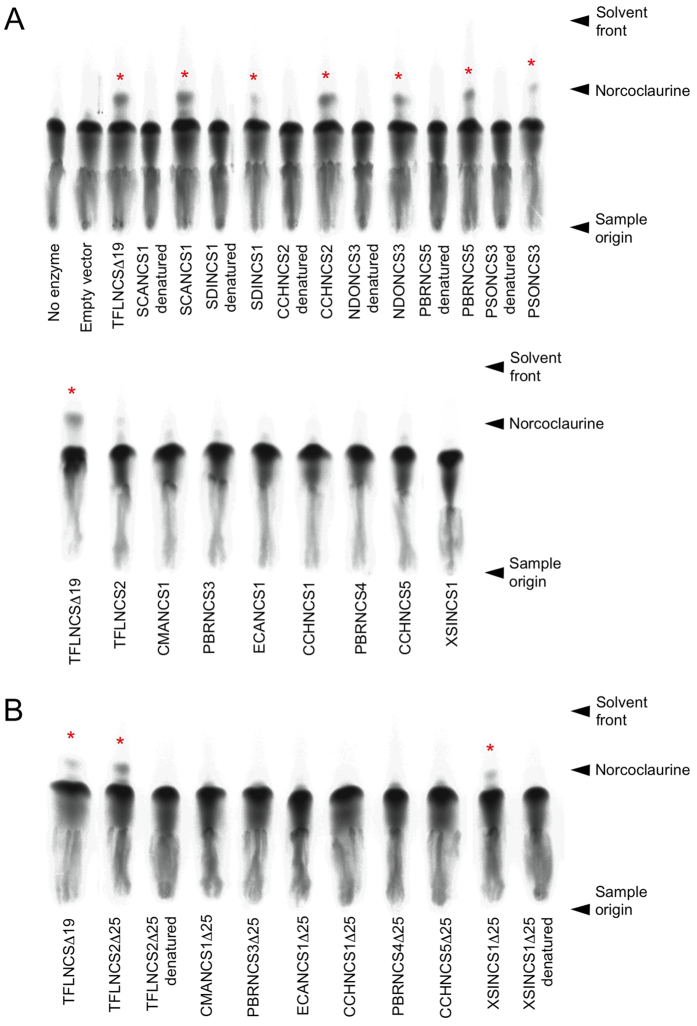
Functional characterization of 14 recombinant NCS enzymes from different plant species produced in *Escherichia coli*. (**A**) Six of the 14 soluble recombinant NCS candidates showed NCS activity *in vitro*. Crude soluble protein from *E. coli* producing each candidate was assayed for NCS activity using [8-^14^C]-dopamine and 4-HPAA. Affinity-purified, recombinant TFLNCSΔ19 and a total protein extract from *E. coli* harboring the empty pET29b vector were used as positive and negative controls for NCS activity. The [^14^C]-norcoclaurine reaction product (labeled with asterisks) was separated using thin layer chromatography and visualized by autoradiography. [^14^C]-Norcoclaurine was not detected in corresponding protein extracts denatured by boiling for 5 min. (**B**) Truncation of the first 25 amino acids from the N-terminus of each of the 8 inactive full-length NCS candidates resulted in the detection of NCS activity in two proteins. Affinity-purified, recombinant TFLNCSΔ19 was used as a positive control for NCS activity.

Using crude *E. coli* protein extracts, six of the fourteen soluble recombinant enzymes converted [^14^C]-dopamine and 4-HPAA to [^14^C]-norcoclaurine (Fig. 4A). One additional candidate, TFLNCS2, also showed a possible low level of activity. Negative control reactions containing only buffer and substrates, or crude soluble protein from *E. coli* harboring the empty pET29b vector did not yield detectable [^14^C]-norcoclaurine (Fig. 4A). Truncation of the first 25 amino acids from eight NCS candidates that did not efficiently convert [^14^C]-dopamine and 4-HPAA to [^14^C]-norcoclaurine as nascent proteins (i.e. retaining a putative N-terminal signal peptide) yielded two additional enzymes with substantial activity, TFLNCS2 and XSINCS1 (Fig. 4B). Remarkably, the amino acid sequence identity among several of the active enzymes was less than 35%, but was as high as 90% between variants from closely related species (Supplemental Fig. 1). In contrast, some catalytically inactive proteins showed amino acid sequence identities of 94–96% compared with active enzymes.

**Figure 5:**
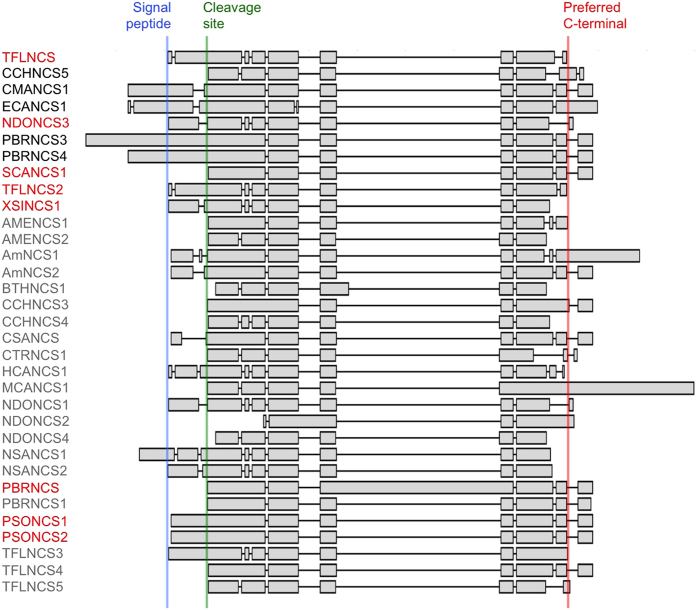
Schematic representation of the amino acid sequence alignment for singlet NCS polypeptides compared with TFLNCS. Polypeptides from position 2 to 10 were functionally characterized. PSONCS1 and PSONCS2 were previously reported as active NCS enzymes^8^. The unpublished sequences for AmNCS1 and AmNCS2 were deposited in NCBI database (GenBank accession numbers ACJ76785.1 and ACJ76787, respectively). PBRNCS and PBRNCS1 were previously reported as putative NCS enzymes^42^.

Alignment of single-domain polypeptides, and the duplicated regions of multiple-domain proteins, revealed an absolute conservation of all four catalytic residues^14^, and several other amino acids, in all active NCS enzymes (Supplemental Fig. 3). Some precisely conserved residues in active enzymes were substituted in inactive proteins, notably CCHNCS5 and PBRNCS3. A schematic representation of the amino acid sequence alignment containing all tested single-domain proteins, together with the predicted translation products of additional NCS candidates, revealed three general architectural groups (Fig. 5). Several proteins (e.g. TFLNCS) included an N-terminal regional corresponding to a characterized signal peptide^29^,^31^, whereas this domain was lacking in an approximately equal number of other candidates (e.g. SCANCS1). A smaller number of proteins displayed an N-terminal extension beyond the putative signal peptide (e.g. CMANCS1). Among functionally tested proteins containing a full complement of conserved residues, polypeptides lacking the signal peptide domain were consistently active (Fig. 4). Proteins containing a putative signal peptide were generally inactive, but two of these could be substantially activated by cleavage of the first 25 amino acids of the nascent polypeptide. Notable exceptions were NDONCS3 and PSONCS1^8^, which were active without cleavage of the signal peptide. The retention of a signal peptide has been associated with thermodynamic destabilization, reducing enzyme activity and increasing protein aggregation^32^. Finally, proteins with an N-terminal extension beyond the signal peptide domain were consistently inactive.

Two active single-domain enzymes (i.e. NDONCS3 and TFLNCS2Δ25) along with the four functional multi-domain proteins (i.e. CCHNCS2, SDINCS1, PBRNCS5 and PSONCS3) were selected for further characterization. Despite considerable effort to obtain soluble recombinant proteins, purified SDINCS1, PBRNCS5 and PSONCS3 could not be obtained in sufficient quantities to perform kinetic analyses. However, NDONCS3, TFLNCS2Δ25 and CCHNCS2 were purified to near homogeneity (Supplemental Fig. 4), and were shown to display Michelis-Menten kinetics for dopamine at saturating concentration of 4-HPAA (Supplemental Fig. 5). In contrast, sigmoidal kinetics were observed for 4-HPAA at saturating concentrations of dopamine with a Hill coefficient of >1 (Supplemental Fig. 5; Table 1). The *K*_cat_ and *K*_m_ values for dopamine and 4-HPAA of the two single-domain enzymes were similar, whereas the *K*_cat_ values for both substrates of the double-domain fusion (i.e. CCHNCS2) were more than two-fold higher compared with those for NDONCS3 and TFLNCS2Δ25 (Table 1). The *K*_m_ value for dopamine of CCHNCS2 was only marginally higher than those of the single-domain enzymes. However *K*_m_ value for 4-HPAA was four-fold higher compared with the *K*_m_ values for NDONCS3 and TFLNCS2Δ25. The similarities and differences in kinetic parameters are notable in the context of the amino acid sequence identity among the three tested enzymes. NDONCS3 and TFLNCS2Δ25 share 54% amino acid sequence identity, whereas each enzyme shares only 33–35% identity with the individual domains of CCHNCS2 (Supplemental Fig. 1).

### Expression of NCS candidates in *Saccharomyces cerevisiae*

**Figure 6:**
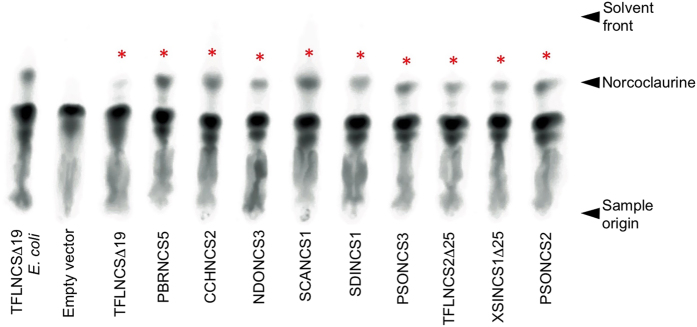
Functional analysis of 10 recombinant NCS enzymes from different plant species produced in *Saccharomyces cereviseae*. NCS activity was detected in soluble protein extracts from *S. cereviseae* cultures expressing all 10 recombinant proteins. Crude soluble protein from *S. cereviseae* producing each protein was assayed for NCS activity using [8-^14^C]-dopamine and 4-HPAA. Affinity-purified, recombinant TFLNCSΔ19 produced in *E. coli* and a total protein extract from *S. cereviseae* harboring the empty pESC-leu vector were used as positive and negative controls for NCS activity. The [8-^14^C]-norcoclaurine reaction product (labeled with asterisks) was separated using thin layer chromatography and visualized by autoradiography.

Immunoblot analysis showed the production of soluble polypeptides in *S. cerevisiae* corresponding to all eight NCS variants and two previously reported enzymes (i.e. TFLNCSΔ19 and PSONCS2)^7^,^8^ (Supplemental Fig. 2B). All recombinant proteins displayed molecular weights in agreement with the size of predicted translation products, including two with double, one with triple, and one with quadruple NCS domains. All ten enzymes assayed in crude *S. cerevisiae* protein extract converted [^14^C]-dopamine and 4-HPAA to [^14^C]-norcoclaurine (Fig. 6A). Negative control reactions containing crude soluble proteins from *S. cerevisiae* harboring the empty expression vector did not yield detectable levels of [^14^C]-norcoclaurine (Fig. 6A). Interestingly, the activity of TFLNCSΔ19 was substantially lower than that of other NCS variants in yeast protein extracts.

### Performance of NCS variants in engineered *Saccharomyces cerevisiae*

**Figure 7:**
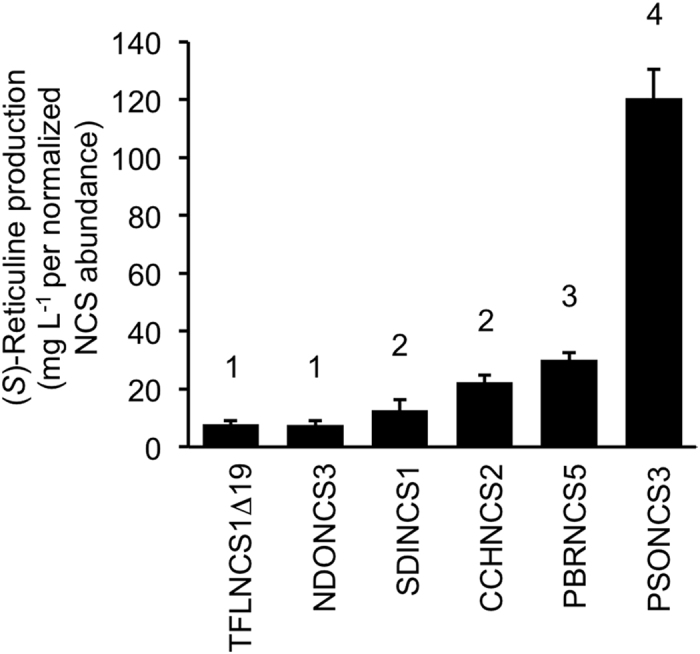
Production of (*S*)-reticuline from DOPA provided exogenously to engineered yeast strains expressing different NCS variants. Genes encoding DOPA decarboxylase (DODC), norcoclaurine 6-*O*-methyltransferase (6OMT), coclaurine *N*-methyltransferase (CNMT), and 3′-hydroxy-*N*-methylcoclaurine-4′-*O*-methyltransferase 2 (4’OMT2) were stably integrated into the yeast genome. Genes encoding a monoamine oxidase (MAO) and the NCS variants were transiently expressed from a pESC vector. The level of (*S*)-reticuline produced by each strain was measured in the culture medium. The relative abundance of each NCS variant was determined by performing immunoblot analysis on total protein extracted from a standard volume of each culture adjusted to consistent cellular density. (*S*)-Reticuline production was normalized to the level of NCS in TFLNCSΔ19, which was arbitrarily set at a value of 1. Values represent the mean ± standard deviation of 4 independent replicates.

Co-expression of *MAO* and individual *NCS* variants in engineering yeast containing chromosomally intergrated *DODC, 6OMT, CNMT* and *4*′*OMT2* genes established strains capable of converting exogenous L-DOPA to (*S*)-reticuline (Supplemental Fig. 6A). Twenty-four hours post-induction, the yield of (*S*)-reticuline was approximately 4 to 11 mg L^−1^ of culture yeast, with the highest and lowest levels produced by strains containing the gene encoding PBRNCS5 and CCHNCS2, respectively. Corresponding western blot analysis showed substantial differences in the abundance of each recombinant NCS variant, with the highest and lowest levels in strains expressing genes for NDONCS3 and PSONCS3, respectively (Supplemental Fig. 6B). Recombinant protein degradation was associated with all four repeated-domain variants, especially PBRNCS5 and PSONCS3. Normalization of (*S*)-reticuline yield with respect to the relative abundance of non-degraded NCS protein, with NDONCS3 set arbitrarily at a value of 1.0, showed that the catalytic performance of each variant positively correlated with the number of repeated domains (Fig. 7). When normalized to the relative abundance of NDONCS3, the four-domain PSONCS3 variant produced the highest level of (*S*)-reticuline. The three-domain PBRNCS5 and the two-domain proteins, SDINCS1 and CCHNCS2, showed progressively lower normalized production of (*S*)-reticuline, but all were higher than levels obtained with the single-domain enzymes TFLNCSΔ19 and NDONCS3.

## Discussion

A survey of NCS orthologs in available transcriptome databases of diverse BIA-producing plant species^28^ was pursued on the basis that novel NCS variants might potentially enhance the relatively low yield of (*S*)-norcoclaurine-derived alkaloids in engineered microorganisms^20^,^21^,^22^. An unexpected outcome of this survey was the detection of several genes encoding fusion proteins with tandem repeats consisting of 70–170 residues. In some fusions, the repeated domains contained highly conserved amino acids found in all active single-domain proteins, including four identified catalytic residues^14^. However, other fusions consisted of repeated domains that did not include complete sets of catalytic residues and were inactive. The natural occurrence of *NCS* fusions was confirmed by the amplification of cDNAs encoding proteins containing two, three or four catalytic domains. The occurrence of tandem repeats is not uncommon with approximate 14% of all proteins reported to feature duplicated domains^33^.

The exclusive detection of contigs representing tandem domain repeats in the assembled 454 and Illumina transcriptome databases of all species in the Papaveraceae, and the absence of detected multiple-domain proteins in members of the related Berberidaceae, Menispermaceae or Ranunculaceae families, suggests that the occurrence of NCS fusions is taxonomically restricted. The current absence of genomic sequence data for plants in Ranunculales order precludes an unequivocal conclusion regarding the distribution or origin of *NCS* fusions. Moreover, the number of tandem repeats detected in each amplified cDNA was dependent on the selection of RT-PCR primers, the design of which was based on available assembled RNA-seq data. Nevertheless, several important conclusions can still be drawn. First, NCS unequivocally occurs as fusions of two or more tandem domains in most, if not all, members of the Papaveraceae. Second, the differential arrangement of repeated domains and the variable length of intervening regions between the conserved catalytic units suggest an independent evolutionary origin for each fusion. Third, fusions consisting of two, three and four repeated domains were confirmed to possess NCS activity.

NCS is the second known example of an enzyme fusion involved in BIA metabolism. Recently, fusion between a cytochrome P450 (CYP) monooxygenase and an NADPH-dependent aldo-keto reductase (AKR) was shown to catalyze the *S* to *R* stereochemical inversion of reticuline as the gateway reaction to morphinan alkaloid biosynthesis in opium poppy^34^,^35^. The CYP-AKR fusion was also detected in Persian poppy (*Papaver bracteatum*), which accumulates the morphinan alkaloid thebaine. Mechanistically, the unstable iminium ion intermediate formed during the epimerization of reticuline would be prone to tautomerization, and furthermore could be oxidized to undesirable byproducts. The fusion of CYP and AKR could ensure protection of the cellular environment against reactive intermediates. However, the CYP-AKR fusion is inherently different compared with NCS fusions in that it combines two enzymes catalyzing independent conversions, whereas repeated NCS domains all catalyze the same reaction.

NCS fusions might have arisen as a mechanism to enhance the efficiency of the enzyme, as suggested from kinetic analysis of variants with single and double catalytic domains (Table 1; Supplemental Figure 4). Despite playing a pivotal role in BIA biosynthesis, NCS is a relatively inefficient catalyst, which has been suggested as a salient feature considering its role as a ‘gatekeeper’ enzyme responsible for regulating entry into alkaloid metabolism^26^. However, NCS must still generate enough (*S*)-norcoclaurine to support the production of abundant alkaloids, such as morphine, noscapine, and papaverine in opium poppy. One potential mechanism to increase enzyme function is the fusion of multiple catalytic domains, each of which exhibits ‘gatekeeper’ qualities such as low substrate affinity (i.e. high *K*_m_), but collectively operate as a single polypeptide with increased catalytic efficiency (i.e. high *K*_cat_) proportional to the number of sequentially repeated domains. Kinetic data were measured using CD spectroscopy to detect the chirality of the norcoclaurine reaction product. A racemic mixture of norcoclaurine enantiomers produced non-enzymatically^30^ does not display a CD signal; thus, any spontaneous background condensation of dopamine and 4-HPAA will not affect kinetic values determined by CD spectroscopy. The measured *K*_m_ values for dopamine and HPAA are consistent with our previous results^7^,^10^, but are substantially divergent from those in a recent report highlighting the inefficiency of NCS and potential solutions for metabolic engineering applications^26^. Comparison of the 4-HPAA and dopamine *K*_cat_ values for CCHNCS2, containing two fused NCS domains, with those of NDONCS3 and TFLNCSΔ25, each possessing only single domains, is also in agreement with the proposed proportional increase in the catalytic efficiency of NCS fusions.

The crystallographic structure of TFLNCS showed that the entrance to the catalytic cavity is formed by Y^108^, Y^131^, Y^139^ and E^103 ^14. Sequence alignment of single-domain and individual repeated regions of multiple-domain proteins (Supplemental Fig. 3) showed the absolute conservation of Y^108^ and ^Y139^ in all fourteen NCS polypeptides. However, Y^131^ was replaced by F^136^ in XSINCS1, whereas E^103^ was substituted with Q^98^ in XSINCS1 and V^95^ in NDONCS3, and by alanine in five other active enzymes, indicating some flexibility with respect to the residues that form the opening to the catalytic tunnel. The four proposed catalytic residues in TFLNCS (i.e. Y^108^, E^110^, K^122^ and D^141^) were absolutely conserved in all active NCS peptides reported in this work. Conservation of additional residues in active NCS variants might provide an extension to the salient features in PR10/Bet v1 proteins associated with NCS activity. For example, despite relaxed substrate specificity for the native substrate 4-HPAA, TFLNCS in unable to convert various α-substituted aldehyde analogs owing to the distal proximity of I^143^ to the α carbon of 4-HPAA^16^. Interestingly, I^143^ is conserved in all active single- and multiple-domain variants of NCS (Supplemental Fig. 3), suggesting that I^143^ could be a target to engineer broader substrate specificity for aldehyde substrates.

Genes encoding NCS appear to have been the target for substantial and potentially independent duplication in the genomes of BIA-producing plants. In the Papaveraceae, gene duplication has apparently resulted in the emergence of active fusion proteins with higher enzyme efficiency proportional with the number of repeated catalytic domains. However, multiple-domain fusions of NCS could represent an evolutionary mechanism to enhance the catalytic efficiency of an enzyme that is potentially recalcitrant to improved catalytic performance via protein engineering. The low amino acid sequence identity among active enzymes from different plant families suggests that natural genetic variation has not resulted in a substantial improvement in the kinetic parameters of NCS. A marked improvement in catalytic efficiency among three enzymes (i.e. NDONCS3, TFLNCS2Δ25, and CCHNCS2) appears to correlate only with the number of fused catalytic domains (Table 1). The common occurrence of *NCS* orthologs encoding inactive variants, including fusions lacking key catalytic domains, suggests an error-prone gene duplication process or low selection pressure to maintain the functional integrity of gene family members. A wide range in ploidy levels among members of the Papaveraceae might also account for the large variation in *NCS* sequences.

One initial objective of this study was the isolation of superior NCS variants as alternatives to the enzymes (e.g. TFLNCSΔ19 and PSONCS2) previously used in synthetic biology applications^20^,^21^,^22^. Interestingly, TFLNCSΔ19 showed low activity in yeast whereas all other NCS variants yielded consistently higher levels of [^14^C]-norcoclaurine in soluble yeast protein extracts (Fig. 6). Variations in NCS activity could result from differences in transcript or protein abundance, or from one of several forms of post-translational modification in yeast. The discovery of enzyme variants with improved functional characteristics in heterologous hosts is generally an empirical process. However, the availability of correlative information linking amino acid sequence, protein architecture and functional performance could substantially streamline the selection of candidates.

A positive correlation between the number of repeated domains and the apparent catalytic performance of NCS has important implications for the engineering of BIA metabolism in microorganisms. With a relatively high *K*_m_ for both dopamine and 4-HPAA, and potential limitations in cellular substrate concentrations owing to the chemical reactivity and possible toxicity of these compounds, strategies to increase BIA production in yeast should benefit from an increase in the catalytic efficiency of NCS. The natural fusion of multiple catalytic domains as an apparent evolutionary adaptation to enhance (*S*)-norcoclaurine formation in plants could be deployed for a similar purpose in microorganisms. The ability of NCS variants with repeated catalytic domains to facilitate the biosynthesis of (*S*)-reticuline in yeast cultures with yields similar to those obtained using single-domain proteins, but in some cases with substantially reduced cellular levels of NCS, is in agreement with the progressively higher catalytic efficiencies associated with the number of fused enzymatic subunits. Increasing the steady-state abundance of multiple-domain NCS variants in engineered yeast could increase the reported low yield of (*S*)-reticuline in yeast^20^,^21^ and, subsequently, facilitate the improved production of derived metabolites such as morphine^23^ and noscapine.

## Methods

### Transcriptome resources

Assembled transcriptome databases based on Roche-454 and Illumina GA and HiSeq sequencing are available from the PhytoMetaSyn Project (www.phytometasyn.ca) for 20 BIA-producing species from four plant families: Papaveraceae – *Papaver bracteatum* (PBR), *Sanguinaria canadensis* (SCA), *Chelidonium majus* (CMA), *Stylophorum diphyllum* (SDI), *Eschscholzia californica* (ECA), *Glaucium flavum* (GFL), *Argenome mexicana* (AME), and *Corydalis cheilanthifolia* (CCH); Ranunculaceae – *Thalictrum flavum* (TFL), *Hydrastis canadensis* (HCA), *Nigella sativa* (NSA), and *Xanthorhiza simplicissima* (XSI); Berberidaceae – *Berberis thumbergii* (BTH), *Mahonia aquifolium* (MAQ), *Jeffersonia diphylla* (JDI), and *Nandina domestica* (NDO); and Menispermaceae – *Menispermum canadense* (MCA), *Cocculus trilobus* (CTR), *Tinospora cordifolia* (TCO), and *Cissempelos mucronata* (CMU)^28^. Assembled transcriptome databases based on Roche-454 sequencing are also available for eight opium poppy (*Papaver somniferum*, PSO) cultivars^36^.

### Selection of NCS candidates

Full-length *NCS* candidate genes were identified through BLASTx analysis of PhytoMetaSyn Project databases using *Papaver somniferum* NCS1 (PSONCS1) and *Thalictrum flavum* NCS (TFLNCS) as query sequences. Repetitive patterns were calculated using the RADAR (Rapid Automatic Detection and Alignment of Repeats, www.ebi.ac.uk/Tools/pfa/radar) algorithm^37^. First-strand cDNA synthesis was performed on total RNA isolated from each plant species using Moloney murine leukemia virus reverse transcriptase and an oligo-dT primer. Double-strand cDNAs encoding full-length NCS candidates were amplified by PCR (30 cycles each consisting of 94 °C for 30 sec, 52 °C for 30 sec, and 72 °C for 2 min) using specific forward and reverse primers (Table S1). PCR reactions contained dNTPs at a concentration of 0.3 mM each, 0.3 mM of each primer, 50 ng of template DNA, 5 x KAPA Hifi reaction buffer, and KAPA Hifi DNA polymerase (Kapa Biosystems). Amplicons were cloned in the pGEM-T easy vector and used as a template for subsequent PCR under the same conditions. To clone the full-length coding region of native *NCS* candidate genes into the pET29b expression vector, primers were designed to include flanking 5′ *Hin*dIII and 3′ *Bam*HI or *Xho*I restriction sites (Table S2). A synthetic gene encoding full-length SDINCS1 was codon-optimized for expression in *Escherichia coli*. Truncated genes encoding NCS candidates lacking the first 25 amino acids of the nascent protein were generated using forward primers annealing to sequences 75 nucleotides from the native transcription start sites (Table S3). Amplicons were inserted into between *Hin*dIII and *Bam*HI or *Xho*I restriction sites of pET29b using T4 DNA ligase (Invitrogen). Ligations were used to transform *E. coli* strains BL21 pLysS or ER2566 pLysS.

### Expression of NCS candidates in *Escherichia coli*

Fresh overnight *E. coli* cultures harboring pET29b expression constructs encoding full-length or truncated NCS candidate enzymes were used to inoculate 250 mL of lysogeny broth (LB) medium containing kanamycin (50 μg/mL) and chloroamphenicol (35 μg/mL). Cultures were grown at 37 °C to an *A*_600_ of 0.5 with shaking. Subsequently, isopropyl β-D-thiogalactoside (IPTG) was added to a final concentration of 0.5 mM. Culture growth was continued for 4.5 h at 37 °C. For the production of soluble PSONCS3, cultures were induced with 0.5 mM IPTG overnight at 4 °C. Bacteria were harvested by centrifugation at 8000 × *g* for 10 min and re-suspended in cold 20 mM Tris, pH 7.5, 100 mM KCl, 10% (v/v) glycerol. Cells were disrupted by sonication, and proteins were separated into soluble and insoluble fractions by centrifugation.

### Expression of NCS candidates in *Saccharomyces cerevisiae*

The synthetic SDINCS1 gene included a C-terminal His_6_-tag and was flanked by *Not*I and *Sac*I restriction sites for direct insertion into the pESC-leu2d yeast expression vector (Agilent). C-terminal His_6_-tags were fused to other NCS candidates by re-amplifying NCS gene candidates by PCR using reverse primers that included sequences encoding the His_6_-tag (Table S4). Amplicons were ligated into pESC-leu2d using *Not*I and *Bgl*II, *Not*I and *Spe*I, *Spe*I and *Pac*I, or *Not*I and *Sac*I restriction enzyme sites, and the resulting yeast expression vectors were used to transform *Saccharomyces cerevisiae* strain YPH 499^38^. A single transformed yeast colony was used to inoculate 2 mL of synthetic complete (SC) medium lacking leucine, but containing 2% (w/v) glucose, and grown overnight at 30 °C and 200 rpm. A flask containing 50 mL of SC medium lacking leucine, but containing 1.8% (w/v) galactose, 0.2% (w/v) glucose and 0.1% (w/v) raffinose, was inoculated with 1 mL of the overnight culture and grown at 30 °C and 200 rpm for approximately 55 h. Yeast cells were collected by centrifugation and suspended in 3 mL of 50 mM phosphate buffer, pH 7.3. Cells were lysed by sonication, cell debris was removed by centrifugation at 20,000 × *g* at 4 °C for 30 min, and the supernatant was used for enzyme assays.

### SDS-PAGE and immunoblot analysis

Recombinant proteins were fractionated by SDS-PAGE using a 12% (w/v) polyacrylamide gel. Immunoblot analysis was performed using an anti-His_6_ antibody and chemoluminescent detection with SuperSignal West Pico substrate (Thermo Scientific). Blots were developed on Kodak XAR film.

### NCS enzyme assays

NCS enzyme assays were performed as described previously^8^. Briefly, reaction mixtures containing recombinant protein, 1 nmol [8-^14^C]-dopamine and 10 nmol 4-HPAA were incubated for 1.5 h at 37 °C. Reactions were subsequently spotted onto silica gel 60 F_254_ TLC plates and developed in *n*-butanol:acetic acid:water (4:1:5, v/v/v). Autoradiogram visualization and analysis was performed using a Molecular Imager PharosFX system and Quantity One software (Bio-Rad).

### Purification of NCS candidate proteins from *Escherichia coli*

NDONCS3, TFLNCS2Δ25, CCHNCS1 ligated into the *Nde*I and *Xho*I sites of pET29b vector were transformed into the *E. coli* strain Rosetta (DE3). A fresh overnight culture was used to inoculate 250 mL of LB medium containing kanamycin (50 μg/mL) and chloroamphenicol (35 μg/mL). The culture was grown at 37 °C to an *A*_600_ of 0.5 with shaking. IPTG was subsequently added to a final concentration of 0.5 mM and cultures were grown for an additional 15–17 h at room temperature. Cells were harvested by centrifugation at 11,300 × *g* for 20 min at 4 °C. All purification steps were performed at 0–4 °C. Approximately 2–3 g of the cellular pellet was suspended in 40 mL of buffer A (50 mM potassium phosphate, pH 7.5, 50 mM NaCl, 5 mM imidazole) containing lysozyme (1 mg/mL) and DNase I (5 μg/mL). After incubation for 15 min at room temperature and an additional 20 min on ice, the cell suspension was lysed by sonication. Cellular debris was removed by centrifugation at 20,000 × *g* for 30 min at 4 °C. The supernatant was mixed with approximately 1 mL of TALON metal affinity resin (Clontech), shaken gently for 20 min and loaded onto a fritted column (1 × 15 cm). The flow through was removed and 30 mL of buffer A was used to wash out weakly bound contaminant proteins. The target protein was recovered by consecutive elution using 10 mL of buffer A containing 10–100 mM imidazole at a gravity-controlled flow rate. Fractions containing pure protein, as determined by SDS-PAGE, were collected, dialyzed against phosphate buffer (50 mM sodium phosphate, pH 7.5) and stored at −20 °C. Protein concentration was determined by absorbance at 280 nm.

### Kinetic analysis of purified NCS enzymes

Enzyme kinetic data was obtained by monitoring the formation of (*S*)-norcoclaurine using circular dichroism (CD) spectroscopy^10^. The data was processed in the program GraphPad prism. Briefly, the experiments were performed on a Jacsco J-715 spectropolarimeter (JASCO Corporation), by monitoring the increase of ellipticity at 285 nm, which corresponds to the formation of (*S*)-norcoclaurine. All the reactions were monitoring in a 0.1 cm quartz cell. Molar ellipticity of 12,541 mdeg cm^−1^ M^−1^ was used to calculate the concentration of (*S*)-norcoclaurine^10^. Assays were carried out at 37 °C in 50 mM phosphate buffer (pH 7.3) at a various concentrations of dopamine and 4-HPAA (100–2000 μM).

### ESI[+]-MS spectrum of (*S*)-norcoclaurine

Assay mixtures in a final volume of 50 μL, containing 2 mM dopamine, 2 mM 4-HPAA and 40 μL of crude protein extract in 50 mM phosphate buffer (pH 7.3), were incubated at 37 °C for 2 h. Reactions were quenched with 5 μL of 6 M HCl and the mixture was diluted with 0.6 M HCl to a final volume of 1 mL. Following filtration through a 0.22 μM Millex-GV (Millipore) filter to remove proteins, 20 uL of the reaction mixtures was diluted to 100 μL in water and analyzed by ESI[+]-MS using an Agilent 6410 triple-quadrupole mass spectrometer.

### Phylogenetic analysis

Sequence editing, sequence alignments and a phylogenetic tree obtained from PHYML analyses were performed with the Geneious Pro 5.6 software package (Biomatters). ClustalW2 was used to perform multiple sequence alignments of the amino acid sequences associated with NCS candidates and query sequences^39^. A consensus tree was generated from 100 samples to obtain bootstrap values.

### Cloning and expression of NCS variants in engineered *Saccharamyces cerevisiae*

A *Saccharomyces cerevisiae* strain harboring chromosomally integrated alkaloid biosynthetic genes operating upstream and downstream of NCS was constructed. Genes encoding DOPA decarboxylase (DODC) from *Pseudomonas putida*, and norcoclaurine 6-*O*-methyltransferase (6OMT), coclaurine *N*-methyltransferase (CNMT), and 3′-hydroxy-*N*-methylcoclaurine 4′-*O*-methyltransferase 2 (4′OMT2) from *Papaver somniferum* were used to transform *S. cerevisiae* stain CENPK102-5B (MATα, ura3-52, his3D1, leu2-3/112, MAL2-8c, SUC2)^40^. The LiAc/PEG/single-stranded carrier DNA (ssDNA) transformation method^38^ was used as described previously^41^ To achieve plasmid-based NCS and monoamine oxidase (MAO) expression without compromising auxotrophic selection of chromosomally integrated genes, a modified version of pESC-his (Agilent) was created. An expression cassette for the KanMX4 marker was inserted immediately downstream of the HIS3 cassette to allow G418 antibiotic selection. Synthetic, codon-optimized, and HA-tagged *Homo sapiens* MAO was cloned into MCS2 of this modified vector, renamed pEV-MAO, using *BamH*I and *Sac*II. Codon-optimized synthetic genes encoding full-length coding regions of each *NCS* variant, fused to C-terminal His_6_ tags, and flanked by 5′ *Not*I *and* 3′ *Sac*I restriction sites, were inserted into MCS1 of pEV-MAO behind the *Gal1* promoter. The resulting pEV-MAO plasmids containing NCS variants were transformed to the yeast strain harboring chromosomally integrated BIA biosynthetic genes.

### Engineered *Saccharamyces cerevisiae* culture and substrate feeding

Four independent colonies of each yeast strain transformed with pEV-MAO constructs containing *NCS* variants were used to inoculate individual wells of a 96-well plate, with each well containing 600 μL of SD*–His–Ura–Leu* medium supplemented with 2% (w/v) glucose. Yeast cultures were grown overnight at 30 °C on a gyratory microplate shaker at 900 rpm. Subsequently, 30 μL of the original culture was transferred to 600 μL of SD*–His–Ura-Leu* containing 2% (w/v) galactose and 1 mM L-DOPA and cultures were grown at 30 °C for 24 h on a gyratory microplate shaker at 900 rpm. For analysis, 5 μL of the supernatant from each culture were subjected to high-resolution mass spectrometry analysis. Yeast cultures were adjusted to a consistent cellular density and volume, using fresh culture medium when required, and cells were collected directly in the 96-well culture plate by centrifugation. Pelleted cells were re-suspended in SDS-PAGE loading buffer and boiled for 5 min and 5 μL of lysate were subjected to immunoblot analysis using an anti-His_6_ antibody and chemoluminescent detection with SuperSignal West Pico substrate (Thermo Scientific). Blots were imaged using a GE Amersham Imager 600 (GE Healthcare Life Sciences). Relative western blot signal intensities of bands corresponding to the expected size of non-degraded NCS polypeptides were determined by digital densitometric analysis using Image Studio Lite software (LI-COR Biosciences). Liquid chromatography methods and LTQ-Orbitrap-XL mass spectrometric analysis of alkaloids was performed as described previously^41^.

## Additional Information

**How to cite this article**: Li, J. *et al*. Genes encoding norcoclaurine synthase occur as tandem fusions in the Papaveraceae. *Sci. Rep.* **6**, 39256; doi: 10.1038/srep39256 (2016).

**Publisher's note:** Springer Nature remains neutral with regard to jurisdictional claims in published maps and institutional affiliations.

## Supplementary Material

Supplementary Information

## Figures and Tables

**Figure 1:**
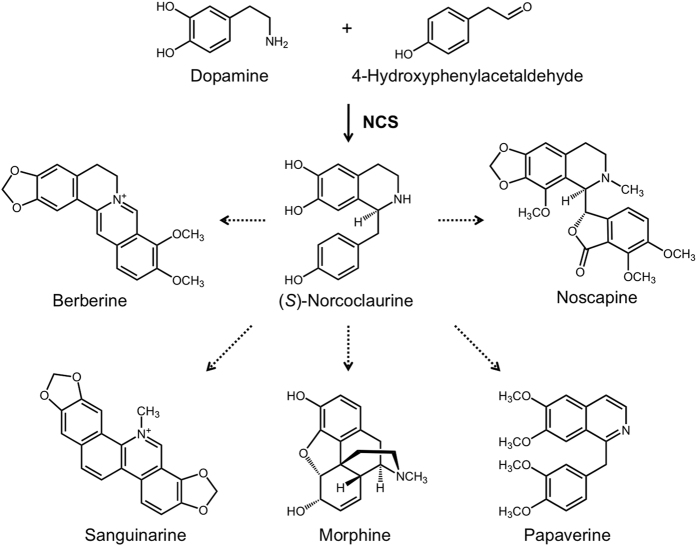
Condensation of dopamine and 4-hydroxyphenylacetaldehyde by NCS yields (*S*)-norcoclaurine, which is the central intermediate in the biosynthesis of structurally diverse benzylisoquinoline alkaloids including berberine, sanguinarine, morphine, noscapine and papaverine.

**Table 1:** Kinetics of NCS variants from *Nandina domestica* (NDONCS3), *Thalictrum flavum* (TFLNCSΔ25) and *Corydalis chinensis* (CCHNCS2).

Enzyme	Domain repeats	*k*_cat_ (s^−1^) (4-HPAA)	*k*_cat_ (s^−1^) (dopamine)	Hill coefficient (dopamine)	*K’*_m_ (μM) (dopamine)	*K*_m_ (μM) (4-HPAA)
NDONCS3	1	1.7 ± 0.1	2.8 ± 0.1	1.9 ± 0.4	347 ± 35	480 ± 120
TFLNCS2Δ25	1	2.4 ± 0.2	2.1 ± 0.6	1.6 ± 0.2	380 ± 29	494 ± 112
CCHNCS2	2	5.3 ± 1.0	5.8 ± 0.6	1.7 ± 0.3	502 ± 82	1829 ± 697
