# Supplementary material for: Genes encoding norcoclaurine synthase occur as tandem fusions in the Papaveraceae
Source: Sci Rep. 2016 Dec 19;6:39256. doi: 10.1038/srep39256 (PMC5171800; doi:10.1038/srep39256)
Supplement: Supplementary Information [file srep39256-s1.pdf]

SUPPLEMENTAL INFORMATION

Genes encoding norcoclaurine synthase occur as tandem fusions in the Papaveraceae

Jing Li, Eun-Jeong Lee, Limei Chang and Peter J. Facchini

Department of Biological Sciences, University of Calgary, Calgary, Alberta T2N 1N4, Canada

	CCHNCS2 (2)	CCHNCS5	CMANCS1	ECANCS1	NDONCS3	PBRNCS3	PBRNCS4	PBRNCS5 (3)	PSONCS3 (4)	SCANCS1	SDINCS1 (2)	TFLNCS2	XSINCS1	TFLNCS
CCHNCS1 (2)	78	56	73	62	36	61	66	68	56	70	75	39	35	36
CCHNCS2 (2)		50	62	63	33	52	58	61	54	60	67	35	32	35
CCHNCS5			55	52	53	49	47	48	41	55	55	48	44	48
CMANCS1				62	38	67	74	73	56	89	96	37	34	37
ECANCS1					35	57	60	59	48	62	63	35	32	36
NDONCS3						36	35	36	30	54	38	54	51	55
PBRNCS3							76	75	56	72	68	33	29	34
PBRNCS4								94	61	75	74	35	30	35
PBRNCS5 (3)									74	75	77	35	31	35
PSONCS3 (4)										51	61	31	26	31
SCANCS1											90	50	46	50
SDINCS1 (2)												37	34	37
TFLNCS2													59	88
XSINCS1														59

Supplemental Figure 1. Amino acid sequence identity among candidate proteins functionally tested for NCS activity. Values represent the percentage identity of single domain proteins, or of individual domains in fusion proteins with tandem repeated polypeptides. Active NCS enzymes are named in red. Numbers in brackets indicate the number of tandem repeated domains. Shading highlights sequences with 25-49% identity (green), 50-74% identity (yellow) and 75-100% identity (red).

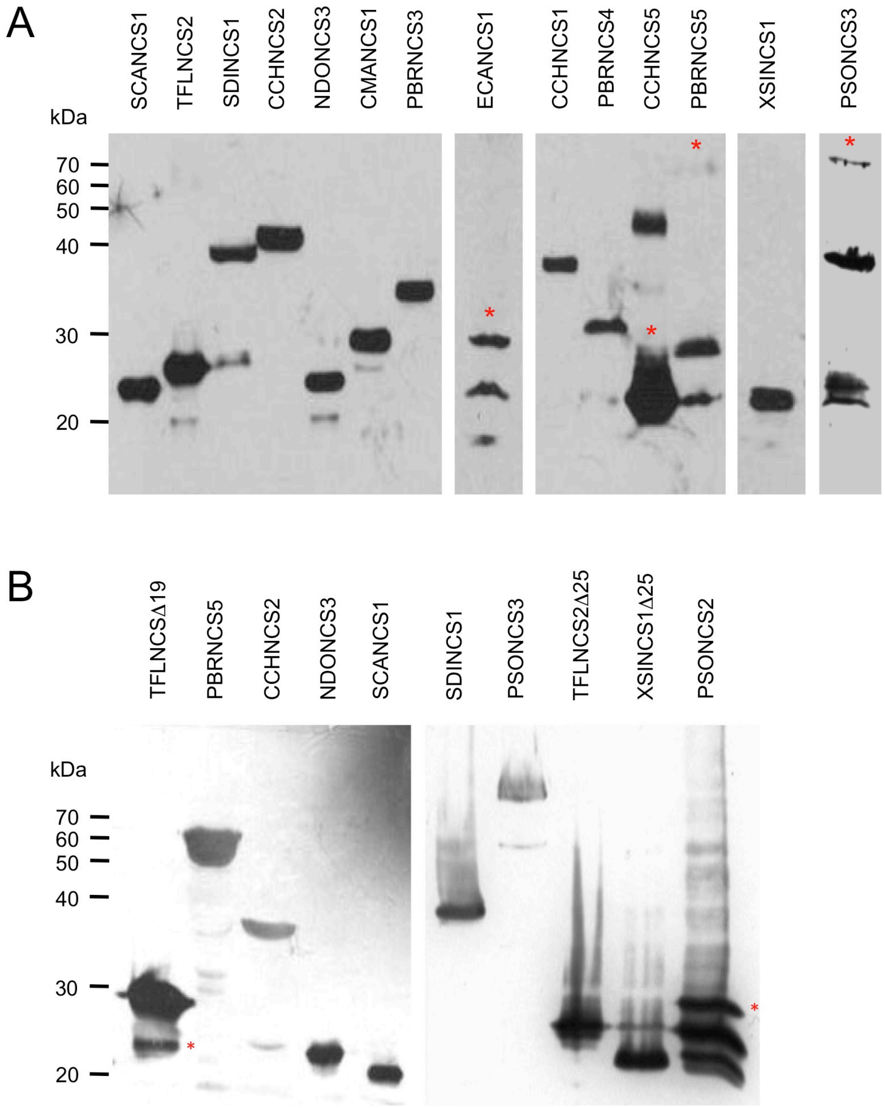

Supplemental Figure 2. Heterologous expression of candidate NCS enzymes from different plant species. A, Immunoblot blot analysis performed using a His_6-tag monoclonal antibody showing the occurrence of 14 soluble recombinant NCS candidates in total protein extracts of *Escherichia coli* cultures harboring corresponding pET29b expression constructs. B, Immunoblot blot analysis performed using a His_6-tag monoclonal antibody showing the occurrence of soluble recombinant NCS candidates in total protein extracts of *Saccharomyces cereviseae* cultures harboring corresponding pECS-leu expression constructs. When more than one protein band was detected using the His_6-tag monoclonal antibody, asterisks indicate bands consistent with the molecular weights of predicted translation products.

```
CCHNCS1-R1  ------------------------MYYFLEFFEKLDVIEGNGGVGTVLDIAFPPGAVPRS 36
PBRNCS3     ----------------------------MDIIEGDGGVGTVLDVVFQPGAVPQSYKERFE 32

CCHNCS1-R1  YKEKFVKVDHKNRLKEVVMIEGGYLDLGCTFYMDRIHVLPKGANSCVIKSTLIYEIPDEL 96
CMANCS1     ------------------------MIEGGYLDMGCTFYMDRIHVVKKGPNSCVIASAIIY 36
ECANCS1     -------------------MIGGFLDMGCTFYMDRIHVVAKGPNSCIIKSTLIYEVKEEY 41
PBRNCS3     TVDHEKRILEVRIIQGGYLEMGCTSYLNRMHVIEITSKSCVIKSSVIYDVKEECADAMSK 92
PBRNCS4     ------------MIEGGYLDMGCTFYLDRIHVVEKTPSSCVIESSIVYEVKQECAEAISK 48

PSONCS3-R1  ---------------------------------------IKYDMEVAVSADSVWAVYSSP 21
PSONCS3-R2  ----------------------SFSARNILSKQSVVKKEIRYDLEVPISADSIWSVYSCP 38
PSONCS3-R3  ----------------------SFSARNILNKNSLVKKEIRYDLEVPTSADSIWSVYSCP 38
PSONCS4-R4  ----------------------SVSERNIPKKQSLLRKEITYETEVQTSADSIWNVYSSP 38
PBRNCS5-R1  -----------------------------------------YDMEVATSADSVWAVYSSP 19
PBRNCS5-R2  ------------VISNYVIQKESVSARNIFNRQSVVKKEIHYDLEVPTSADSIWAVYSNP 48
PBRNCS5-R3  --------------ANYVLKKQSVSDTNIPKKQSVLRKEITYETEVQTSVDSIWNVYSSP 46
SDINCS1-R1  ------------------------------------RKEVRYEMEVPTSADSIWAVYSSH 24
SDINCS1-R2  ------------------------------------RKELTYEMEVPTSADSIWAVYSSH 24
CCHNCS2-R1  -----------------------------------MRKELRHELEVATSADSIWAVYGSP 25
CCHNCS2-R2  -----------------------------------LRKELTTEMEVPTSADSIWAVYGSP 25
SCANCS1     -----------------------------------MRKELTHEMEVPASADAIWAVYSSH 25
TFLNCS2Δ25  --------------------RPFLNRQGIINQVSTVTKGVHHELEVAASADDIWSVYSWP 40
TFLNCSΔ19   -------------QKLILTGRPFLHHQGIINQVSTVTKVIHHELEVAASADDIWTVYSWP 47
XSINCSΔ25   -------------------GRPLLHR--VTNEETVI---LYHELEVPASVDELWSVEGSP 36
NDONCS3     MRSGIVFLVLFFLGCEISQGRQLLES--RLFRKSTIRKVLHHELPVAASAQEVWDVYSSP 58
CCHNCS1-R1  VDSVGSLMSTEPLASMAKVISDYVLKQRKMTANKILRKELKTEMEVATSADSIWAVYGSP 156
CCHNCS1-R2  ----------------------------KKEAKKILRKELTHELEVPTSADSIWAVYGSP 32
CCHNCS5     -----------------------------------MRKELTNELEVAAPADAVWAVYSSP 25
CMANCS1     VKEEFVDVVVPLITTEPLASMAEVISNYVLKKQRRVRKELTYEMEVPTSADSIWAVYSSH 96
ECANCS1     ADAMASLITVEPLASMAEVVANYVLHQQVRVLGSVKRKELTHELEVAAPADAIWGVYSSP 101
PBRNCS3     LITTIQLESMAKVVADYVLKKQSASDTSIPKKQSLMRKEITHEMEVQTSADSIWDIYSSP 152
PBRNCS4     LITTEPLKSMAEVIANYVLKKQSVSDTNIPKKQSVLRKEITYETEVQTSVDSIWNVYSSP 108

PSONCS3-R1  DIPRLLRDVLLPGVFEKLDVIEGNGGVGTVLDIVFPPGAVPRSYKEKFVNIDREKRLKEV 81
PSONCS3-R2  DIPRLLRDVLLPGVFEKLDVIEGDGGVGTVLDIVFPPGAVPRSYKEKFVNIDREKRLKEV 98
PSONCS3-R3  DIPRLLRDVLLPGVFQKLDVIEGNGGVGTVLDIVFPPGAVPRSYKEKFVNINHEKRLKEV 98
PSONCS4-R4  DIPRLLRDVLLPGVFEKLDVIAGNGGVGTVLDIAFPLGAVRRRYKEKFVKINHEKRLKEV 98
PBRNCS5-R1  DIPRLLRDVLLPGVFEKLDVIEGNGGVGTVLDIAFPPGAVPRSYKEKFVNIDRVKRLKEV 79
PBRNCS5-R2  DIPRLLRDVLLPGVFEKLDVIEGNGGVGTVLDIVFPPGAVPRRYKEKFVNINHEKRLKEV 108
PBRNCS5-R3  DIPRLLRDVLLPGVFEKLDVIAGNGGVGTVLDIAFPLGAVPRRYKEKFVKINQEKRLKEV 106
SDINCS1-R1  DIPRLLKEVLLPGVFEKLDVIEGDGGVGTVLDIAFPPGAVPRTYKEKFVTINHEKRLKEV 84
SDINCS1-R2  DIPRLLKEVLLPGVFEELDVIEGDGGVGTVLDIAFPPGAVPRTYKEKFVKINHEKRLKEV 84
CCHNCS2-R1  DIPRLLRDVLLPGVFEKLDVIQGNGGVGTVLDIAFPPGAVPRTYKEKFVKVDHKNRLKEV 85
CCHNCS2-R2  DIPRLLRDVLLPGVFERLDVIEGNGGVGTVLDISFPPGAVPRSYKEKFVKVDHKNRLKEV 85
SCANCS1     DIPRLLKEVLLPGVFEKLDVIAGDGGVGTVLDIAFPPGAVPRRYKEKFVKINHEKRLKEV 85
TFLNCS2Δ25  GLAKHLPD-LLPGAFEKLEII-GDGGVGTILDMTFTPGEFPHEYKEKFILVDNEHRLKKV 98
TFLNCSΔ19   GLAKHLPD-LLPGAFEKLEII-GDGGVGTILDMTFVPGEFPHEYKEKFILVDNEHRLKKV 105
XSINCSΔ25   ELGKNLPD-LLPGIFADFKIT-GDGGEGSILDMTFPPGQFPHHYREKFVFFDHKNHYKLV 94
NDONCS3     ELPKHLPE-ILPGAFKKVVVT-GDGGVGTVIEMVFPPGVVPHRYKEKFVLIDDEKFLKKV 116
CCHNCS1-R1  DIPRLLRDVLLPGVFEKLDVIEGNGGVGTVLDIAFPPGAVPRTYKEKFVKVDHKNRLKEV 216
CCHNCS1-R2  DIPRLLRDVLLPGVFEKLDIVEGNGGVGTVLDIAFPPGAVPRSYKEKFVKVDHDKHLKEV 92
CCHNCS5     DLPKIIVE-LLPSVFEKIEIVEGDGGVGTVLYVVFPPGSVPLTYKEKFVTIDHEKRLKEV 84
CMANCS1     DIPRLLKEVLLPGVFEKLDVIEGDGGVGTVLDIAFPPGAVPRTYKEKFVKINHEKRLKEV 156
ECANCS1     DIPRLLRDVLLPGVFEKLEVIQGNGGVGTVLEIVFHPGAIPRRYKEKFVTINHKKRLKEV 161
PBRNCS3     DIPRLLRDVLLPGAFEKLHVIQGNGGVGTVLDIALPLGAVPRNYKEKFVKINHEKRLKEA 212
PBRNCS4     DIPRLLRDVLLPGVFEKLDVIAGNGGVGTVLDIAFPLGAVPRRYKERFVKINHEKRLKEV 168

PSONCS3-R1  IMIEGGYLDMGCTFYLDRIHVVEKTKSSCVIESSIVYDAKEECADAMSKLITTEPLK--- 138
PSONCS3-R2  IMIEGGYLDMGCTFYLDRIHVVEKSLSSCVIESSIVYEVKEEYVDAMSKLITTEPLK--- 155
PSONCS3-R3  IMIEGGYLDMGCTSYLDRIHVVEKTSKSCIIKSSVVYEVKQECVEAMSKLITTEPLK--- 155
PSONCS4-R4  VMIEGGYLDMGCTFYMDRIHVFEKTPNSCVIESSIITKLKK------------------- 139
PBRNCS5-R1  IMIEGGYLDMGCTFYLDRIHVVEKTPSSCVIESSIVYEVEEEYADVM------------- 126
PBRNCS5-R2  IMIEGGYLDMGCTFYLDRIHVVEKTSKSCIIKSSIVYEVKQECAEAI------------- 155
PBRNCS5-R3  IMIEGGYLDMGCTFYMDRIHVLEKTPNSCVIESSIIYEVKEEFADKM------------- 153
SDINCS1-R1  IMIEGGYLDMGCTFYMDRIHVLEKGPKSCIIASAIIYEVKEEFADVVVPLITTEPLASMA 144
SDINCS1-R2  VMIEGGYLDMGCTFYMDRIHVLEKGPNSCVIESAIIYEVKEEFADVVVPLITTEPLASMA 144
CCHNCS2-R1  VMIEGGYLDLGCTFYMDRIHVLPSGPNTCIIKSTLIYEVPDELAYSVASLISVEPLASMA 145
CCHNCS2-R2  VMIEGGYLDLGCTFYMDRIHVIPKGPNSCVIKSTLIYEIPGELVDSVGSLMSTEPLASMA 145
SCANCS1     EMIEGGYLDMGCTFYMDRIHVVEKGPNSCVIESAIIYVVKDECADVVVPLITTEPLASMA 145
TFLNCS2Δ25  QMIEGGYLDLGVTYYMDTIQVIPTGTNSCVIKSSTEYHVKPEFVKIVEPLITTGPLAAMA 158
TFLNCSΔ19   QMIEGGYLDLGVTYYMDTIHVVPTGKDSCVIKSSTEYHVKPEFVKIVEPLITTGPLAAMA 165
XSINCSΔ25   QMIDGDFFDLGVTYYMDTIRVVATGPDSCVIKSSTEYHVKVEFAKIVKPLIDTVPLAIMS 154
NDONCS3     EMIEGGYLDMGCTFYMDTIQIVPTGPDSCIIKSSTEYYVKPEFADKVVPLISTVPLQAMA 176
CCHNCS1-R1  VMIEGGYLDLGCTFYMDRIHVLPKGPNTCVIKSTLIYEVPDEFADAVGSLISVEPLASMA 276
CCHNCS1-R2  VMIEGGYLDLGCTFYMDRIHVLPKGPNSCVIESSLIYEVREELADVVGSLISIEPLASMA 152
CCHNCS5     LQIEGGYLDLGCTFYMDSFHILEKDCDSCIIKSITAYEVRDDVVDNVSSLISIDSLANMA 144
```

```
CMANCS1     VMIEGGYLDMGCTFYMDRIHVLEKSPNSCVIESSIIYEVKEEFADVVGPLITTEPLASMS 216
ECANCS1     VMI-GGYLDMGCTLYMDRIHVVSKGPNSCVIKSTLIYEVKAESADAMASTITIDPLASMA 221
PBRNCS3     VMIEGGYADMGCTFYMHRIHVLEKTPKSCVIESSIVYEVKEEYADKMSKLITTEPLQSMA 272
PBRNCS4     VMIEGGYLDMGCTFYMDRIHVFDKTPNSCVIESSIIYEVKEEYADKMAKLITTEPLQSMA 228

PSONCS3-R1  ------------------------------------------------------------
PSONCS3-R2  ------------------------------------------------------------
PSONCS3-R3  ------------------------------------------------------------
PSONCS4-R4  ------------------------------------------------------------
PBRNCS5-R1  ------------------------------------------------------------
PBRNCS5-R2  ------------------------------------------------------------
PBRNCS5-R3  ------------------------------------------------------------
SDINCS1-R1  EVISNYVLKKQ------------------------------------------------- 155
SDINCS1-R2  EVISNYVLKKQ------------------------------------------------- 155
CCHNCS2-R1  EVISGYVLRQRKMTTNKI------------------------------------------ 163
CCHNCS2-R2  AVISDYVLKQRKMTANQI------------------------------------------ 163
SCANCS1     EVISNYVLRKQIRLFGYVIKPKLGLSILLSLILCLVILGVLLIGGVPF------------ 193
TFLNCSΔ25   EAISKLVLEHKYKSNSDEIDASKNNLKMVINM---------------------------- 190
TFLNCSΔ19   DAISKLVLEHKSKSNSDEIEAA------IITV---------------------------- 191
XSINCSΔ25   EAIAKVVLEKKYKRSE-------------------------------------------- 170
NDONCS3     EAIAKIVLEFKAKHKG------------FIEI---------------------------- 196
CCHNCS1-R1  EVISGYVLKQ-------------------------------------------------- 286
CCHNCS1-R2  EVISSYVLKQQLRVFGVVVQPRVGLSLLLCLILCLVILGGLLIGGVSI------------ 200
CCHNCS5     EAISKYVLEKQEAATKHGHGDDRERTGLCWPFNCLG------------------------ 180
CMANCS1     EVISNYVLKKQIRMFGYVIKPKLGLSLLLCFILCLVLLGVLLIGGVPL------------ 266
ECANCS1     QVISNYVLKNQMQVLGSVKRRELTHELEVAASADAIWGVYGSKRYSKASQGCFASWCFRK 281
PBRNCS3     EAISSYVLKKQFQVFGLEVKPKLVLSLFLCLIIFLAIVGGFLIGGLKA------------ 312
PBRNCS4     EVISGYVLKKRLQVFGFEIKPNLRFNLLLCLIICLVIAGGMLIGRVPL------------ 276
```

Supplemental Figure 3. Amino acid sequence alignment of single domain proteins and individual domains in fusion proteins with tandem repeated (R) polypeptides. Names of active NCS enzymes are shown in red. Key catalytic residues are highlighted black, whereas residues perfectly conserved in active enzymes are highlighted in grey. Exceptions to the perfect conservation of residues found in active enzymes are highlighted in yellow in proteins lacking NCS activity.

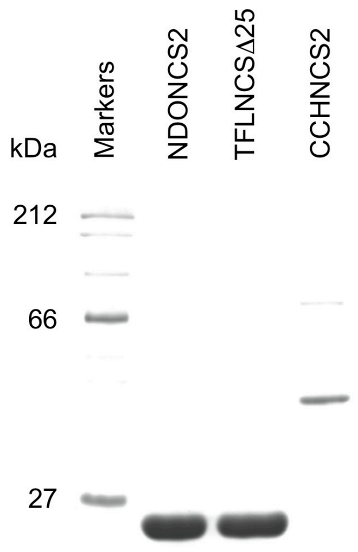

Supplemental Figure 4. SDS-PAGE of purified NDONCS3, TFLNCS2Δ25 and CCHNCS2 enzymes used for kinetics analyses.

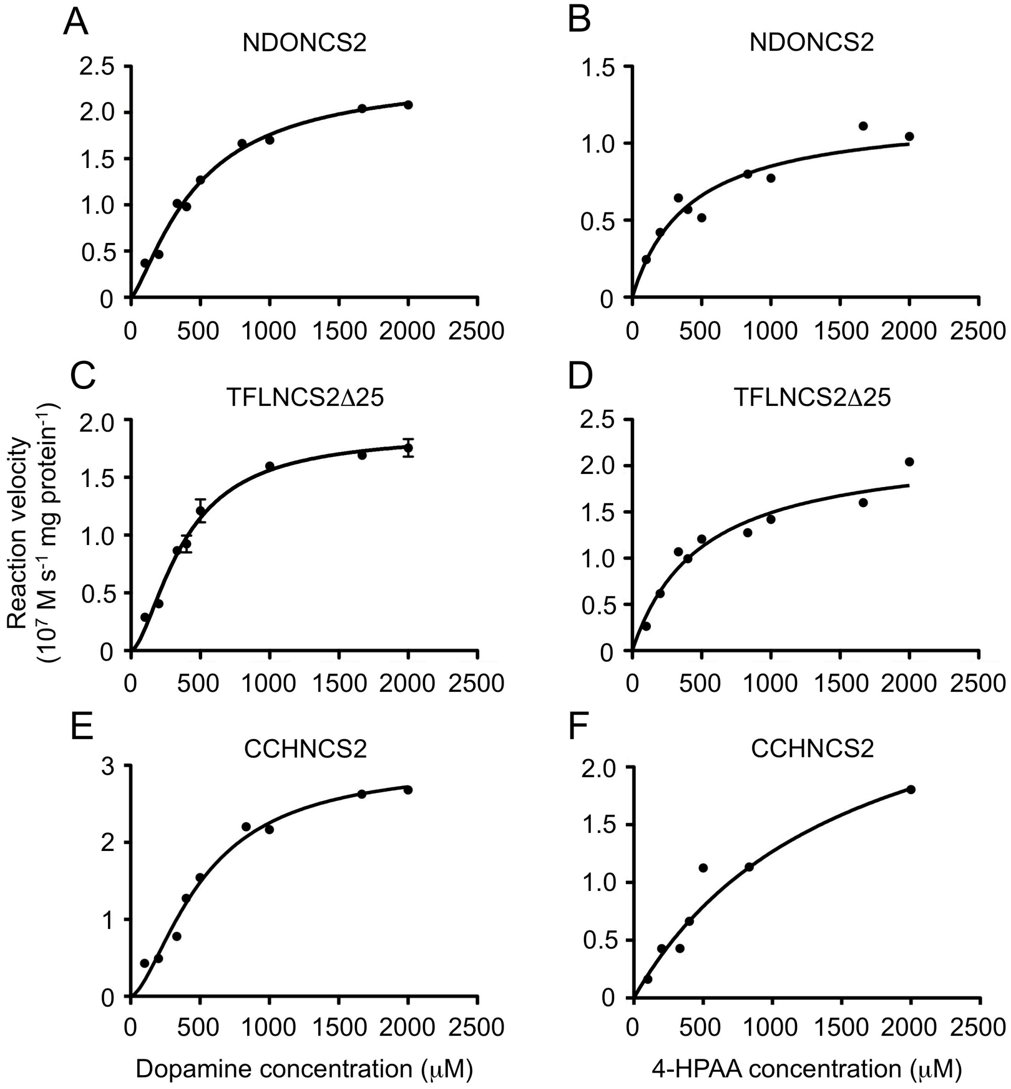

Supplemental Figure 5. Steady-state enzyme kinetics of affinity-purified recombinant NDONCS2 (A and B), TFLNCSΔ25 (C and D) and CCHNCS2 (E and F) for dopamine (A,C,E) and 4-hydroxyphenylacetaldehyde (4-HPAA) (B,D,F). Kinetic parameters were determined by varying the concentration of one substrate at a fixed saturating concentration of the other substrate under otherwise standard assay conditions. Kinetic constants were determined by fitting initial velocity versus substrate concentration to the Hill equation.

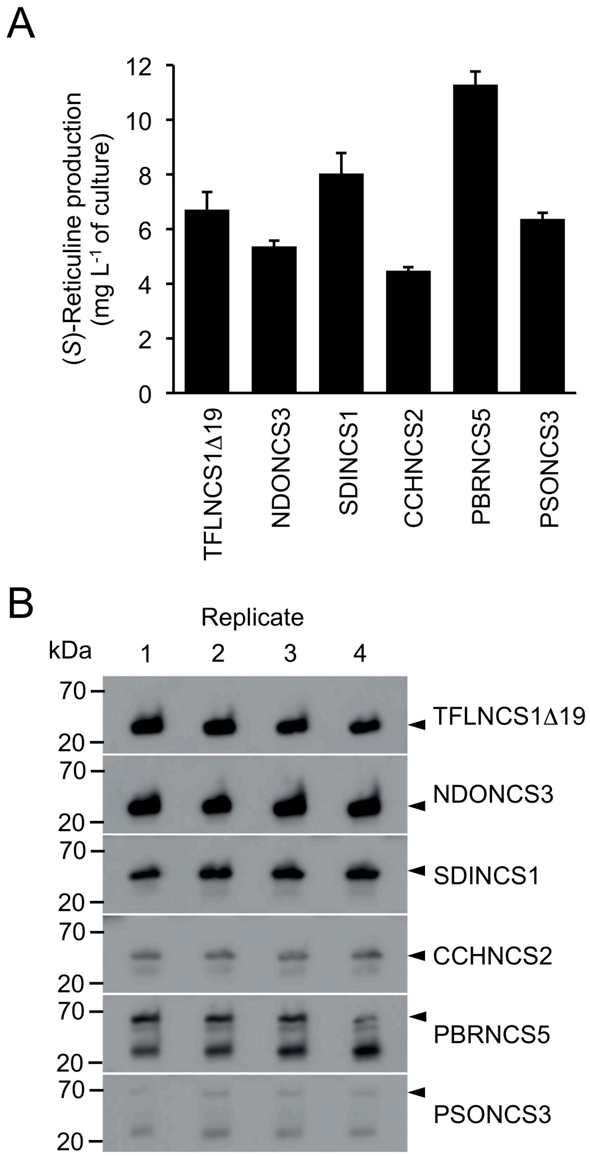

Supplemental Figure 6. Production of (*S*)-reticuline from DOPA provided exogenously to engineered yeast strains expressing different NCS variants. Genes encoding DOPA decarboxylase, norcoclaurine 6-*O*-methyltransferase, coclaurine *N*-methyltransferase, and 3'-hydroxy-*N*-methylcoclaurine-4'-*O*-methyltransferase were stably integrated into the yeast genome. Genes encoding a monoamine oxidase and the NCS variants were transiently expressed from a pESC vector. The level of (*S*)-reticuline produced by each strain was measured in the culture medium (A). The relative abundance of each NCS variant was determined by performing immunoblot analysis on total protein extracted from a standard volume of each culture adjusted to consistent cellular density. Arrowheads show non-degraded NCS polypeptides. Values represent the mean ± standard deviation of 4 independent replicates.

Supplemental Table 1. Primers used to amplify candidate NCS genes

Primers	Description
PBR_rep_c6824-F (PBRNCS2)	AGTGTTTCAGAGAGTATGATGAGGA
PBR_rep_c6824-R (PBRNCS2)	CCCGCAATGACATCTAGCTT
PBRContig25754-F (PBRNCS4)	ACATCGACCGTGTAAAGCGA
PBRContig25754-R (PBRNCS4)	ACCTTAGAGTGGAACACGTCC
PBR_rep_c8842-F (PBRNCS3)	ACTTCCTGGTGTCTTCGTGAAA
PBR_rep_c8842-R (PBRNCS3)	ACTTGGCTTATGCTTTTAGACCTC
PBRContig45733-F (PBRNCS5)	AGTGAGTGAGTGTTTCAGAGAGT
PBRContig45733-R (PBRNCS5)	ACCTTAGAGTGGAACACGTCC
SCAContig30427-F (SCANCS1)	AGAGAGAGAAAATGAGGAAGGAACT
SCAContig30427-R (SCANCS1)	ACCGAACTTAGAATGGAACACCT
CMAContig5713-F (CMANCS2)	GTGTTTCAGAGAGAACGATGAGG
CMAContig5713-R (CMANCS2)	ACCTTAGAGTGGAACACCAGC
CMA_rep_c1557-F (CMANCS1)	CACGAGAAGCGATTGAAAGAGGTG
CMA_rep_c1557-R (CMANCS1)	TGGACCGGACGGTATACATGACCAT
SDI_rep_c489-F (SDINCS1)	GAGAAAATGAGGAAGGAAGTACGATA
SDI_rep_c489-R (SDINCS2)	CCGGTACTTAGAGTGGAACACC
ECAContig18893-F (ECANCS2)	AACCAAGAGAAGCGACTCAA
ECAContig18893-R (ECANCS2)	ACCTAAAGTAACTGAAACTATGCTG
ECA_rep_c12486-F (ECANCS1)	GCGAAAATACAGAGAGAAGTTTGTGA
ECA_rep_c12486-R (ECANCS1)	CCCCTGGAGGAAAAACAATTTGG
AME_rep_c2186-F (AMENCS1)	AGGGAGAGAAAATGAGGAAAGAAGT
AME_rep_c2186-R (AMENCS1)	CCTCAATGACATCTAACTTTTC
AMEcomp935-F (AMENCS2)	CAACCCTGCTATCTCCAAGTATGTT
AMEcomp935-R (AMENCS2)	AACAGGTAGCTAGGGCAGCTGTTTAT
TFLcomp2119-F (TFLNCS4)	AATGAGGAAGGAACTAACACATGAGA
TFLcomp2119-R (TFLNCS4)	GTGGCCTATCTCATCTTCACAGTACT
TFLcomp21856-F (TFLNCS5)	CAAGTTCATCACACTAACACAAGTAAG
TFLcomp21856-R (TFLNCS5)	CTTCGAATTCTAGGCAGAAGAATCCAC
TFL_rep_c456-F (TFLNCS2)	ACCAAAGGTCCTATTACCGAAGATGA
TFL_rep_c456-R (TFLNCS2)	CTCTAGACTACATCTTTCAAGCCCCA
TFL_rep_c2110-F (TFLNCS3)	GAATATATATGAAGATGGAAGCTAC
TFL_rep_c2110-R (TFLNCS3)	CCACTTAAGTACCTACAAACCCCAA
BTH_c15840-F (BTHNCS1)	GAATTGGTAAATGAGATGGTAGTGGC
BTH_c15840-R (BTHNCS2)	GTAGTATCTTGTTAACACGATTTGTC
MCAcomp5594-F (MCANCS1)	CAGTCCATCCCTTCTCAGTCAATTAA
MCAcomp5594-R (MCANCS1)	GTCAATCCCATAAGCCTAATAACCA
CCH_rep_c1173-F (CCHNCS1)	AGATGGAAGTGGCTACTTCAGCTGAT
CCH_rep_c1173-R (CCHNCS1)	TCTTGATTGAATTGGATCCCCTCAAT
CCH_rep_c7133-F (CCHNCS2)	GAGTGTGATAGTAGAAAGAAATGAG
CCH_rep_c7133-R (CCHNCS2)	CATTGCCTTCAATGACATCCTAGTC
CCH_rep_c1524-F (CCHNCS3)	CGAGAGACTAAAAGTAAGGAAAAG
CCH_rep_c1524-R (CCHNCS3)	ACCTTGACACCATTATTAGTACTTCC
CCH_rep_c156-F (CCHNCS4)	TAGCAAGAATGAGGAAGCATCTTG
CCH_rep_c156-R (CCHNCS4)	AGCTAGCTAGGTGCATCCATCATAAG
CCH_rep_c2691-F (CCHNCS5)	AATGAGGAAGGAACTCACAAATGAGT
CCH_rep_c2691-R (CCHNCS5)	TCTCCCAAGCAAACAAAGCATTG
NDO_rep_c12880-F (NDONCS1)	TCTAGTTTGCATTATCAAGGAGAGGA
NDO_rep_c12880-R (NDONCS1)	ACATAGCGATGATGATTATATTTCGA
NDO_rep_c17645-F (NDONCS2)	CTTGAAATGGTATTTCCTCCAGGA
NDO_rep_c17645-R (NDONCS2)	AGTCGCATACATCCACATTTTGTTTC
NDO_rep_c11505-F (NDONCS3)	AATGAGGAGTGGAATTGTTTTCCTG
NDO_rep_c11505-R (NDONCS3)	GATTACACTACACGATGCAACTTTG
NDO_rep_c14985-F (NDONCS4)	GTAAATGAGATGGAAGTGGCTGCGT
NDO_rep_c14985-R (NDONCS4)	AGCATACATCTTGTTAATGACGCTTC
CTR_c5246-1-F (CTRNCS1)	GCCTGCATCAGCTTAGAACAC

CTR_c5246-1-R (CTRNCS1)	TGGCAGTCCACTTCCAATTCA
HCA_rep_c19-F (HCANCS1)	CGATCTTGCATCTGTAAACATTTCA
HCA_rep_c19-R (HCANCS1)	GCGTACGTACTCAAACAAGTATTTCT
NCA_rep_c28-F (NCANCS1)	TAAATAAGATGGTTCAGTTCAGCAGA
NCA_rep_c28-R (NCANCS1)	GAGCAGAAGTTGTGTTCCTCAGATTG
NCA_rep_c877-F (NCANCS2)	TGAGAGGAAGCAAGCACAAGG
NCA_rep_c877-R (NCANCS2)	CGGTCTTGTACCTGGGATGAT
XSIcomp133-F (XSINCS1)	GCAAGAAGGTTTCCTTAGTGCAA
XSIcomp133-R (XSINCS1)	TCAGTAGCTGCTTTGAACCAT
PSO_rep_c3975-F (PSONCS3)	TCGAGTGTTTCAGAGAGAACGA
PSO_rep_c3975-R (PSONCS3)	ACCCATTTTTCAAACATCGCCA

Supplemental Table 2. Primers designed to include *Hind*III, *Bam*HI or *Xho*I restriction sites and used to obtain the coding region of candidate NCS genes cloned into pET29b for expression in *Escherichia coli.*

Primers	Description
CCHNCS1-*Hind*III	CCAAGCTTATGGAAGTGGCTACTTCA
CCHNCS1-*Xho*I	GCTCGAGTATCGAAACACCGCCGAT
CCHNCS2-*Hind*III	CCAAGCTTATGAGGAAGGAATTAAGA
CCHNCS2-*Xho*I	GCTCGAGGTCTTCGAAAACTCCA
CCHNCS5-*Hind*III	CCAAGCTTATGAGGAAGGAACTCACA
CCHNCS5-*Xho*I	GCTCGAGACCCAAACAATTGAAAGG
CMANCS1-*Bam*HI	CGGGATCCTATGATTGAAGGAGGGTA
CMANCS1-*Xho*I	GCTCGAGGAGTGGAACACCCCCAAT
ECANCS1-*Hind*III	CCAAGCTTATGATCGGAGGATTCTTA
ECANCS1-*Xho*I	GCTCGAGATGACTTCTAACTTTTCGA
NDONCS3-*Hind*III	CCAAGCTTATGAGGAGTGGAATTGTT
NDONCS3-*Xho*I	GCTCGAGTATTTCGATAAACCCCTT
PBRNCS3-*Hind*III	CCAAGCTTATGGATATCATAGAAGGG
PBRNCS3-*Xho*I	GCTCGAGTGCTTTTAGACCTCCAAT
PBRNCS4-*Hind*III	CCAAGCTTATGATCGAAGGAGGGTAT
PBRNCS4-*Xho*I	GCTCGAGGAGTGGAACACGTCCAAT
PBRNCS5-*Hind*III	CCAAGCTTATGATGAGGAAAGTAATC
PBRNCS5-*Xho*I	GCTCGAGGAGTGGAACACGTCCA
SCANCS1-*Hind*III	CCAAGCTTATGAGGAAGGAACTGACA
SCANCS1-*Xho*I	GCTCGAGGAATGGAACACCTCCAAT
SDINCS1-*Bam*HI	CGGATCCTATGAGGAAGGAAGTACG
SDINCS1-*Xho*I	GCTCGAGGAGTGGAACACCTC
TFLNCS1-*Hind*III	CCAAGCTTATGAAGATGGAAGTTGTA
TFLNCS1-*Xho*I	CCAAGCTTATGAGGATGGAAGTTGTT
XSINCS1-*Hind*III	GCTCGAGCTCTGATCTCTTGTATTTCT
XSINCS1-*Xho*I	CCAAGCTTATGAGGAAAGTAATCAAAT
PSONCS3-*Hind*III	CCAAGCTTATGAGGAAAGTAATCAAAT
PSONCS3-*Xho*I	GCTCGAGGCTTAGCCATTTTACCA

Supplemental Table 3. Primers used to obtain truncated versions of candidate NCS genes.

Primers	Description
PBRNCS3-25-*Hin*dIII	CCAAGCTTAGTTACAAGGAGAGATTTG
PBRNCS3-*Xho*I	GCTCGAGTGCTTTTAGACCTCCAAT
ECANCS1-25-*Hin*dIII	CCAAGCTTTCATGTATTATCAAATCAAC
ECANCS1-*Xho*I	GCTCGAGATGACTTCTAACTTTTCGA
CMANCS1-25-*Bam*HI	CGGGATCCTAATTCATGCGTTATTGCAT
CMANCS1-*Bam*HI	CGGGATCCTATGATTGAAGGAGGGTA
CCHNCS2-25-*Hin*dIII	CCAAGCTTGATATCCCAAGACTTC
CCHNCS2-*Xho*I	GCTCGAGGTCTTCGAAAACTCCA
TFLNCS2-25-*Hin*dIII	CCAAGCTTAGGCCATTTCTTAACCG
TFLNCS1-*Xho*I	CCAAGCTTATGAGGATGGAAGTTGTT
PBRNCS4-25-*Hin*dIII	CCAAGCTTAGCTCATGTGTTATTGAATC
PBRNCS4-*Xho*I	GCTCGAGGAGTGGAACACGTCCAAT
CCHNCS5-25-*Hin*dIII	CCAAGCTTGATCTCCCAAAAATCATA
CCHNCS5-*Xho*I	GCTCGAGACCCAAACAATTGAAAGG
XSINCS1-25-*Hin*dIII	CCAAGCTTGGGCGTCCTCTCCT
XSINCS1-*Xho*I	CCAAGCTTATGAGGAAAGTAATCAAAT

Supplemental Table 4. Primers used to amplify NCS gene candidates for insertion into pESC-leu2d for expression in yeast.

Primers	Description
SCANCS1-*Not*I	TAAAGGGCGGCCGCAAAAATGAGGAAGGAACTGACACACG
SCANCS1-*Bgl*II	AGACTGAGATCTTCAATGGTGATGGTGATGATG GAATGGAACACCTCCAATCAATAAC
NDONCS3-*Not*I	TCAAGTGCGGCCGCAAAAATGAGGAGTGGAATTGTTTTCC
NDONCS3-*Bgl*II	GTACCTAGATCTTCAATGGTGATGGTGATGATG TATTTCGATAAACCCCTTGTG
CCHNCS2-*Not*I	TAAAGGGCGGCCGCAAAAATGAGGAAGGAATTAAGACATG
CCHNCS2-*Spe*I	CGCGATACTAGTTCAATGGTGATGGTGATGATGGTCTTCG AAAACTCCAGGAA
PBRNCS5-*Not*I	TTAAGGGCGGCCGCAAAAATGATGAGGAAAGTAATCAAATACG
PBRNCS5-*Bgl*II	GTACTCAGATCTTCAATGGTGATGGTGATGATG GAGTGGAACACGTCCAATC
PSONCS3-*Spe*I	ATGAGGAAAGTAATCAAATACGATATGGAGGTAGC
PSONCS3-*Pac*I	AGCGTTAATTAATCAATGGTGATGGTGATGATGAGATTGTTGCTTCATAGCGT A
XSINCS1-25-*Not*I	TAAAGGGCGGCCGCAAAAATGAGGCCATTTCTTAACCGC
XSINCS1-25-*Bgl*II	AGACTGAGATCTTCAATGGTGATGGTGATGATGCTCTGATCTCT TGTATTTCTTCTCAAGAAC
PsNCS2-*Not*I	GAAAGGGCGGCCGCAAAAATGTCTAAATTAATCACAACTGAACC
PsNCS2-*Sac*I	GTTGCTGGTGTTCCACTCCATCATCACCATCACCATTGA GAGCTCGAGCG
TfNCSD19-*Not*I	TAAAGGGCGGCCGCAAAAATGCAGAAACTGATTCTGACAGGTAG
TfNCSD19-*Bgl*II	AGACTGAGATCTTCAATGGTGATGGTGATGATGGACTGTTATTATTGCGGCC
PSONCS3D306-641-*Spe*I	GGCCGCACTAGTAAAAATGAGGAAAGTAATCAAATACGATATGGAGGTAGC
PSONCS3D306-641-*Pac*I	CTTAATTAATCAATGGTGATGGTGATGATGGATTAATTTAGACATGGCATCAA CATACTC

Assembled nucleotide sequences

>PBRSC1NG_011_C02
ATGATGAGGAAAGTAATCAAATACGATATGGAGGTAGCTACCTCAGCTGATTCAGTATGGGCAGTTTACAG
TTCACCGGATATTCCAAGGCTTCTCAGGGATGTTCTACTTCCCGGCGTCTTCGAGAAATTAGACGTCATTG
AAGGGAATGGCGGCGTCGGTACAGTTCTTGACATTGCTTTTCCTCCAGGTGCGGTTCCTCGAAGTTACAAA
GAGAAATTCGTCAACATCGACCGTGTAAAGCGATTGAAAGAAGTGATCATGATTGAAGGAGGATACCTGGA
CATGGGATGCACATTTTACTTGGACAGGATCCATGTCGTGGAGAAAACCCCAAACTCATGTGTCATTGAAT
CCTCTATTATCTACGAAGTTAAAGAAGAGTTTGCTGATAAAATGGCTAAGCTAATCACAACGGAACCATTG
CAGTCGATGGCAGAAGTCATCTCTGGTTATGTTCTTAAGAAACGACTCCAAGTATTTGGATTCGAGATTAA
GCCAAACTTAAGATTCAATCTTTTGCTATGTTTGATTATCTGCTTGGTTATAGCTGGAGGTATGTTGATTG
GACGTGTTCCATAA

>TFLNCS
ATGATGAAGATGGAAGTTGTATTTGTTTTCTTAATGTTGTTAGGAACAATAAATTGCCAGAAACTGATTCT
GACAGGTAGGCCGTTTCTGCACCACCAGGGCATAATAAACCAGGTGTCTACAGTCACAAAAGTGATTCATC
ATGAGTTGGAAGTTGCTGCTTCAGCTGATGATATATGGACTGTTTATAGCTGGCCTGGCTTGGCCAAGCAT
CTTCCTGACTTGCTCCCTGGCGCTTTTGAAAAGCTAGAAATCATTGGTGATGGAGGTGTTGGTACCATCCT
AGACATGACATTTGTACCAGGTGAATTTCCTCATGAATACAAGGAGAAGTTTATATTAGTCGATAATGAGC
ATCGTTTAAAGAAGGTGCAAATGATTGAGGGAGGTTATCTGGACTTGGGAGTAACATACTACATGGACACA
ATCCATGTTGTTCCAACTGGTAAAGATTCATGTGTTATTAAATCCTCAACTGAGTACCATGTGAAACCTGA
GTTTGTCAAAATCGTTGAACCACTTATCACCACCGGTCCATTAGCTGCCATGGCAGACGCCATCTCAAAAC
TTGTTCTAGAACACAAATCCAAAAGCAACTCAGATGAAATTGAGGCCGCAATAATAACAGTCTGA

>PSONCS1
ATGTCTAAGTTGATCACGACGGAACCATTGAAGTCGATGGCAGAAGTTATCTCTAATTACGCTATGAAGCA
ACAATCTGTTTCTGAGAGAAACATTCCTAAGAAGCAATCTCTACTGAGGAAGGAAATTACTTATGAAACGG
AGGTGCAAACTTCTGCTGATTCAATTTGGAACGTCTACAGTTCTCCTGACATCCCTCGACTACTTAGAGAT
GTTCTGCTTCCTGGTGTTTTTGAAAAGCTAGATGTCATAGCAGGCAATGGTGGAGTTGGTACGGTACTGGA
TATTGCCTTCCCTCTAGGTGCAGTGCCACGGAGGTACAAGGAGAAATTTGTGAAGATCAACCATGAGAAGC
GATTGAAAGAAGTGGTGATGATCGAAGGAGGATACTTAGACATGGGGTGCACATTTTACATGGACAGGATC
CATATCTTTGAGAAAACCCCAAACTCATGTGTTATCGAATCCTCGATCATTTACGAAGTTAAAGAAGAGTA
TGCTGGTAAAATGGCTAAGCTAATCACAACTGAACCATTGGAATCCATGGCAGAAGTCATCTCTGGTTATG
TTCTTAAGAAACGACTCCAAGTATTCGGATTCGAGATTAAGCCAAAATTAAGATTCAATCTTTTGCTATGT
TTGATTATCTGTCTGGTTATAGCTGGAGGTATGTTTGTTGCTGGTGTTCCACTCTAA

>PSONCS2
ATGTCTAAATTAATCACAACTGAACCATTGAAGTCGATGGCGGAAGTCATCTCTAATTACGTTATCCAGAG
AGAATCATTTTCCGCAAGAAACATTCTCAACAAAAATTCTTTGGTGAAGAAGGAGATTCGGTATGACCTGG
AGGTTCCAACCTCAGCTGATTCTATCTGGTCAGTTTACAGCTGCCCGGATATTCCTCGGCTTCTTAGAGAT
GTTTTACTTCCCGGTGTTTTCCAGAAATTGGATGTTATCGAAGGGAATGGTGGTGTTGGTACAGTTCTTGA
TATCGTTTTTCCTCCAGGTGCGGTACCTCGTAGTTACAAGGAGAAATTTGTGAACATCAACCACGAAAAGC
GATTAAAAGAAGTGATTATGATCGAAGGAGGATATTTAGACATGGGATGCACATTTTACATGGACAGGATC
CATATCTTTGAGAAAACCCCAAACTCATGTGTTATCGAATCCTCGATCATTTACGAAGTTAAAGAAGAGTA
TGCTGGTAAAATGGCTAAGCTAATCACAACTGAACCATTGGAATCCATGGCAGAAGTCATCTCTGGTTATG
TTCTTAAGAAACGACTCCAAGTATTCGGATTCGAGATTAAGCCAAAATTAAGATTCAATCTTTTGCTATGT
TTGATTATCTGTCTGGTTATAGCTGGAGGTATGTTTGTTGCTGGTGTTCCACTCTAA

>CJAPR10A
ATGAGGATGGAAGTTGTTCTAGTTGTTTTCTTGATGTTCATAGGTACGATAAATTGTGAAAGATTGATATT
CAATGGACGACCGCTACTCCATCGCGTAACAAAAGAGGAGACTGTAATGCTTTATCATGAGCTGGAAGTAG
CTGCTTCAGCCGATGAAGTGTGGAGTGTCGAAGGTTCGCCTGAGTTGGGCTTGCATTTGCCTGACTTGCTC
CCTGCTGGTATATTTGCAAAGTTTGAAATTACTGGTGATGGAGGTGAAGGTTCGATCCTGGACATGACATT
CCCCCCAGGTCAGTTTCCACATCATTACAGGGAGAAGTTCGTGTTCTTCGATCACAAGAATCGTTACAAGT
TAGTAGAACAGATCGATGGTGATTTTTTCGATCTAGGTGTTACATACTATATGGATACAATCCGAGTTGTT
GCGACAGGCCCTGATTCATGTGTCATCAAGTCTACTACTGAATACCATGTGAAACCTGAGTTTGCCAAAAT
CGTCAAACCACTTATTGACACTGTTCCACTAGCTATCATGTCTGAAGCGATTGCAAAGGTTGTTCTAGAGA
ACAAACACAAGAGTTCAGAGTAA

>PBRNCS2
ATGATGAGGAAAGTAATCAAATACGATATGGAGGTAGCTACCTCAGCTGATTCAGTCTGGGCAGTTTACAG
TTCACCGGATATTCCTAGACTTCTCAGGGATGTTCTACTTCCCGGCGTCTTTGAGAAATTAGACGTCATTG
AAGGGAATGGCGGCGTTGGTACTGTTCTTGACATTGCTTTCCCTCCAGGTGCGGTTCCTCGAAGTTACAAA
GAGAAATTCGTCAACATCGACCGTGTAAAGCGATTGAAAGAAGTGATCATGATTGAAGGAGGATACCTGGA
CATGGGATGCACATTTTACTTGGACAGGATCCATGTCGTGGAGAAAACTCCGAGCTCATGTGTTATTGAAT
CGTCTATTGTTTATGAAGTGGAAGAAGAGTACGCTGATGCGATGTCAAAATTGATCACYACTGAACCATTG
AAGTCGATGGCGGAAGTGATYTCTAATTACGTTATTCAGAAAGAATCAGTTTCCGCAAGAAACATTTTCAA
CAGGCAATCTGTAGTGAAGAAGGAGATTCATTACGACCTGGAGGTACCAACCTCAGCTGATTCGATCTGGG
CAGTTTACAGCAATCCCGATATCCCTCGGCTACTTAGAGATGTTCTGCTTCCTGGCGTTTTCGAGAAATTG
GATGTCATTGAAGGGAATGGTGGTGTTGGCACTGTTCTTGATATTGTTTTCCCTCCAGGTGCGGTGCCTCG
TTGTTACAAGGAGAAGTTTGTGACCATGGACCACCAAAAGCGACTAAAAGAAGTGATTATGATCGAAGGAG
GTTACTTAGACATGGGATGCACATCTTACCTGGACAGGATCCATGTTATAGAAAAAACCTCTAAATCTTGC
ATCATTAAATCTTCTGTTGTCTACGAAGTGAAGCAAGAGTGTGCTGAAGCAATATCTAAGTTGATCACGAC
GGAGCCATTGAAGTCGATGGCAGAAGTCATCTCAAATTATGTTCTTAAGAAACAATCTGTTTCCGACACAA
ACAATATTGCTAAGAAGCAATCTGTGTTGAGGAAAGAAATTACTTATGAAACGGAGGTGCAAACCTCAGCT
GATTCGATTTGGAACGTCTACAGTTCTCCTGACATTCCCCCGACTACTTAG

>PBRNCS3
ATGGATATCATAGAAGGGGATGGTGGAGTTGGTACTGTTCTTGATGTTGTTTTCCAACCTGGTGCGGTGCC
TCAAAGTTACAAGGAGAGATTTGAGACCGTGGACCACGAGAAGCGAATACTGGAAGTGAGAATTATCCAAG
GAGGATACTTAGAAATGGGTTGCACATCTTACCTGAATAGGATGCATGTTATTGAAATAACCTCTAAATCT
TGTGTTATTAAATCTTCGGTTATCTACGACGTGAAAGAAGAGTGTGCTGATGCAATGTCTAAGTTAATCAC
AACCATACAGTTGGAGTCAATGGCCAAAGTGGTCGCTGATTATGTTCTTAAGAAACAATCTGCTTCTGACA
CAAGCATTCCTAAGAAGCAGTCTCTAATGAGGAAAGAAATTACACATGAGATGGAGGTGCAAACCTCAGCT
GATTCGATTTGGGACATCTACAGTTCTCCTGACATCCCTCGACTACTTAGAGATGTCCTGCTTCCTGGTGC
TTTCGAAAAGCTACATGTCATTCAAGGCAATGGTGGGGTTGGTACTGTACTGGACATCGCTCTCCCTCTAG
GTGCAGTGCCACGAAATTACAAGGAGAAATTTGTGAAGATCAACCACGAGAAGCGACTAAAAGAAGCAGTT
ATGATTGAAGGGGGATACGCAGACATGGGGTGCACATTTTACATGCACAGGATCCATGTCCTAGAGAAAAC
ACCAAAGTCGTGTGTCATTGAATCCTCCATCGTTTACGAAGTGAAAGAAGAGTATGCTGATAAAATGTCAA
AGCTAATCACAACAGAACCATTGCAGTCCATGGCAGAAGCCATATCTAGTTATGTTCTTAAGAAACAGTTC
CAAGTATTTGGATTAGAGGTTAAACCGAAATTAGTATTAAGTCTATTTCTATGTTTGATCATCTTTTTGGC
TATAGTTGGTGGTTTTTTGATTGGAGGTCTAAAAGCATAA

>PBRNCS4
ATGATTGAAGGAGGATACCTGGACATGGGATGCACGTTTTACTTGGACAGGATCCATGTCGTGGAGAAAAC
TCCGAGCTCATGTGTTATTGAATCGTCTATTGTTTATGAAGTGAAGCAAGAGTGTGCTGAAGCAATATCTA
AGTTGATCACGACGGAGCCATTGAAGTCGATGGCAGAAGTCATCGCTAATTACGTTCTTAAGAAACAATCT
GTTTCTGACACAAACATTCCTAAGAAGCAATCTGTGTTGAGGAAAGAAATTACTTATGAAACGGAGGTGCA
AACCTCAGTTGATTCGATTTGGAACGTCTACAGTTCTCCAGACATCCCTCGACTACTTAGAGATGTTCTGC
TTCCTGGTGTTTTTGAGAAGCTAGATGTCATTGCAGGCAATGGTGGCGTTGGGACTGTACTGGACATTGCT
TTCCCTCTAGGTGCAGTGCCGCGGAGGTATAAGGAGAGATTTGTGAAGATCAATCATGAGAAGCGGTTGAA
AGAAGTGGTTATGATCGAAGGAGGGTACTTGGACATGGGCTGCACATTTTACATGGACAGGATTCATGTCT
TTGACAAAACCCCAAACTCATGTGTCATTGAATCCTCTATTATCTACGAAGTTAAAGAAGAGTATGCTGAT
AAAATGGCTAAGCTAATCACAACGGAACCATTGCAGTCGATGGCAGAAGTCATCTCTGGTTATGTTCTTAA
GAAACGACTCCAAGTATTTGGATTCGAGATTAAGCCAAACTTAAGATTCAATCTTTTGCTATGTTTGATTA
TCTGCTTGGTTATAGCTGGAGGTATGTTGATTGGACGTGTTCCACTCTAA

>PBRNCS5
ATGATGAGGAAAGTAATCAAATACGATATGGAGGTAGCTACCTCAGCTGATTCAGTCTGGGCAGTTTACAG
TTCACCGGATATTCCAAGGCTTCTCAGGGATGTTCTACTTCCCGGCGTCTTCGAGAAATTAGATGTCATTG
AAGGGAATGGCGGCGTTGGTACTGTTCTTGACATTGCTTTCCCTCCAGGTGCGGTTCCTCGAAGTTACAAA
GAGAAATTCGTCAACATCGACCGTGTAAAGCGATTGAAAGAAGTGATCATGATTGAAGGAGGATACCTGGA
CATGGGATGCACATTTTACTTGGACAGGATCCATGTCGTGGAGAAAACTCCGAGCTCATGTGTTATTGAAT
CGTCTATTGTTTATGAAGTGGAAGAAGAGTACGCTGATGCCATGTCAAAATTGATCACCACTGAACCATTG
AAGTCGATGGCGGAAGTGATTTCTAATTACGTTATCCAGAAAGAATCAGTTTCCGCAAGAAACATTTTCAA
CAGGCAATCTGTAGTGAAGAAGGAGATTCGATACGACCTGGAGGTACCAACCTCAGCTGATTCTATCTGGG

```
CAGTTTACAGCAATCCCGATATCCCTCGGCTACTTAGAGATGTTCTGCTTCCTGGCGTTTTCGAGAAATTG
GATGTCATTGAAGGGAATGGTGGTGTTGGGACTGTTCTTGATATCGTTTTTCCTCCAGGTGCGGTGCCTCG
TCGTTACAAGGAGAAATTTGTGAACATCAACCACGAGAAGCGATTAAAAGAAGTGATTATGATCGAAGGAG
GGTACTTAGACATGGGATGCACATTTTACCTGGACAGGATCCATGTTGTAGAAAAAACCTCTAAATCTTGC
ATCATTAAATCTTCTATTGTTTACGAAGTGAAGCAAGAGTGCGCTGAAGCAATATCTAAGTTGATCACGAC
GGAGCCGTTGAAGTCGATGGCACAAGTCATCGCTAATTATGTTCTTAAGAAACAATCTGTTTCTGACACAA
ACATTCCTAAGAAGCAATCTGTGTTGAGGAAAGAAATTACTTATGAAACGGAGGTGCAAACCTCAGTTGAT
TCGATTTGGAACGTCTACAGTTCTCCAGACATCCCTCGACTACTTAGAGATGTTCTGCTTCCGGGTGTTTT
TGAAAAGCTAGATGTCATTGCAGGCAATGGTGGCGTTGGGACTGTACTGGACATTGCTTTCCCTCTAGGTG
CAGTGCCGCGGAGGTACAAGGAAAAATTTGTGAAGATCAACCATGAGAAACGATTGAAAGAAGTGATTATG
ATCGAAGGAGGATACTTAGACATGGGGTGCACATTTTACATGGACAGGATTCATGTACTTGAGAAAACCCC
AAACTCATGTGTCATTGAATCCTCTATTATCTACGAAGTTAAAGAAGAGTTTGCTGATAAAATGGCTAAGC
TAATCACAACGGAACCATTGCAGTCGATGGCAGAAGTCATCTCTGCTTATGTTCTTAGGAAACGATTCGAA
GTATTTGGACTAGAGATTAAGCAAAAATTAAGATACAATCTTTTGCTATGTTTGATTATCTGCTTGGTTAT
AGCTGGAGGTATGTTGATTGGACGTGTTCCACTCTAA

>SCANCS1
ATGAGGAAGGAACTGACACACGAGATGGAGGTGCCTGCCTCAGCCGATGCTATTTGGGCAGTCTACAGTTC
ACATGATATTCCAAGGCTGCTCAAAGAAGTTTTGCTTCCTGGTGTTTTTGAAAAGCTAGATGTCATTGCAG
GTGATGGTGGTGTTGGTACTGTTCTCGACATTGCTTTCCCTCCAGGGGCGGTACCGCGTCGTTACAAGGAG
AAATTCGTGAAGATCAATCACGAGAAGCGATTGAAGGAAGTGGAGATGATCGAAGGAGGGTATTTGGATAT
GGGGTGTACATTTTATATGGACAGGATTCATGTCGTAGAGAAAGGTCCTAATTCATGCGTTATCGAATCGG
CGATTATTTACGTAGTGAAGGACGAATGCGCCGATGTCGTCGTTCCTCTAATTACGACTGAACCACTGGCT
AGCATGGCGGAGGTCATCTCAAATTACGTTCTAAGGAAACAAATCCGATTGTTTGGATACGTAATTAAACC
AAAATTAGGGTTAAGTATTTTGCTCTCCTTGATTCTCTGCCTAGTTATACTAGGAGTGTTATTGATTGGAG
GTGTTCCATTCTAA

>CMANCS1
ATGATTGAAGGAGGGTATTTGGATATGGGATGTACATTTTACATGGACAGAATCCATGTTGTAAAGAAAGG
TCCCAATTCATGCGTTATTGCATCGGCTATTATCTACGAGGTGAAGGAGGAATTTGTCGACGTCGTCGTTC
CTCTAATCACGACCGAACCATTGGCTAGCATGGCAGAAGTCATCTCAAATTACGTTCTTAAGAAACAACGT
CGTGTAAGGAAGGAACTAACATATGAGATGGAGGTGCCTACCTCAGCTGATTCAATTTGGGCAGTCTACAG
TTCACATGATATTCCAAGGCTCCTCAAAGAAGTTCTGCTCCCTGGTGTCTTTGAAAAGCTTGATGTCATTG
AAGGTGATGGTGGTGTTGGTACTGTTCTTGACATTGCTTTCCCACCAGGGGCGGTACCACGCACTTACAAG
GAGAAATTCGTGAAGATCAATCACGAGAAGCGATTGAAAGAGGTGGTGATGATTGAAGGAGGGTATTTGGA
TATGGGATGTACATTTTACATGGACAGAATCCATGTGCTAGAGAAAAGTCCTAACTCGTGCGTTATTGAAT
CTTCTATTATCTACGAGGTGAAGGAGGAATTTGCCGATGTCGTGGGTCCTCTAATCACGACCGAGCCACTA
GCTAGCATGTCAGAGGTCATCTCAAATTACGTTCTAAAGAAACAAATCCGCATGTTTGGTTATGTAATTAA
ACCAAAACTTGGTTTAAGTCTTTTGCTCTGCTTCATTCTCTGCCTCGTTTTACTTGGAGTTTTATTGATTG
GGGGTGTTCCACTCTAA

>SDINCS1
ATGAGGAAGGAAGTACGATATGAGATGGAGGTACCTACCTCAGCTGATTCAATTTGGGCAGTTTACAGTTC
ACATGATATTCCAAGGCTCCTCAAAGAAGTTCTTCTCCCTGGTGTCTTTGAAAAGCTTGATGTCATTGAAG
GTGATGGTGGTGTTGGTACTGTTCTTGACATTGCTTTCCCACCAGGGGCGGTACCACGCACTTACAAGGAG
AAATTTGTGACAATCAATCATGAGAAGCGATTGAAAGAGGTGATTATGATTGAAGGAGGGTATTTGGATAT
GGGATGTACATTTTACATGGACAGAATCCATGTCCTAGAGAAAGGTCCCAAATCATGCATTATTGCATCGG
CTATTATCTATGAGGTGAAAGAAGAATTCGCCGATGTCGTCGTTCCTCTAATCACGACTGAACCATTGGCT
AGCATGGCAGAGGTCATCTCCAATTACGTTCTTAAGAAACAACGCCGTGTAAGGAAGGAATTAACATATGA
GATGGAGGTTCCTACCTCAGCTGATTCAATTTGGGCAGTTTACAGTTCACATGATATTCCAAGGCTCCTCA
AAGAAGTCCTTCTCCCTGGTGTGTTTGAAAAGCTTGATGTCATTGAGGGTGATGGTGGTGTTGGTACTGTT
CTTGACATTGCTTTCCCTCCAGGTGCGGTACCACGCACTTACAAGGAGAAATTCGTGAAGATCAATCACGA
GAAGCGATTGAAAGAGGTGGTGATGATTGAAGGAGGGTATTTGGATATGGGATGTACATTTTACATGGACA
GAATCCATGTCCTAGAGAAAGGTCCTAATTCTTGCGTTATTGAATCTGCTATTATCTACGAGGTGAAAGAA
GAATTTGCTGATGTCGTCGTTCCACTAATCACGACCGAACCACTAGCTAGCATGGCAGAGGTCATCTCAAA
TTACGTTCTAAAGAAACAAATCCATGTGTTTGGTTATGTAATTAAACCAAAACTTGGATTAAGTCTTTTGC
TCTGCTTCATTCTCTGCCTCGTTTTACTTGGAGTTTTATTGATTGGAGGTGTTCCACTCTAA
```

>ECANCS1
ATGATCGGAGGATTCTTAGACATGGGATGTACATTTTACATGGACAGGATTCATGTCGTAGCGAAAGGTCC
TAATTCATGTATTATCAAATCGACTCTTATCTACGAAGTGAAAGAGGAATATGCCGATGCCATGGCTTCTC
TAATCACTGTAGAACCACTAGCTAGCATGGCAGAAGTTGTTGCAAATTACGTTCTTCATCAACAAGTCCGG
GTGTTAGGATCCGTGAAGAGGAAGGAACTTACGCATGAGTTGGAAGTTGCTGCACCAGCTGATGCTATTTG
GGGTGTGTATAGCTCACCTGATATTCCGAGGCTTCTGAGGGATGTTTTGCTTCCGGGTGTTTTTGAAAAGT
TAGAAGTTATACAAGGAAATGGAGGTGTTGGTACTGTTCTTGAGATTGTTTTCCATCCAGGTGCAATTCCG
CGTAGGTACAAGGAGAAGTTTGTGACGATAAATCACAAGAAGCGACTGAAAGAGGTGGTCATGATTGGAGG
GTATCTAGACATGGGGTGTACACTTTATATGGACAGGATTCATGTAGTATCCAAAGGTCCTAATTCATGTG
TTATCAAATCGACACTCATTTATGAAGTTAAAGCAGAATCAGCAGATGCGATGGCTTCTACAATCACCATC
GACCCACTCGCTAGCATGGCACAGGTCATCTCAAATTATGTTCTCAAGAATCAAATGCAAGTCTTAGGATC
TGTTAAGAGAAGGGAATTAACACATGAGTTAGAGGTAGCTGCCTCAGCTGACGCTATTTGGGGAGTTTATG
GATCAAAAAGATATTCCAAGGCTTCTCAGGGATGTTTTGCTTCCTGGTGTTTTCGAAAAGTTAGAAGTCAT
TGA

>ECANCS2
ATGATCGGAGGGTACCTAGACATGGGGTGTACTCTTTATATGGACAGGATTCATGTGGTTGAGAAAGGTCC
AAACTCATGTGTTATAAAATCGACACTTATTTATGAAGTGAAAGCAGAATATGCGGATGCCATGGCTTCTC
TAATCACAGTCGACCCACTCGCTAGCATGGCACAAGTGATCTCAAATTATGTTCTCAAGAATCAAGGCCAA
GTCTTAGGATCTATAAAGAGAAGGGAACTAAAACATGAATTGGAGGTAGCTGTCTCTGCTGATGCTATTTG
GGGAGTTATTGGTTCAAAAGATATCCCAAGGCTTCTTAGAGATGTTTTGCTTCCTGGTGTTTTTGAGAAGT
TAGATGTCATTGAAGGTGATGGAGGCGTGGGTACTGTTCTTGAAATTGTTTTCCCTCCAGGAGCTGTTCCG
CGAAAATACAGAGAGAAGTTTGTGAAGGTCGATCATGAGAAACGACTGAAAGAGGTGATCATGATCGGAGG
GTACTTAGACATGGGATGTACATTTTACATGGACAGGATTCATGTCGTAGCGAAAGGTCCTAATTCATGTA
TTATCAAATCGACTCTTATCTACGAAGTGAAAGAGGAATATGCCGATGCAATGGCTTCTCTAATCACCGTA
GAACCACTAGCTAGCATGGCAGAAGTTGTTGCAAATTACGTTCTCCATCAACAAGTCCGGGTGTTAGGATC
CGTGAAGAGGAAGGAACTTACACATGAGTTGGAAGTAGCTGCATCAGCTGATGCTATTTGGGGTGTGTATA
GCTCACCTGATATTCCGAGGCTTCTGAGGGATGTTTTGCTTCCGGGTGTTTTTGAAAAGTTAGAAGTTATA
CAAGGAAATGGAGGTGTTGGTACTGTTCTTGAGATTGTTTTCCATCCAGGTGCAATTCCGCGTAGGTACAA
GGAGAAGTTTGTGACGATAAATCACAAGAAGCGACTGAAAGAGGTGGTCATGATTGGAGGGTATCTAGACA
TGGGGTGTACACTTTATATGGACAGGATTCATGTAGTATCCAAAGGTCCTAATTCATGTGTTATCAAATCG
ACACTCATTTATGAAGTTAAAGCAGAATCAGCAGATGCCATGGCTTCTACAATCACCATCGACCCACTCGC
TAGCATGGCACAGGTCATCTCAAATTACGTTCTCAAGAATCAAATGCAAGTCTTAGGATCTGTTAAGAGAA
GGGAATTAACACATGAGTTAGAGGTAGCTGCCTCAGCTGACGCTATTTGGGGAGTTTATGGATCAAAAGAT
ATTCCAAGGCTTCTCAGGGATGTTTTGCTTCCTGGTGTTTTCGAAAAGTTAGAAGTCATTGAAGGCGATGG
AGGTGTTGGTACTGTTCTCCAAATTGTTTTTCCTCCAGGGGCAATTCCACGGAGGTACAAAGAGAAATTTG
TGAAAGTCGATCAGAAGCTGCGACTAAAAGAAGTGATCATGATCGGAGGATACTTGGACATGGGTTGTACG
TTTTATATGGACAGGATTCATGTAGTACCAAAAGGTCTTAATTCATGTGTTATCAAATCAACACTTATTTA
TGAAGTGAAAGATGAATATGCTGATGCCATGTCCTCTCTTATCACTGTCGAGCCACTCGCTAGCATGGCTG
AAGTTGTTTCAAATTATGTTCTGAATAAAAAGAAACTAATGATAACAAGGAAAGAACTAACACATGAGTTG
GAGGTGGCAGCCTCAGCCGATGCTATTTGGAGTGTCTATAGCTCACCTGATATTCCAAGGCTTCTCAGAGA
TGTTTTGCTTCCTGGCGTTTTTGAAAAGTTAGAAGTTGTTCAAGGAAATGGTGGCGTTGGTACTGTTCTCG
AAATTGTTTTCCCTAAAGGATCTGTTCCACGGAGGTACAAAGAGAAGTTTGTGAAAATCAACGACGAGAAA
AAGCTGAAAGAGGTAATCATGATCGAAGGAGGATACTTGGACTTGGGATGTACATTTTACATGGACAAAAT
TCATGTCTTACCTAAAGGGCCTAATTCATGTGTTATCGAATCGTCACTTATTTACGAAGTTAAAGAAGAAA
ATGCAAAAGCCATGGCTTCTCTGATTACTGTTGAACCACTTGCTAGCATGGCTGAAGTCGTTGCAAATTAC
GTTCTTAAGAAGCAAATCCGTGTGTTAGGATACGTCGTTAAGCCAAGAGTTGGATATAGTGTTTTGGTTGG
GCTTTTACTCTGCTTGGTTCTACTTGGAGTTTTATTGCTTTCAGGTGTCAACATCTAA

>AMENCS1
ATGAGGAAAGAAGTTGTATATGAATTGGAAGTACCAACTTCAGCTGATTCAATATGGGCAGTTTACAGTTC
ACCCAACATTCCTACACTTCTAAGAGATGTTCTACTTCCTGGTGTTTTTGAGAAGTTAGATGTGATTGAGG
GTAATGGTGGTGTTGGAACTGTTCTTAACATTGTTTTCCCTCCAGGTGCTGTGCCCCGTTGTTACAAAGAG
AAATTCATTAATATCGACAACAAGAAGAGGTTAAAAGAAGTGATTATGATCGAAGGAGGCTATTTAGACAT
GGGATGTACATTTTACATGGATAGGATCCATGTTATAGCGGAAACTCCTAATTCTTGTGTTATCAAATCAT
CTATCATTTACGACGTGAAAAAAGAGTATGCCGAAGCTATGTCTAAACTAATCACAACCATACCTTTGAAA
TCCATGTCTGAAGTCATCGCTAATTACGTTCTCAAGAATCAATCTGTGATAAGAAAGGAAGTTACATATGA
ACTGCAAGTGCCAACCTCAGACTGA

>AMENCS2
ATGAAGTTTGAGCTAGTAAATGAGTTAGAGGTGCCTGCCTCAGCAAATGATGTATGGGCAATTTATAGCTC
ACCTGATTTCCCTAAACTCCTTACGAAGTTGGTTCCAGGTATTCTGGAGAGTGTAGAATACGTCGAAGGCG
ATGGTCATCTTGGAACTGTTATTCATCTTGTATACGTTCCTGGGAGTGTGCCACTTAGTTACAAGGAAAAG
TTTGTGACGATCGACCACGAAAAACGTTTGAAAGAGGCAGTGCATGTCGAAGGAGGATTCCTAGAGATGGG
TGTAACATTTTACATGAACAGCTTCGAAATTATTGAGAAAGGTTCGGATTGTTGCATAATTAGGTCAATGA
CTAAGTGCGAAATTGAGGATAAAGAAATCATGAACCTAATTTCTCATATTAGTGTTGCAAATGTGACCGTC
CTTGCAATGACTATCTCAAAATATGTTCAACAACACAAGAAATAA

>TFLNCS2
ATGAAGATGGAAGTTGTATTTGTTTTCTTTATGATCTTAGGAACAATTAATTGCCAGAAACTGATTCTGAC
AGGTAGGCCATTTCTTAACCGCCAAGGCATAATAAACCAAGTGTCTACAGTGACAAAAGGGGTTCATCATG
AGTTGGAAGTTGCTGCTTCGGCTGATGATATATGGAGTGTTTATAGCTGGCCTGGTTTGGCAAAGCATCTT
CCTGACTTGCTCCCTGGCGCTTTTGAAAAGCTCGAAATCATTGGTGATGGAGGTGTTGGTACCATCCTAGA
CATGACATTTACACCAGGTGAATTTCCTCATGAATACAAGGAGAAATTTATTTAGTCGATAATGAGCATC
GTTTAAAGAAGGTGCAAATGATCGAGGGAGGTTATCTGGACTTAGGAGTAACATACTACATGGACACAATC
CAGGTTATTCCAACTGGTACAAATTCGTGTGTCATTAAATCCTCAACTGAGTACCATGTGAAACCTGAGTT
TGTCAAAATCGTTGAACCACTTATCACTACTGGTCCATTAGCTGCCATGGCGGAAGCCATCTCAAAACTTG
TTTTAGAACACAAATACAAAAGCAACTCAGATGAGATTGATGCCTCAAAAAACAATCTGAAGATGGTGATT
AATATGTAA

>TFLNCS3
ATGAAGATGGAAGCTACTGTATTTGTTTTTTTAATGTTCTTAGGAACAATAAATTGTCAGAAATTGATTAT
GGCAGGTAGGCCGTTTCTTCATCACCAGGGCATAATAAACCAGGAGTTTACAGTTACAAAAGTGCTTCATC
ATGAGTTGGAAGTTGCTGCTTCGGCTGATGATATATGGGGTGTTTATAGCTCGCCTCACTTGGTTTTTCAT
CTCACTGACTTGCTCCCTGGTGCTTTTGAAAAGGTCCAAGTCATTGGTGATGGAGGTGTTGGTACTATTCT
AGACATGACATTTGCACCAGGTGAATTTCCTCATGAATACAAGGAGAAATTTATTGTAGTCGATAATGAAC
ATCGTTTAAAGAAGGTGCAAATGATCGAGGGAGGTTATCTGGACTTAGGAGTAACATACTACATGGACACA
ATCCAGGTTGTTCCAACTGGTACAAATTCGTGTGTCATTAAATCCTCAACTGAGTATCATGTGAAACCTGA
GCTACTCAAAATCGTTGAACCACTTATCACCACTGGTCCAGTAGCTGCCATGGCGGAAGCCATCTCAAAAC
TTGTTCTAGAATACAAATACAAAAGTCACTCAGACGAGATTCATGCTGGCCTCAATAACAATCTAAAGATG
GTGATCAATAATATATAA

>TFLNCS4
ATGAGGAAGGAACTAACACATGAGATGGAGGTACCTGCCTCAGCTGATGCTATTTGGGCAGTCTATGGTTC
TCCTGATATTCCCAGGCTCCTCAAAGAAGTTTTGCTCCCAGGTGTCTTTGAAAAGCTGGATGTTATTGAAG
GTGATGGTGGTGTTGGTACCGTTCTCGATATTGCTTTCCCACCAGGAGCGGTGCCGCGTGCTTACAAGGAG
AAATTCATGAAGGTCAATCACGAAAAGCGATTGAAAGAGGTGGAGATGATCGAAGGAGGGTATTTGGATAT
GGGTTGTACATTTTACATGGACAGAATCCATGTCGTAGAGAAAGGTCCTAATGCCTGTGTTATTGAATCGG
CTATTATATATGAAGTGAAGGATGAATTCGCCGACGTTGTTGTTCCTCTAATCACAACTGAACCACTGGCT
AGCATGGCTGAGGTCATCTCAAACTATGTTCTAAAGAATCAATTTCGCGTGTTCGGCTATGTAATTAAACC
TAAACTCGGATTAAGTCTTTTGCTCTGCTTCATTCTCTGCCTCGTTTTACTTGGAGGGTTGTT
GATTGGAGGTGTTCCACTCTAA

>TFLNCS5
ATGAGGAAGGAACTTACAAATGAGATGGAGGTGGCTGCGTCTGCTGACGAAATTTGGGCAGTCTACAGCTC
CCCCAACCTCCCCAAACTCATCGTTCAATTACTTCCTGCTGTCTTTGAAAGGATATATATCCTTGAAGGAG
ATGGTGGTGTTGGTACCGTTCTCTATATTTTATCTCCTCCAGGATCGGTTCCGCGTAGTTACAAGGAGAAG
TTCATTACAATCGATCATGAGAAGCGTCTGAAGGAGGTGCAAGAGATCGAAGGAGGGTACTTGGACATGGG
CGTTACCTTTTACATGGACACCTTCTACATCTTAGAGAAAGGTCCTGATTCCTGCATCATCAAATCCATGA
CTACCTACGAAATCAAGGATGAGCTGGCCGATAAAGTTGCTTCTCTTATTAGCATTGATTCACTAGTTGGC
ATGGCTAAAGCCATCACAAAATATGTCCTTGATCAGAAGAAAGCTGCTGTGGATTCTTCTGCCTAG

>BTHNCS1
ATGGTAGTGGCTGCCTCAGCTGATGATGTTTGGGCAATCTATAGCTCCCATGATCTGCCCAAACTCATTGT
GAAGTTGCTTCCAAGTGTCTTTAAGAGCATAGAAATTGTTGAAGGTGATGGAGGTCTTGGTACAGTTTTGG
ATGTTAAATACCCTCCAGGATCAATACCACTACATTACAGGGAGAAATTTATAACAATCGATAATGAAAAA

CGTCTTAAAGAAGTGAGACAAATCGAAGATGGACTTCTGGCTTTAGGATGCACATTCTACATGGACAGCTT
CCATATCCTTGAGAAAGATTGTCACCATGAATTCTTCCATATCCATGAGAAGAACTGTCATAAGAAATGTA
TCATCAAGTCGACTACAGTATATGAGATACCTGATGAGTTAGCTTATAAAATCGAACCTCTAGTCACCATT
GATTCCCTGGTTGGTATGGCTCATGCCATCTCAAAATATGTTCTTGACAAATCGTGTTAA

>CCHNCS1
ATGTACTACTTCCTGGAGTTCTTCGAAAAACTAGATGTCATTGAAGGCAATGGAGGCGTCGGCACTGTTCT
TGACATTGCTTTTCCTCCAGGGGCAGTACCACGTAGCTACAAGGAGAAGTTCGTAAAGGTTGACCACAAGA
ACCGTTTGAAGGAAGTCGTGATGATCGAAGGAGGATATTTAGATCTTGGATGTACATTTTATATGGATAGA
ATCCATGTCTTACCAAAAGGTGCAAATTCATGCGTAATCAAGTCGACCCTCATTTACGAAATTCCAGATGA
GCTTGTCGACTCTGTTGGTTCTCTTATGTCTACTGAACCACTAGCAAGCATGGCAAAAGTCATCTCGGATT
ACGTTCTCAAGCAGAGAAAGATGACAGCAAACAAAATATTGAGGAAGGAATTAAAAACAGAGATGGAGGTG
GCTACTTCAGCTGATTCTATATGGGCAGTCTACGGTTCCCCTGACATTCCTAGACTCCTCAGAGATGTATT
ACTTCCTGGAGTTTTTGAGAAACTGGATGTCATTGAAGGCAATGGAGGTGTCGGCACTGTTCTTGATATTG
CTTTTCCTCCAGGGGCGGTACCTCGTACTTACAAAGAGAAGTTCGTAAAGGTTGATCACAAGAACCGTTTG
AAGGAAGTCGTGATGATTGAAGGCGGATATCTGGACTTAGGATGTACATTTTATATGGATAGAATCCATGT
CTTACCAAAAGGTCCTAATACATGCGTAATCAAATCGACTCTTATCTATGAAGTTCCAGACGAGTTCGCTG
ATGCAGTTGGTTCTCTTATCTCCGTTGAACCACTAGCAAGCATGGCAGAAGTAATATCAGGTTATGTTCTC
AAGCAGAAGAAGGAAGCAAAGAAAATATTAAGGAAGGAATTAACACACGAATTGGAGGTGCCTACTTCAGC
TGATTCAATATGGGCAGTCTATGGTTCCCCTGATATTCCAAGATTGCTCAGAGATGTATTACTTCCTGGTG
TGTTTGAAAAGCTAGACATCGTGGAAGGCAATGGAGGTGTTGGTACTGTTCTTGACATTGCTTTTCCTCCA
GGGGCGGTACCTCGTAGTTACAAGGAGAAGTTTGTAAAGGTTGATCACGATAAGCATTTGAAAGAAGTTGT
GATGATCGAAGGAGGATATTTGGATCTAGGATGCACATTCTATATGGATAGAATCCATGTCCTACCGAAAG
GTCCTAATTCTTGTGTTATCGAATCGTCTCTTATTTATGAAGTCCGGGAAGAGCTCGCTGATGTCGTTGGT
TCTCTTATCTCAATTGAACCACTTGCTAGCATGGCAGAAGTCATCTCAAGTTATGTTCTCAAACAACAACT
CCGAGTGTTTGGAGTTGTAGTTCAACCAAGAGTAGGATTAAGTCTTTTGCTCTGCCTTATACTGTGTCTAG
TCATATTAGGAGGTCTTTTGATCGGCGGTGTTTCGATATAA

>CCHNCS2
ATGAGGAAGGAATTAAGACATGAATTGGAGGTGGCAACTTCAGCTGATTCTATTTGGGCAGTCTATGGTTC
CCCTGATATCCCAAGACTTCTCAGAGATGTATTGCTTCCTGGTGTCTTTGAAAAACTAGATGTTATTCAAG
GCAATGGAGGTGTGGGTACTGTTCTTGACATTGCTTTCCCTCCAGGGGCGGTTCCACGTACTTACAAGGAG
AAGTTCGTAAAGGTCGATCACAAGAATCGTTTGAAGGAAGTTGTAATGATCGAAGGAGGATATCTGGACCT
AGGATGTACATTTTATATGGATAGAATCCATGTCTTGCCCAGTGGACCTAATACATGTATAATCAAATCTA
CACTTATTTACGAAGTTCCAGACGAGCTCGCCTACTCCGTTGCTTCTCTAATCTCTGTTGAACCACTAGCA
AGCATGGCAGAAGTCATCTCAGGTTATGTGCTCAGGCAGAGAAAGATGACAACAAACAAAATATTGAGGAA
GGAATTGACAACAGAGATGGAGGTGCCCACTTCAGCTGATTCAATATGGGCAGTCTATGGTTCCCCTGATA
TTCCTAGACTCCTTAGAGATGTATTACTTCCTGGAGTTTTCGAAAGGCTGGATGTCATTGAAGGCAATGGA
GGTGTCGGCACTGTTCTTGATATTAGTTTTCCACCAGGGGCGGTACCACGTAGTTACAAGGAGAAGTTTGT
GAAGGTTGATCACAAGAACCGTCTGAAGGAAGTTGTGATGATCGAAGGAGGATATCTAGATCTAGGATGTA
CATTTTATATGGATAGAATCCATGTAATACCAAAAGGCCCTAATTCATGCGTAATCAAGTCGACCCTCATT
TATGAAATTCCTGGTGAGCTTGTGGACTCTGTTGGTTCTCTTATGTCTACTGAACCACTTGCAAGCATGGC
AGCAGTCATCTCGGATTACGTTCTCAAACAGAGAAAGATGACAGCAAACCAAATATTGAGGAAGGAATTGA
CAACAGAGATGGAGTTGGCTACTTCAGCTGATTCTATTTGGTCAGTTTATGGTTCCCCTGATATTCCTAGA
CTCCTCAGAGATGTATTACTTCCTGGAGTTTTCGAAAGACTAGGATGTCATTGA

>CCHNCS3
ATGATGAGGAAAGAACTAGTACATGAAAAGGAAGTGTGTGCATCAGCTGATGCAGTATGGGGAGTGTATAG
TTCACCCAATATTCCAACACTCCTTAGAGATAAATTACTTCCTGGTATGTTTAAAAGGCTTGAGATACTTG
AAGGTGATGGAGGAGTTGGTACAATTCTCCTCCTTGAGTTCAATAATCCAGCGATTATACCACATACATAT
CTTGAAAAGTTCATGAAGCTAGATCATGAGAAGCGTTTGTTGGAAGTTGAGGTGGTCAAAGGAGGATATTT
GGATTTAGGATGTACATTTTATATGAGTAGAATACACATCTTGGAGAAAGGTCCTAATTCATGCGTAATCG
AATCGACTCTTATTTTCGAAGCACCGGAGGAACTCATGGAATATGTTAGTCAATATGCAAACCTTGAATCA
TTAATTAGCATGGCAGAAGTTATATCAAAGTATGTTCTTGAGCAGCAATTCCGAGTTTTCGGAGTTGTTGT
CAAGAAACTGAAATTGGGATTATCAACTATTGTGTTGCTATGCATTTTTATCTTTCTGGTTATTGTATTAG
GAGGTCTTTGGATTGAAGGTGTTTCAATCTAA

>CCHNCS4
ATGAGGAAGCATCTTGTTAACGAGTTGGAGGTTGTTGTTCCAGCCGACACTCTTTGGGCAATTTACAGCAC
CACTCAATTCCCAAAGCTAATTGTTCAATTGCTTCCCATTGTTGTCCAAAATATAGAAATTGATGGCGACG
GAAGTCTTGGTACTGTTTTGAATGTTATTTTTGTTCCAGGATCGGTTCCATTGTCTTACAAAGAGAAAATC
GTGACGATTGATCATGAGAAGCGTTTGAAGGAAGTGGTGCAGATCGAAGGAGGATATTTGGATTTAGGATG
TTCATTTTACATGAGCAGCTTTCAAATCTTGGAGAAAGGTCGTGATTCTTGCATCATCAAATCCATGGTTA
CATACGAGCTGGCTAAGGATGCTGATCCTAGTGTTGCTGATTTGGTCACCATTGCTGCACATGCTGCCATA
GCTCAAGTCATCTCTAAGTATGTTCTTGACAAACAAGTAGCCGCAGCTCCATAA

>CCHNCS5
ATGAGGAAGGAACTCACAAATGAGTTGGAGGTCGCAGCCCCTGCTGATGCTGTCTGGGCAGTTTACAGCTC
CCCGGATCTCCCAAAAATCATAGTTGAATTACTTCCTAGTGTCTTCGAAAAGATTGAAATCGTTGAAGGAG
ACGGAGGTGTTGGTACCGTTCTGTACGTTGTTTTTCCTCCAGGATCAGTTCCATTAACTTACAAGGAGAAG
TTCGTGACGATTGATCACGAGAAGCGTCTGAAGGAGGTGCTACAGATTGAAGGAGGATATTTGGACCTAGG
GTGTACGTTTTACATGGACAGCTTCCATATACTAGAGAAAGATTGTGATTCATGCATCATCAAATCCATAA
CAGCATACGAAGTCAGGGATGATGTTGTTGATAATGTTTCCTCTCTTATCTCCATTGATTCGCTCGCTAAC
ATGGCTGAAGCCATCTCAAAGTATGTCCTTGAGAAACAAGAAGCCGCAACTAAGCATGGACATGGAGATGA
TAGGGAAAGGACTGGTCTTTGTTGGCCTTTCAATTGTTTGGGTTAA

>NDONCS1
ATGAGGAAGGGAATTGTTTTCCTATTTCTAGTTTTCTTAGGATGTGAAGTTTCACAAGGAAGGCAACTGCT
AGAGTCGAGATTATTTAGGAAATCTACGATACAAAAAGTTCTTCACCATGAGTTGCCAGTTGCTGCGTCGG
CCCAAGAAGTGTGGGATGTTTATAGCTCGCCCGAATTGCCAAAACACCTACCAGAAATACTTCCAGGCGCA
TTCGAGAAAGTTGTAGTTACCGGGGATGGTGGTGTTGGTACTGTACTTGAAATGGTATTTCCTCCAGGAGA
AGTACCCCGTAGTTACAAGGAGAAATTTGTGTTGATTGATGACGAACAGCTTTTGAAGAAGGTCGAAATGA
TTGAAGGTGGATATTTGGACATGGGATGTACGTTTTATATGGACACAATCCAAATCGTTCCAACAGGTCCT
GATTCATGCATAATCAAATCCTCAACTGAATACTACGTTAAACCTGAATTTGCGGACAAGGTTGTACCTCT
TATCAGCACAATCCCATTGCAAGCCATGGCCGAAGCTATCTCGAACATTGTTCTAGCAAACAAAGCCAAGA
ACAAGAGTATTATCATCGAAATATAA

>NDONCS2
ATGGTATTTCCTCCAGGAGAAGTACCCCGTAGTTACAAGGAGAAATTTGTGTTGATTGATGACGAACAGCT
TTTGAAGAAGGTCGAAATGATTGAAGGTGGATATTTGAACGATTTGGATTGTGTCCATATAAAACGTACAT
CCCATGTCCAAATATCCACCTTCAATCATTTCGACATGGGATGTACGTTTTATATGGACACAATCCAAATC
GTTCCAACAGGTCCTGATTCATGCATAATCAAATCCTCAACTGAATACTACGTTAAACCTGAATTGCCGGA
CAAGGTTGTACCTCTTATCAGCACAATCCCATTGCAAGCCATGGCCGAAGCTATCTCGAACATTGTTCTAG
ACAAAACAAAAGACCAAAGAAACAAAGAAGTAATTAATACAAATACGAAAAATAATAAAATACATCATCGC
TATGTCGCTACCATTGTTATAATCAGATAA

>NDONCS3
ATGAGGAGTGGAATTGTTTTCCTGGTTCTATTTTTCTTAGGATGTGAAATTTCGCAGGGAAGACAATTACT
GGAGTCGAGACTATTTAGGAAGTCTACAATACGAAAAGTGCTTCACCATGAGTTGCCAGTAGCTGCGTCGG
CCCAAGAAGTGTGGGACGTTTATAGCTCGCCGGAATTGCCAAAACACCTACCAGAAATACTTCCAGGCGCA
TTTAAGAAAGTTGTAGTCACTGGAGATGGAGGTGTTGGTACGGTAATTGAAATGGTATTTCCTCCAGGAGT
AGTACCGCACCGTTACAAGGAAAAGTTTGTTCTAATCGACGATGAGAAATTTTTAAAGAAGGTTGAAATGA
TCGAAGGTGGATATTTGGACATGGGATGTACGTTTTACATGGACACAATCCAAATCGTTCCAACAGGTCCT
GATTCATGCATAATCAAGTCCTCAACTGAGTACTATGTTAAACCTGAATTCGCGGATAAGGTTGTACCTTT
GATCAGCACTGTTCCGTTGCAAGCCATGGCTGAAGCTATTGCGAAAATCGTTCTAGAGTTCAAAGCCAAGC
ACAAGGGGTTTATCGAAATATAA

>NDONCS4
ATGGAAGTGGCTGCGTCGGCGGGTGATATTTGGGCTGTTTACAGTTCCCCTGATCTGCCTAGGCTTATCGT
CCAATTACTCCCCACTGTGTTTGAAAAGATAGACATTGTAGAAGGCGATGGAGGTGTTGGTACTGTTTTAC
ATATTACATTTCCTCCCGGATCTGTACCGCTTACTTACAAGGAGAAATTTGTGACGATCGATAATGCAAAC
CGTTTAAAAGAAGTACTACAGATCGAAGGTGGATATTTGGAGTTGGGATGTACTTTCTATATGGATAGCTT
CCAGATCTTCGAGAAAGGTATTGATTCATGCATCATCAAATCAATGACTACTTATGAAGTACCAGATGAGC
TTGCAGACAAAGTTGCTCCTCTTATCTCCATTGATTCCCTCGTTCCCATGGCTGAAGCCATCTCAAAATAT
GTTATTGAGAAGCGTCATTAA

>CTRNCS1
ATGATCAAGAAGGAACTCAAACACGAGATGAGAGTGGCTGCCTCTGCAGATGATATATGGGCAGTTTACAG
CTCACCTGATTTGCCCAATCTCATCCTCAGATTGCTACCTAGTGTTTTCGACAACATCGAAATCGTCGAAG
GCAACGGAGGAGTCGGAACTGTTCTCCACCTCACTTTTCCTCCAGGTTCAGTACCACTTTCATACAAGGAG
AAGTTCGTGACTATTAATGGCAACAAACGTTTGAAGGAGGTGAAGCAGATTCAAGGAGGGTATCTTGACAT
GGGCTGCACATTCTACATGGACAGCTTTCATATAGAAGAGAAAGGTTGTGATTCATGTGTGATCGTGTCGA
AGACCGAGTACGAAGTACCTAATGAGGAGATAGCAAACCAAGTTGAGCTTTATATATCTATTGATTCACTA
GCTAGCATGGCCCAAGGCCATCTCGGATTATGTTCTTAG

>HCANCS1
ATGAAGATGGCAATTTTGTTTGTGTTCTTAATGTTCTTGGGAAAGATGAATTCTGAAGGCTTGCACTTGAG
CGGGAGGCCGCTTCTCCGGGCGATAATATCCGACAAGCCCAATGTAATCAAAGTGCTTAAACATGAGTTGG
CAGTACCTGCATCCGCGGACAAAGTTTGGGCAGTCTATAGCGCGCCCACGTTGGCCTTCCATCTCAGCGAC
TTACTTCCCGGTGCCTTTGAAAAGGTGGAAGTTTTTGGTGATGGAGGTGTTGGCACTATCATCGACATGAC
ATTTGCCCCAGGTGAATTTCCCCATGAATACAAGGAAAAATTCATCTTGATAGATGGCAAACAACGTCTAA
AGAAAGTACAAATGATCGAAGGAGGTTATTTGGATCTAGGAGTTACATACTATATGGACACCATCCATGTC
GTCCCAACAGGTTCCAATTCATGCATCATCAAATCATCTACTGAATACCATGTAAAACCTGAGGCCGCAAA
GCTAGTCGAACCTCTTATCACAACTGAGCCATTAGCTGCCATGGCTGAAGTTATCACAAAGATTGTCTTAG
AGAACAAAAGCAAGAGCTCCGAAGAAAATCAGTCATCAGAAGCCATATAA

>NSANCS1
ATGGTTCAGTTCAGCAGAGAAAGCAAGCAAATAAGCATTATCTCTGACGAAGAAGAAGGAGGAGAAGAAGA
AACTAAGGAGAAGAAGATGATGAAGGTACAAGTAGCACTTGCTTTCTTACTAATATTAGGTGCTGCAAGCT
GCCAAGAACTCATACTGCAGGGAAGGCCGCTTCTTGGTGGTGCCCGCGCGTGGGGTACCAAATCCATAAAG
AAAGAGCTGAAACACGAGTTCAAAGTTGCTGCTTCAGCTGATGAGGTTTGGAGTGTGTATAGCGCACCAGA
ACTCTGCAAACATCTCACTGATCTTCTCCCAGGTGCATTTGAGGATGTTGAAATCATTGGTGACGGAGGCG
TTGGTACCATTCTTCACATGATTTTCCCTCCAGGTGAATTCCCGCATGAATACAAGGAGAAGTTTGTGGTG
ATTGATGACAAGCAACGGTTAAAGAAGGTAGAAATGATCGAAGGTGGGTATCTGGATATTGGAGTGACTTA
CTATATGGACACCATCCATGTTGTGCCCACTGGTTCAGACTCATGTGTGATTAAGTCATCCACAGAATACC
ATGTAAAACCTGAGTTTGAGAAAATTGTGGAACCACTTATTACTACAGTTCCATTAGCTGCCATGGCTGAA
GCTATCGCCAAGATTGTTCTAGACAACAAAACTCATTCCATTACAATCTGA

>NSANCS2
ATGGTGAAGATACAGTTAGTGCTTGCTTGTTTACTACTAGTAGTAGGTGCTGTAAATTGCCAAAAGCTTAT
ATTGCAGGGGAGGCCACTTCTTGGTGCCTGGGCGTGTGGTACCATCAAGAAAGTGCTGAAACATGAGTTCA
AAGTAGCTGCTTCAGCTGATGAGGTGTGGAGTGTGTACAGCTCACCAGAACTCTGCAAACATCTAACTGAT
CTTCTCCCAGGTGCATTTCAGGATCTTGAAATTATTGGTGATGGAGGCGTTGGTACCATTCTCCACATGAC
TTTTCCACCAGGTGAATTCCCACATGAATACAAGGAGAAATTTGTGTTAATTGATGACAAGCGAAAGTTAA
AGAAGGTAGAAATGATAAAGGGTGGCTATTTGGATATTGGAGTGACTTACTATATGGACACCATCCATGTT
GTGCCCACTGGTTCAGATTCATGTGTGATCAAGTCGTCCACAGAATACCATGTAAGACCTGAGTGTGAGAA
AATAGTGGAACCACTTATTACCACTGAACCATTAGCTGCCATGGCTGAAGCTGTCTCCAAGATTGTTCTAG
ATGCCAAAATTCATTCCATAATAACAATCTGA

>MCANCS1
ATGATCAAGAAGGAGCTCAAGCATGAATTGGAGGTAGCTACCTCTGCGGACGAAATTTGGGAAGTATACAG
CTCTCCTGATTTGCCCATCCTTATTGTTAAATTGCTTCCAAGCGTGTTCGAAAAGATCGAAATCTTGGAAG
GCGATGGAGGCGTTGGTACTGCTCTTCGACTCACATTCCCAATAGGGTCAGTGCCCCTTACTTACAAGGAG
AAGTTTGTGACCATTAACGACTGGAAACGATTGAAGGAGGTGAAGCAAATCGAAGGAGGGTACCTTGATAT
GGGCTGCACATTCTACATGGACAGTTTTCATATTTTACGAAAAGGTCCTAAGTCGTGTGTGATTGTGTCTA
AGACTGAGTACGAAGTACCTAATAAGGAGATAGCAAGCAAAGTTGAACCTTATATATCTATTGATTCACTC
AGGAAGATGGCAACTGCCATCTCGGATTATGTTCTGAACAGGGCGACCAGGAAGGAAGTGAAGCACGAATT
AGAGGTGGCTGCCTCGGCAGACGATGTGTGGGAGGGCTACAGGTCACCTGACGTGGGCAGCCTAATTTGCC
CACATGTGTTCGAAAAGATCGAGCTTGTAGAAGGTGATGGCGGTGTTGGCACTATTCTGCAGATCACGCAT
CCACCAGGATATGTGCCACATACTTACAAGGAGAAGTACGTGACGCTAGATGATCACAAACGTTTGTTGGA
GGTAGAGCAAATCGAAGGAGGATACCTTGAGATGGGCTGCACATTTTACATGGACAGCATTCACGTTGTAA
AGAAAGGTGACAACTCATGTGTCATCGTGTCCAAGGCAAAGTACGAAGTTCCCAAGGAGTTAGCAAGCCAA

GTTGAACCTTACATTGCCGCTGATGCAGTTGCAAACATGGCCAGAATAATCTCCAATAATGTTCTAGAGAA
GAAGAAATCATGA

>XSINCS1
ATGAGGATGGAAGTTGTTCTAGTTGTTTTCTTACTGTTCATAGGTACTGTAAATTGTGAAAGAATGATATT
CAGTGGGCGTCCTCTCCTCCATCGCGTAACAAATGAGGAGACTGTAATCCTTTATCATGAGCTGGAAGTAC
CTGCTTCGGTGGATGAACTGTGGAGTGTCGAAGGTTCGCCTGAGTTGGGCAAGAATTTGCCTGACCTGCTC
CCTGGTATATTTGCAGACTTCAAAATTACTGGTGACGGAGGTGAAGGTTCCATCCTGGATATGACATTCCC
CCCAGGTCAGTTTCCACATCATTACAGGGAGAAGTTCGTGTTTTTCGATCACAAGAATCATTACAAGTTAG
TACAAATGATCGACGGTGATTTTTTCGATCTAGGTGTTACATACTATATGGATACAATCCGTGTTGTTGCA
ACAGGCCCTGATTCATGTGTCATCAAGTCTTCAACGGAATATCATGTAAAAGTTGAGTTTGCCAAGATCGT
CAAACCACTTATTGACACTGTACCACTAGCTATAATGTCTGAAGCGATTGCAAAGGTTGTTCTTGAGAAGA
AATACAAGAGATCAGAGTAA

>PSONCS3
ATGAGGAAAGTAATCAAATACGATATGGAGGTAGCTGTCTCAGCTGATTCAGTTTGGGCAGTTTACAGTTC
ACCGGATATTCCTAGACTTCTCAGAGACGTTCTACTTCCCGGTGTCTTCGAGAAGTTAGATGTTATTGAAG
GGAATGGCGGCGTCGGAACAGTTCTTGACATTGTTTTCCCTCCAGGTGCGGTTCCTCGAAGTTACAAGGAG
AAATTTGTCAATATCGATCGCGAAAAGCGATTGAAAGAAGTGATCATGATCGAAGGAGGATACCTGGACAT
GGGATGCACATTTTACTTGGATAGGATCCATGTAGTGGAGAAAACCAAGAGCTCATGCGTTATTGAATCGT
CTATTGTTTACGATGTGAAAGAAGAGTGCGCCGATGCCATGTCTAAATTGATCACAACTGAACCATTGAAG
TCCATGGCGGAAGTCATTTCTAATTACGTTATTCAGAAAGAATCATTTTCTGCCAGAAACATTCTAAGCAA
GCAATCTGTAGTGAAGAAGGAGATTCGATACGACCTGGAGGTACCAATCTCAGCTGATTCTATCTGGTCAG
TTTACAGCTGCCCTGATATCCCTCGGCTTCTTAGAGATGTTCTACTTCCTGGTGTGTTCGAGAAATTGGAT
GTCATTGAAGGGGATGGTGGTGTTGGGACTGTTCTTGACATTGTCTTCCCTCCAGGTGCAGTTCCTCGAAG
TTACAAAGAGAAATTCGTTAACATTGACCGCGAGAAGCGATTGAAAGAGGTTATCATGATCGAAGGAGGAT
ACTTGGACATGGGATGCACGTTTTACTTGGACAGGATCCATGTAGTGGAGAAAAGCCTGAGCTCGTGTGTT
ATCGAATCGTCTATTGTTTATGAAGTAAAAGAAGAGTATGCTGATGCCATGTCTAAATTAATCACAACTGA
ACCATTGAAGTCGATGGCGGAAGTCATCTCTAATTACGTTATCCAGAGAGAATCATTTTCCGCAAGAAACA
TTCTCAACAAAAAATTCTTTGGTGAAGAAGGAGATTCGGTATGACCTGGAGGTTCCAACCTCAGCTGATTCT
ATCTGGTCAGTTTACAGCTGCCCGGATATTCCTCGGCTTCTTAGAGATGTTTTACTTCCCGGTGTTTTCCA
GAAATTGGATGTTATCGAAGGGAATGGTGGTGTTGGTACAGTTCTTGATATCGTTTTTCCTCCAGGTGCGG
TACCTCGTAGTTACAAGGAGAAATTTGTGAACATCAACCACGAAAAGCGATTAAAAGAAGTGATTATGATC
GAAGGAGGATATTTAGACATGGGATGCACATCTTACCTGGACAGGATCCATGTAGTTGAAAAAAACCTCTAA
ATCTTGTATCATTAAATCTTCTGTTGTCTATGAAGTGAAGCAAGAGTGTGTTGAGGCAATGTCTAAGTTGA
TCACGACGGAACCATTGAAGTCGATGGCAGAAGTTATCTCTAATTACGCTATGAAGCAACAATCTGTTTCT
GAGAGAAACATTCCTAAGAAGCAATCTCTACTGAGGAAGGAAATTACTTATGAAACGGAGGTGCAAACTTC
TGCTGATTCAATTTGGAACGTCTACAGTTCTCCTGACATCCCTCGACTACTTAGAGATGTTCTGCTTCCTG
GTGTTTTTGAAAAGCTAGATGTCATTGCAGGCAATGGTGGAGTTGGTACGGTACTGGATATTGCCTTCCCT
CTAGGTGCAGTGCCACGGAGGTACAAGGAGAAATTTGTGAAGATCAACCATGAGAAGCGATTGAAAGAAGT
GGTGATGATCGAAGGAGGATACTTAGACATGGGGTGCACATTTTACATGGACAGGATCCATGTCTTTGAGA
AAACCCCAAACTCATGTGTTATCGAATCCTCGATCATTTACGAAGTTAAAGAAGAGTATGCTGGTAAAATG
GCTAAGCTAATCACAACTGAACCATTGGAATCCATGGCAGAAGTCATCTCTGGTTATGTTCTTAAGAAACG
ACTCCAAGTATTCGGATTCGAGATTAAGCCAAAATTAAGATTCAATCTTTTGCTATGTTTGATTATCTGTC
TGGTTATAGCTGGAGGTATGTTTGTTGCTGGTGTTCCACTCTAA

RT-PCR amplified cDNA sequences

>SCANCS1
ATGAGGAAGGAACTGACACACGAGATGGAGGTGCCTGCCTCAGCCGATGCTATTTGGGCAGTCTACAGTTC
ACATGATATTCCAAGGCTGCTCAAAGAAGTTTTGCTTCCTGGTGTTTTTGAAAAGCTAGATGTCATTGCAG
GTGATGGTGGTGTTGGTACTGTTCTCGACATTGCTTTCCCTCCAGGGGCGGTACCGCGTCGTTACAAGGAG
AAATTCGTGAAGATCAATCACGAGAAGCGATTGAAGGAAGTGGAGATGATCGAAGGAGGGTATTTGGATAT
GGGGTGTACATTTTATATGGACAGGATTCATGTCGTAGAGAAAGGTCCTAATTCATGCGTTATCGAATCGG
CGATTATTTACGTAGTGAAGGACGAATGCGCCGATGTCGTCGTTCCTCTAATTACGACTGAACCACTGGCT
AGCATGGCGGAGGTCATCTCAAATTACGTTCTAAGGAAACAAATCCGATTGTTTGGATACGTAATTAAACC
AAAATTAGGGTTAAGTATTTTGCTCTCCTTGATTCTCTGCCTAGTTATACTAGGAGTGTTATTGATTGGAG
GTGTTCCATTCTAA

>NDONCS3
ATGAGGAGTGGAATTGTTTTCCTGGTTCTATTTTTCTTAGGATGTGAAATTTCGCAGGGAAGACAATTACT
GGAGTCGAGACTATTTAGGAAGTCTACAATACGAAAAGTGCTTCACCATGAGTTGCCAGTAGCTGCGTCGG
CCCAAGAAGTGTGGGACGTTTATAGCTCGCCGGAATTGCCAAAACACCTACCAGAAATACTTCCAGGCGCA
TTTAAGAAAGTTGTAGTCACTGGAGATGGAGGTGTTGGTACGGTAATTGAAATGGTATTTCCTCCAGGAGT
AGTACCGCACCGTTACAAGGAAAAGTTTGTTCTAATCGACGATGAGAAATTTTTAAAGAAGGTTGAAATGA
TCGAAGGTGGATATTTGGACATGGGATGTACGTTTTACATGGACACAATCCAAATCGTTCCAACAGGTCCT
GATTCATGCATAATCAAGTCCTCAACTGAGTACTATGTTAAACCTGAATTCGCGGATAAGGTTGTACCTTT
GATCAGCACTGTTCCGTTGCAAGCCATGGCTGAAGCTATTGCGAAAATCGTTCTAGAGTTCAAAGCCAAGC
ACAAGGGGTTTATCGAAATATAA

>PBRNCS3
ATGGATATCATAGAAGGGGATGGTGGAGTTGGTACTGTTCTTGATGTTGTTTTCCAACCTGGTGCGGTGCC
TCAAAGTTACAAGGAGAGATTTGAGACCGTGGACCACGAGAAGCGAATACTGGAAGTGAGAATTATCCAAG
GAGGATACTTAGAAATGGGTTGCACATTTTACCTGAATAGGATGCATGTTATTGAAATAACCTCTAAATCT
TGTGTTATTAAATCTTCGGTTATCTACGACGTGAAAGAAGAGTGTGCTGATGCAATGTCTAAGTTAATCAC
AACCATACAGTTGGAGTCAATGGCCAAAGTGGTCGCTGATTATGTTCTTAAGAAACAATCTGCTTCTGACA
CAAGCATTCCTAAGAAGCAGTCTCTAATGAGGAAAGAAATTACACATGAGATGGAGGTGCAAACCTCAGCT
GATTCGATTTGGGACATCTACAGTTCTCCTGACATCCCTCGACTACTTAGAGATGTCCTGCTTCCTGGTGC
TTTCGAAAAGCTACATGTCATTCAAGGCAATGGTGGGGTTGGTACTGTACTGGACATCGCTCTCCCTCTAG
GTGCAGTGCCACGAAATTACAAGGAGAAATTTGTGAAGATCAACCACGAGAAGCGACTAAAAGAAGCAGTT
ATGATTGAAGGGGGATACGCAGACATGGGGTGCACATTTTACATGCACAGGATCCATGTCCTAGAGAAAAC
ACCAAAGTCGTGTGTCATTGAATCCTCCATCGTTTACGAAGTGAAAGAAGAGTATGCTGATAAAATGTCAA
AGCTAATCACAACAGAACCATTGCAGTCCATGGCAGAAGCCATATCTAGTTATGTTCTTAAGAAACAGTTC
CAAGTATTTGGATTAGAGGTTAAACCGAAATTAGTATTAAGTCTATTTCTATGTTTGATCATCTTTTTGGC
TATAGTTGGTGGTTTTTTGATTGGAGGTCTAAAAGCATAA

>ECANCS1
ATGATCGGAGGATTCTTAGACATGGGATGTACATTTTACATGGACAGGATTCATGTCATAGCGAAAGGTCC
TAATTCATGTATTATCAAATCAACTCTTATCTACGAAGTGAAAGAGGAATATGCCGATGCCATGGCTTCTC
TAATCACCATAGAACCACTAGCTAGCATGGCAGAAGTTGTTGCAAATTACGTTCTTCATCAACAAGTCCGG
GTGTTAGGATCCGTGAAGAGGAAGGAACTTACGCATGAGTTGGAAGTTGCTGCACCAGCTGATGCTATTTG
GGGTGTGTATAGCTCACCTGATATTCCGAGGCTTCTGAGGGATGTTTTGCTTCCGGGTGTTTTTGAAAAGT
TAGAAGTTATACAAGGAAATGGAGGTGTTGGTACTGTTCTTGAGATTGTTTTCCATCCAGGTGCAATTCCG
CGTAGGTACAAGGAGAAGTTTGTGACGATAAATCACAAGAAGCGACTGAAAGAGGTGGTCATGATTGGAGG
GTATCTAGACATGGGGTGTACACTTTATATGGACAGGATTCATGTAGTATCCAAAGGTCCTAATTCATGTG
TTATCAAATCGACACTCATTTATGAAGTTAAAGCAGAATCAGCAGATGCCATGGCTTCTACAATCACCATC
GACCCACTCGCTAGCATGGCACAGGTCATCTCAAATTACGTTCTCAAGAATCAAATGCAAGTCTTAGGATC
TGTTAAGAGAAGGGAATTAACACATGAGTTAGAGGTAGCTGCCTCAGCTGACGCTATTTGGGGAGTTTATG
GATCAAAAAGATATTCCAAGGCTTCTCAGGGATGTTTTGCTTCCTGGTGTTTTCGAAAAGTTAGAAGTCAT
TGA

>CMANCS1
ATGATTGAAGGAGGGTATTTGGATATGGGATGTACATTTTACATGGACAGAATCCATGTTGTAAAGAAAGG
TCCCAATTCATGCGTTATTGCATCGGCTATTATCTACGAGGTGAAGGAGGAATTTGTCGACGTCGTCGTTC

CTCTAATCACGACCGAACCATTGGCTAGCATGGCAGAAGTCATCTCAAATTACGTTCTTAAGAAACAACGT
CGTGTAAGGAAGGAACTAACATATGAGATGGAGGTGCCTACCTCAGCTGATTCAATTTGGGCAGTCTACAG
TTCACATGATATTCCAAGGCTCCTCAAAGAAGTTCTGCTCCCTGGTGTCTTTGAAAAGCTTGATGTCATTG
AAGGTGATGGTGGTGTTGGTACTGTTCTTGACATTGCTTTCCCACCAGGGGCGGTACCACGCACTTACAAG
GAGAAATTCGTGAAGATCAATCACGAGAAGCGATTGAAAGAGGTGGTGATGATTGAAGGAGGGTATTTGGA
TATGGGATGTACATTTTACATGGACAGAATCCATGTGCTAGAGAAAAGTCCTAACTCGTGCGTTATTGAAT
CTTCTATTATCTACGAGGTGAAGGAGGAATTTGCCGATGTCGTGGGTCCTCTAATCACGACCGAGCCACTA
GCTAGCATGTCAGAGGTCATCTCAAATTACGTTCTAAAGAAACAAATCCGCATGTTTGGTTATGTAATTAA
ACCAAAACTTGGTTTAAGTCTTTTGCTCTGCTTCATTCTCTGCCTCGTTTTACTTGGAGTTTTATTGATTG
GGGGTGTTCCACTCTAA

>CCHNCS2
ATGAGGAAGGAATTAAGACATGAATTGGAGGTGGCAACTTCAGCTGATTCTATTTGGGCAGTCTATGGTTC
CCCTGATATCCCAAGACTTCTCAGAGATGTATTGCTTCCTGGTGTCTTTGAAAAACTAGATGTTATTCAAG
GCAATGGAGGTGTGGGTACTGTTCTTGACATTGCTTTCCCTCCAGGGGCGGTTCCACGTACTTACAAGGAG
AAGTTCGTAAAGGTCGATCACAAGAATCGTTTGAAGGAAGTTGTAATGATCGAAGGAGGATATCTGGACCT
AGGATGTACATTTTATATGGATAGAATCCATGTCTTGCCCAGTGGACCTAATACATGTATAATCAAATCTA
CACTTATTTACGAAGTTCCAGACGAGCTCGCCTACTCCGTTGCTTCTCTAATCTCTGTTGAACCACTAGCA
AGCATGGCAGAAGTCATCTCAGGTTATGTGCTCAGGCAGAGAAAGATGACAACAAACAAAATATTGAGGAA
GGAATTGACAACAGAGATGGAGGTGCCCACTTCAGCTGATTCAATATGGGCAGTCTATGGTTCCCCTGATA
TTCCTAGACTCCTTAGAGATGTATTACTTCCTGGAGTTTTCGAAAGGCTGGATGTCATTGAAGGCAATGGA
GGTGTCGGCACTGTTCTTGATATTAGTTTTCCACCAGGGGCGGTACCACGTAGTTACAAGGAGAAGTTTGT
GAAGGTTGATCACAAGAACCGTCTGAAGGAAGTTGTGATGATCGAAGGAGGATATCTAGATCTAGGATGTA
CATTTTATATGGATAGAATCCATGTAATACCAAAAGGCCCTAATTCATGCGTAATCAAGTCGACCCTCATT
TATGAAATTCCTGGTGAGCTTGTGGACTCTGTTGGTTCTCTTATGTCTACTGAACCACTTGCAAGCATGGC
AGCAGTCATCTCGGATTACGTTCTCAAACAGAGAAAGATGACAGCAAACCAAATATTGAGGAAGGAATTGA
CAACAGAGATGGAGTTGGCTACTTCAGCTGACTCTATTTGGTCAGTTTATGGTTCCCCTGATATTCCTAGA
CTCCTCAGAGATGTATTACTTCCTGGAGTTTTCGAAGACTAG

>TFLNCS2
ATGAAGATGGAAGTTGTATTTGTTTTCTTTATGATCTTAGGAACAATTAATTGCCAGAAACTGATTCTGAC
AGGTAGGCCATTTCTTAACCGCCAAGGCATAATAAACCAAGTGTCTACAGTGACAAAAGGGGTTCATCATG
AGTTGGAAGTTGCTGCTTCGGCTGATGATATATGGAGTGTTTATAGCTGGCCTGGTTTGGCAAAGCATCTT
CCTGACTTGCTCCCTGGCGCTTTTGAAAAGCTCGAAATCATTGGTGATGGAGGTGTTGGTACCATCCTAGA
CATGACATTTACACCAGGTGAATTTCCTCATGAATACAAGGAGAAATTTATTTTAGTCGATAATGAGCATC
GTTTAAAGAAGGTGCAAATGATCGAGGGAGGTTATCTGGACTTAGGAGTAACATACTACATGGACACAATC
CAGGTTATTCCAACTGGTACAAATTCGTGTGTCATTAAATCCTCAACTGAGTACCATGTGAAACCTGAGTT
TGTCAAAATCGTTGAACCACTTATCACTACTGGTCCATTAGCTGCCATGGCGGAAGCCATCTCAAAACTTG
TTTTAGAACACAAATACAAAAGCAACTCAGATGAGATTGATGCCTCAAAAAACAATCTGAAGATGGTGATT
AATATGTAA

>CCHNCS1
ATGGAAGTGGCTACTTCAGCTGATTCTATATGGGCAGTCTACGGTTCCCCTGACATTCCTAGACTCCTCAG
AGATGTATTACTTCCTGGAGTTTTTGAGAAACTGGATGTCATTGAAGGCAGTGGAGGTGTCGGCACTGTTC
TTGATATTGCTTTTCCTCCAGGGGCGGTACCTCGTACTTACAAAGAGAAGTTCGTAAAGGTTGATCACAAG
AACCGTTTGAAGGAAGTCGTGATGATTGAAGGCGGATATCTGGACTTAGGATGTACATTTTATATGGATAG
AATCCATGTCTTACCAAAAGGTCCTAATACATGCGTAATCAAATCGACTCTTATCTATGAAGTTCCAGACG
AGTTCGCTGATGCAGTTGGTTCTCTTATCTCCGTTGAACCACTAGCAAGCATGGCAGAAGTAATATCAGGT
TATGTTCTCAAGCAGAAGAAGGAAGCAAAGAAAATATTAAGGAAGGAATTAACACACGAATTGGAGGTGCC
TACTTCAGCTGATTCAATATGGGCAGTCTATGGTTCCCCTGATATTCCAAGATTGCTCAGAGATGTATTAC
TTCCTGGTGTGTTTGAAAAGCTAGACATCGTGGAAGGCAATGGAGGTGTTGGTACTGTTCTTGACATTGCT
TTTCCTCCAGGGGCGGTACCTCGTAGTTACAAGGAGAAGTTTGTAAAGGTTGATCACGATAAGCATTTGAA
AGAAGTTGTGATGATCGAAGGAGGATATTTGGATCTAGGATGCACATTCTATATGGATAGAATCCATGTCC
TACCGAAAGGTCCTAATTCTTGTGTTATCGAATCGTCTCTTATTTATGAAGTCCGGGAAGAGCTCGCTGAT
GTCGTTGGTTCTCTTATCTCAATTGAACCACTTGCTAGCATGGCAGAAGTCATCTCAAGTTATGTTCTCAA
ACAACAACTCCGAGTGTTTGGAGTTGTAGTTCAACCAAGAGTAGGATTAAGTCTTTTGCTCTGCCTTATAC
TGTGTCTAGTCATATTAGGAGGTCTTTTGATCGGCGGTGTTTCGATATAA

>SDINCS1
ATGAGGAAGGAAGTACGATATGAGATGGAGGTACCTACCTCAGCTGATTCAATTTGGGCAGTTTACAGTTC
ACATGATATTCCAAGGCTCCTCAAAGAAGTTCTTCTCCCTGGTGTCTTTGAAAAGCTTGATGTCATTGAAG
GTGATGGTGGTGTTGGTACTGTTCTTGACATTGCTTTCCCACCAGGGGCGGTACCACGCACTTACAAGGAG
AAATTTGTGACAATCAATCATGAGAAGCGATTGAAAGAGGTGATTATGATTGAAGGAGGGTATTTGGATAT
GGGATGTACATTTTACATGGACAGAATCCATGTCCTAGAGAAAGGTCCCAAATCATGCATTATTGCATCGG
CTATTATCTATGAGGTGAAAGAAGAATTCGCCGATGTCGTCGTTCCTCTAATCACGACTGAACCATTGGCT
AGCATGGCAGAGGTCATCTCCAATTACGTTCTTAAGAAACAACGCCGTGTAAGGAAGGAATTAACATATGA
GATGGAGGTTCCTACCTCAGCTGATTCAATTTGGGCAGTTTACAGTTCACATGATATTCCAAGGCTCCTCA
AAGAAGTCCTTCTCCCTGGTGTGTTTGAAGAGCTTGATGTCATTGAGGGTGATGGTGGTGTTGGTACTGTT
CTTGACATTGCTTTCCCTCCAGGTGCGGTACCACGCACTTACAAGGAGAAATTCGTGAAGATCAATCACGA
GAAGCGATTGAAAGAGGTGGTGATGATTGAAGGAGGGTATTTGGATATGGGATGTACATTTTACATGGACA
GAATCCATGTCCTAGAGAAAGGTCCTAATTCTTGCGTTATTGAATCTGCTATTATCTACGAGGTGAAAGAA
GAATTTGCTGATGTCGTCGTTCCACTAATCACGACCGAACCACTAGCTAGCATGGCAGAGGTCATCTCAAA
TTACGTTCTAAAGAAACAAATCCATGTGTTTGGCTATGTAATTAAACCAAAACTTGGATTAAGTCTTTTGC
TCTGCTTCATTCTCTGCCTCGTTTTACTTGGAGTTTTATTGATTGGAGGTGTTCCACTCTAA

>PBRNCS5
ATGATGAGGAAAGTAATCAAATACGATATGGAGGTAGCTACCTCAGCTGATTCAGTATGGGCAGTTTACAG
TTCACCGGATATTCCAAGGCTTCTCAGGGATGTTCTACTTCCCGGCGTCTTCGAGAAATTAGACGTCATTG
AAGGGAATGGCGGCGTCGGTACAGTTCTTGACATTGCTTTTCCTCCAGGTGCGGTTCCTCGAAGTTACAAA
GAGAAATTCGTCAACATCGACCGTGTAAAGCGATTGAAAGAAGTGATCATGATTGAAGGAGGATACCTGGA
CATGGGATGCACATTTTACCTGGACAGGATCCATGTCGTGGAGAAAACTCCGAGCTCATGTGTTATTGAAT
CGTCTATTGTTTATGAAGTGGAAGAAGAGTACGCTGATGTCATGTCAAAATTGATCACCACTGAACCATTG
AAGTCGATGGCGGAAGTGATTTCTAATTACGTTATCCAGAAAGAATCAGTTTCCGCAAGAAACATTTTCAA
CAGGCAATCTGTAGTGAAGAAGGAGATTCATTACGACCTGGAGGTACCAACCTCAGCTGATTCGATCTGGG
CAGTTTACAGCAATCCCGATATCCCTCGACTACTTAGAGATGTTCTGCTTCCTGGCGTTTTCGAGAAATTG
GATGTCATTGAAGGGAATGGTGGTGTTGGGACTGTTCTTGATATCGTTTTTCCTCCAGGTGCGGTGCCTCG
TCGTTACAAGGAGAAATTTGTGAACATCAACCACGAGAAGCGATTAAAAGAAGTGATTATGATCGAAGGAG
GGTACTTAGACATGGGATGCACATTTTACCTGGACAGGATCCATGTTGTAGAAAAAACCTCTAAATCTTGC
ATCATTAAATCTTCTATTGTTTACGAAGTGAAGCAAGAGTGCGCTGAAGCAATATCTAAGTTGATCACGAC
GGAGCCGTTGAAGTCGATGGCACAAGTCATCGCTAATTATGTTCTTAAGAAACAATCTGTTTCTGACACAA
ACATTCCTAAGAAGCAATCTGTGTTGAGGAAAGAAATTACTTATGAAACGGAGGTGCAAACCTCAGTTGAT
TCTATTTGGAACGTCTACAGTTCTCCTGACATCCCTCGACTACTTAGAGATGTTCTGCTTCCTGGTGTTTT
TGAGAAGCTAGATGTCATTGCAGGCAATGGTGGCGTTGGGACTGTACTGGACATTGCTTTCCCTCTAGGTG
CAGTGCCGCGGAGGTACAAGGAGAAATTTGTGAAGATCAACCAGGAGAAGCGATTGAAAGAAGTGATTATG
ATCGAAGGAGGATACTTAGACATGGGGTGCACATTTTACATGGACAGGATCCATGTACTTGAGAAAACCCC
AAACTCATGTGTCATTGAATCCTCTATTATCTACGAAGTTAAAGAAGAGTTTGCTGATAAAATGGCTAAGC
TAATCACAACGGAACCATTGCAGTCGATGGCAGAAGTCATCACTGGTTATGTTCTTAAGAAACGACTCCAA
GTATTTGGATTCGAGATTAAGCCAAACTTAAGATTCAATCTTTTGCTATGTATGATTATCTGCTTGGTTAT
AGCTGGAGGTATGTTGATTGGACGTGTTCCACTCTAA

>PBRNCS4
ATGATCGAAGGAGGGTATCTGGACATGGGATGCACGTTTTATCTGGACAGGATCCATGTAGTGGAGAAAAC
TCCGAGCTCATGTGTTATTGAATCGTCTATTGTTTATGAAGTGAAGCAAGAGTGTGCTGAAGCAATATCTA
AGTTGATCACGACGGAGCCATTGAAGTCGATGGCAGAAGTCATCTCTAATTATGTTCTTAAGAAACAATCT
GTTTCTGACACAAACATTCCTAAGAAGCAATCTGTGTTGAGGAAAGAAATTACTTATGAAACGGAGGTGCA
AACGTCAGCTGATTCGATTTGGAACGTCTACAGTTCTCCAGACATCCCTCGACTACTTAGAGATGTTCTGC
TTCCTGGTGTTTTTGAGAAGCTAGATGTCATTGCAGGCAATGGCGGCGTTGGGACTGTACTGGATATCGCT
TTCCCTCTAGGTGCAGTGCCGCGGAGGTACAAGGAGAGATTTGTGAAAATCAATCACGAGAAGCGGTTGAA
AGAAGTGGTTATGATCGAAGGAGGGTACTTGGACATGGGCTGCACATTTTACATGGACAGGATTCATGTCT
TTGACAAAACCCCAAACTCATGTGTCATTGAATCCTCTATTATCTACGAAGTTAAAGAAGAGTATGCTGAT
AAAATGGCTAAGCTAATCACAACAGAACCATTGGAATCCATGGCAGAAGTCATCTCTGGTTATGTTCTTAA
GAAACGACTCCAAGTATTTGGATTCGAGATTCAGCCAACATTAAGATTCAATCTTTTGCTATGTTTGATTA
TCTGCTTGGTTATAGCTGGAGGTATGTTGATTGGACGTGTTCCACTCTAA

>CCHNCS5
ATGAGGAAGGAACTCACAAATGAGTTGGAGGTCGCAGCCCCTGCTGATGCTGTCTGGGCAGTTTACAGCTC
CCCGGATCTCCCAAAAATCATAGTTGAATTACTTCCTAGTGTCTTCGAAAAGATTGAAATCGTTGAAGGAG
ACGGAGGTGTTGGTACCGTTCTGTACGTTGTTTTTCCTCCAGGATCAGTTCCATTAACTTACAAGGAGAAG
TTCGTGACGATTGATCACGAGAAGCGTCTGAAGGAGGTGCTACAGATTGAAGGAGGATATTTGGACCTAGG
GTGTACGTTTTACATGGACAGCTTCCATATACTAGAGAAAGATTGTGATTCATGCATCATCAAATCCATAA
CAGCATACGAAGTCAGGGATGATGTTGTTGATAATGTTTCCTCTCTTATCTCCATTGATTCGCTCGCTAAC
ATGGCTGAAGCCATCTCAAAGTATGTCCTTGAGAAACAAGAAGCCGCAACTAAGCATGGACATGGAGATGA
TAGGGAAAGGACTGGTCTTTGTTGGCCTTTCAATTGTTTGGGTTAA

>XSINCS1
ATGAGGATGGAAGTTGTTCTAGTTGTTTTCTTACTGTTCATAGGTACTGTAAATTGTGAAAGAATGATATT
CAGTGGGCGTCCTCTCCTCCATCGCGTAACAAATGAGGAGACTGTAATCCTTTATCATGAGCTGGAAGTAC
CTGCTTCGGTGGATGAACTGTGGAGTGTCGAAGGTTCGCCTGAGTTGGGCAAGAATTTGCCTGACTTGCTC
CCTGGTATATTTGCAGACTTCAAAATTACTGGTGACGGAGGTGAAGGTTCCATCCTGGATATGACATTCCC
CCCAGGTCAGTTTCCACATCATTACAGGGAGAAGTTCGTGTTTTTCGATCACAAGAATCATTACAAGTTAG
TACAAATGATCGACGGTGATTTTTTCGATCTAGGTGTTACATACTATATGGATACAATCCGTGTTGTTGCA
ACAGGCCCTGATTCATGTGTCATCAAGTCTTCAACGGAATATCATGTAAAAGTTGAGTTTGCCAAGATCGT
CAAACCACTTATTGACACTGTACCACTAGCTATAATGTCTGAAGCGATTGCAAAGGTTGTTCTTGAGAAGA
AATACAAGAGATCAGAGTAA

>PSONCS3
ATGAGGAAAGTAATCAAATACGATATGGAGGTAGCTGTCTCAGCTGATTCAGTTTGGGCAGTTTACAGTTC
ACCGGATATTCCTAGACTTCTCAGAGACGTTCTACTTCCCGGTGTCTTCGAGAAGTTAGATGTTATTGAAG
GGAATGGCGGCGTCGGAACAGTTCTTGACATTGTTTTCCCTCCAGGTGCGGTTCCTCGAAGTTACAAGGAG
AAATTTGTCAATATCGATCGCGAAAAGCGATTGAAAGAAGTGATCATGATCGAAGGAGGATACCTGGACAT
GGGATGCACATTTTACTTGGATAGGATCCATGTAGTGGAGAAAACCAAGAGCTCATGCGTTATTGAATCGT
CTATTGTTTACGATGCGAAAGAAGAGTGCGCCGATGCCATGTCTAAATTGATCACAACTGAACCATTGAAG
TCCATGGCGGAAGTCATTTCTAATTACGTTATTCAGAAAGAATCATTTTCTGCCAGAAACATTCTAAGCAA
GCAATCTGTAGTGAAGAAGGAGATTCGATACGACCTGGAGGTACCAATCTCAGCTGATTCTATCTGGTCAG
TTTACAGCTGCCCTGATATCCCTCGGCTTCTTAGAGATGTTCTACTTCCTGGTGTGTTCGAGAAATTGGAT
GTCATTGAAGGGGATGGTGGTGTTGGGACTGTTCTTGACATTGTCTTCCCTCCAGGTGCAGTTCCTCGAAG
TTACAAAGAGAAATTCGTTAACATTGACCGCGAGAAGCGATTGAAAGAGGTTATCATGATTGAAGGAGGAT
ACTTGGACATGGGATGCACGTTTTACTTGGACAGGATCCATGTAGTGGAGAAAAGCCTGAGCTCGTGTGTT
ATCGAATCGTCTATTGTTTATGAAGTAAAAGAAGAGTATGTTGATGCCATGTCTAAATTAATCACAACTGA
ACCATTGAAGTCGATGGCGGAAGTCATCTCTAATTACGTTATCCAGAGAGAATCATTTTCCGCAAGAAACA
TTCTCAACAAAAATTCTTTGGTGAAGAAGGAGATTCGGTATGACCTGGAGGTTCCAACCTCAGCTGATTCT
ATCTGGTCAGTTTACAGCTGCCCGGATATTCCTCGGCTTCTTAGAGATGTTTTACTTCCCGGTGTGTTCCA
GAAATTGGATGTTATCGAAGGGAATGGTGGTGTTGGTACAGTTCTTGATATCGTTTTTCCTCCAGGTGCGG
TACCTCGTAGTTACAAGGAGAAATTTGTGAACATCAACCACGAAAAGCGATTAAAAGAAGTGATTATGATC
GAAGGAGGATATTTAGACATGGGATGCACATCTTACCTGGACAGGATCCATGTAGTTGAAAAAAACCTCTAA
ATCTTGTATCATTAAATCTTCTGTTGTCTATGAAGTGAAGCAAGAGTGTGTTGAGGCAATGTCTAAGTTGA
TCACGACGGAACCATTGAAGTCGATGGCAGAAGTTATCTCTAATTACGCTATGAAGCAACAATCTGTTTCT
GAGAGAAACATTCCTAAGAAGCAATCTCTACTGAGGAAGGAAATTACTTATGAAACGGAGGTGCAAACTTC
TGCTGATTCAATTTGGAACGTCTACAGTTCTCCTGACATCCCTCGACTACTTAGAGATGTTCTGCTTCCTG
GTGTTTTTGAAAAGCTAGATGTCATTGCAGGCAATGGTGGAGTTGGTACGGTACTGGATATTGCCTTCCCT
CTAGGTGCAGTGCGACGGAGGTACAAGGAGAAATTTGTGAAGATCAACCATGAGAAGCGATTGAAAGAAGT
GGTGATGATCGAAGGAGGATACTTAGACATGGGGTGCACATTTTACATGGACAGGATCCATGTCTTTGAGA
AAACCCCAAACTCATGTGTTATCGAATCCTCGATCATTACGAAGTTAAAGAAGAGTATGCTGGTAAAATGG
CTAAGCTAA

Amino acid sequences

```
>PBRSC1NG_011_C02
MMRKVIKYDMEVATSADSVWAVYSSPDIPRLLRDVLLPGVFEKLDVIEGNGGVGTVLDIAFPPGAVPRSYK
EKFVNIDRVKRLKEVIMIEGGYLDMGCTFYLDRIHVVEKTPNSCVIESSIIYEVKEEFADKMAKLITTEPL
QSMAEVISGYVLKKRLQVFGFEIKPNLRFNLLLCLIICLVIAGGMLIGRVP

>PBRSC1NG_025_C08
MMRKVIKYDMEVATSADSVWAVYSSPDIPRLLRDVLLPGVFEKLDVIEGNGGVGTVLDIAFPPGAVPRSYK
EKFVNIDRVKRLKEVIMIEGGYLDMGCTFYLDRIHVVEKAPSSCVIESSIVYEVEEEYADVMSKLITTEPL
KSMAEVISNYVIQKESVSARNIFNRQSVVKKEIHYDLEVPTSADSIWAVYSNPDIPRLLRDVLLPGVFEKL
DVIAGNGGVGTILDIAFPLGAVPRRYKEKFVKINHEKRLKEVIMIEGGYLDMGCTFYMDRIHVFEKTPNSC
VIESSIIYEVKEEYADKMAKLITTEPLQSMAEVISGYVLKKRLQVFGFEIKPTLRFNLLLCLIICLVIAGG
MLIGRVPL

>TFLNCS
MMKMEVVFVFLMLLGTINCQKLILTGRPFLHHQGIINQVSTVTKVIHHELEVAASADDIWTVYSWPGLAKH
LPDLLPGAFEKLEIIGDGGVGTILDMTFVPGEFPHEYKEKFILVDNEHRLKKVQMIEGGYLDLGVTYYMDT
IHVVPTGKDSCVIKSSTEYHVKPEFVKIVEPLITTGPLAAMADAISKLVLEHKSKSNSDEIEAAIITV

>AMENCS1
MSKLITTIPLKSMSEVIANYVLKNQSVIRKEVTYELEVPTSADSIWAVYSSPNIPTLLRDVLLPGVFEKLD
VIEGNGGVGTVLDIVFPPGAVPRCYKEKFINIDNKKRLKEVIMIEGGHLDMGCTYYLDRIHVIAKTPNSCV
IKSSIIYDVKKEYAEAMSKLITTIPLKSMSEVIANYVLKNQSVIRKEVTYELQVPTSADSIWAVYSSPNIP
TILRDVLLPGVFERLDVIKGNGGVGTVLDIVSHPGNSMYYYFFTIYPTFNFITILVTMVNST

>AMENCS2
MSKLITTAPLKSMSEAIANYVLKKQSVIRKVVTYELEVPASADSIWAVYSSPNIPTLLRDVLLPGVFEKLD
VIEGNGGVGTVLDIVFPPGAVPRRYKEKFVKINNEKRLKEVIMIEGGYLDMGCTSYMDRIHVLEKTPNSCV
IESSIIYEVKQEYADEMSKLITTVPLKSMSEVIANYVLKKQFRVFGYEIKPKLGLSLLLCLIICLVILGGL
LIAGVPV

>CSANCS
MGKRIQKKEAKKILRKELTHELEVPTSADSIWAVYGSPDIPRLLRDVLLPGVFEKLDIIEGNGGVGTVLDI
AFPPGTVPRSYKEKFVKVDHDKHLKEVVMIEGGYLDLGCTFYMDRIHVLPKGPNSCVIESSLIYEVREELA
DAVGSLISIEPLASMAEVVSSYVLKQQLRVFGVVVQPRVGLSLLLCLILCLVILGGLLIGGVSI

>PSONCS1
MSKLITTEPLKSMAEVISNYAMKQQSVSERNIPKKQSLLRKEITYETEVQTSADSIWNVYSSPDIPRLLRD
VLLPGVFEKLDVIAGNGGVGTVLDIAFPLGAVPRRYKEKFVKINHEKRLKEVVMIEGGYLDMGCTFYMDRI
HIFEKTPNSCVIESSIIYEVKEEYAGKMAKLITTEPLESMAEVISGYVLKKRLQVFGFEIKPKLRFNLLLC
LIICLVIAGGMFVAGVPL

>PSONCS2
MSKLITTEPLKSMAEVISNYVIQRESFSARNILNKNSLVKKEIRYDLEVPTSADSIWSVYSCPDIPRLLRD
VLLPGVFQKLDVIEGNGGVGTVLDIVFPPGAVPRSYKEKFVNINHEKRLKEVIMIEGGYLDMGCTFYMDRI
HIFEKTPNSCVIESSIIYEVKEEYAGKMAKLITTEPLESMAEVISGYVLKKRLQVFGFEIKPKLRFNLLLC
LIICLVIAGGMFVAGVPL

>CJAPR10A
MRMEVVLVVFLMFIGTINCERLIFNGRPLLHRVTKEETVMLYHELEVAASADEVWSVEGSPELGLHLPDLL
PAGIFAKFEITGDGGEGSILDMTFPPGQFPHHYREKFVFFDHKNRYKLVEQIDGDFFDLGVTYYMDTIRVV
ATGPDSCVIKSTTEYHVKPEFAKIVKPLIDTVPLAIMSEAIAKVVLENKHKSSE

>PBRNCS2 (PBRST1PF_rep_c6824)
MMRKVIKYDMEVATSADSVWAVYSSPDIPRLLRDVLLPGVFEKLDVIEGNGGVGTVLDIAFPPGAVPRSYK
EKFVNIDRVKRLKEVIMIEGGYLDMGCTFYLDRIHVVEKTPSSCVIESSIVYEVEEEYADAMSKLITTEPL
KSMAEVISNYVIQKESVSARNIFNRQSVVKKEIHYDLEVPTSADSIWAVYSNPDIPRLLRDVLLPGVFEKL
```

DVIEGNGGVGTVLDIVFPPGAVPRCYKEKFVTMDHQKRLKEVIMIEGGYLDMGCTSYLDRIHVIEKTSKSC
IIKSSVVYEVKQECAEAISKLITTEPLKSMAEVISNYVLKKQSVSDTNNIAKKQSVLRKEITYETEVQTSA
DSIWNVYSSPDIPPTT

>PBRNCS3 (PBRST1PF_rep_c8842)
MDIIEGDGGVGTVLDVVFQPGAVPQSYKERFETVDHEKRILEVRIIQGGYLEMGCTSYLNRMHVIEITSKS
CVIKSSVIYDVKEECADAMSKLITTIQLESMAKVVADYVLKKQSASDTSIPKKQSLMRKEITHEMEVQTSA
DSIWDIYSSPDIPRLLRDVLLPGAFEKLHVIQGNGGVGTVLDIALPLGAVPRNYKEKFVKINHEKRLKEAV
MIEGGYADMGCTFYMHRIHVLEKTPKSCVIESSIVYEVKEEYADKMSKLITTEPLQSMAEAISSYVLKKQF
QVFGLEVKPKLVLSLFLCLIIFLAIVGGFLIGGLKA

>PBRNCS4 (PBRContig25754)
MIEGGYLDMGCTFYLDRIHVVEKTPSSCVIESSIVYEVKQECAEAISKLITTEPLKSMAEVIANYVLKKQS
VSDTNIPKKQSVLRKEITYETEVQTSVDSIWNVYSSPDIPRLLRDVLLPGVFEKLDVIAGNGGVGTVLDIA
FPLGAVPRRYKERFVKINHEKRLKEVVMIEGGYLDMGCTFYMDRIHVFDKTPNSCVIESSIIYEVKEEYAD
KMAKLITTEPLQSMAEVISGYVLKKRLQVFGFEIKPNLRFNLLLCLIICLVIAGGMLIGRVPL

>PBRNCS5 (PBRContig45733)
MMRKVIKYDMEVATSADSVWAVYSSPDIPRLLRDVLLPGVFEKLDVIEGNGGVGTVLDIAFPPGAVPRSYK
EKFVNIDRVKRLKEVIMIEGGYLDMGCTFYLDRIHVVEKTPSSCVIESSIVYEVEEEYADAMSKLITTEPL
KSMAEVISNYVIQKESVSARNIFNRQSVVKKEIRYDLEVPTSADSIWAVYSNPDIPRLLRDVLLPGVFEKL
DVIEGNGGVGTVLDIVFPPGAVPRRYKEKFVNINHEKRLKEVIMIEGGYLDMGCTFYLDRIHVVEKTSKSC
IIKSSIVYEVKQECAEAISKLITTEPLKSMAQVIANYVLKKQSVSDTNIPKKQSVLRKEITYETEVQTSVD
SIWNVYSSPDIPRLLRDVLLPGVFEKLDVIAGNGGVGTVLDIAFPLGAVPRRYKEKFVKINHEKRLKEVIM
IEGGYLDMGCTFYMDRIHVLEKTPNSCVIESSIIYEVKEEFADKMAKLITTEPLQSMAEVISAYVLRKRFE
VFGLEIKQKLRYNLLLCLIICLVIAGGMLIGRVPL

>SCANCS1 (SCAContig30427)
MRKELTHEMEVPASADAIWAVYSSHDIPRLLKEVLLPGVFEKLDVIAGDGGVGTVLDIAFPPGAVPRRYKE
KFVKINHEKRLKEVEMIEGGYLDMGCTFYMDRIHVVEKGPNSCVIESAIIYVVKDECADVVVPLITTEPLA
SMAEVISNYVLRKQIRLFGYVIKPKLGLSILLSLILCLVILGVLLIGGVPF

>CMANCS1 (CMAST2PF_rep_c1557)
MIEGGYLDMGCTFYMDRIHVVKKGPNSCVIASAIIYEVKEEFVDVVVPLITTEPLASMAEVISNYVLKKQR
RVRKELTYEMEVPTSADSIWAVYSSHDIPRLLKEVLLPGVFEKLDVIEGDGGVGTVLDIAFPPGAVPRTYK
EKFVKINHEKRLKEVVMIEGGYLDMGCTFYMDRIHVLEKSPNSCVIESSIIYEVKEEFADVVGPLITTEPL
ASMSEVISNYVLKKQIRMFGYVIKPKLGLSLLLCFILCLVLLGVLLIGGVPL

>CMANCS2 (CMAContig5713)
MRKVIKYDMEVAVSADSVWAVYSSPDIPRLLRDVLLPGVFEKLDVIEGNGGVGTVLDIVFPPGAVPRSYKE
KFVNIDREKRLKEVIMIEGGYLDMGCTFYLDRIHVVEKTKSSCVIESSIVYDVKEECADAMSKLITTEPLK
SMAEVISNYVIQKESFSARNILSKQSVVKKEIRYDLEVPISADSIWSVYSCPDIPRLLRDVLLPGVFQKLD
VIEGNGGVGTVLDIVFPPGAVPRSYKEKFVNINHEKRLKEVIMIEGGYLDMGCTSYLDRIHVVEKTSKSCI
IKSSVVYEVKQECVEAMSKLITTEPLKSMAEVISNYAMKQQSVSERNIPKKQSLLRKEITYETEVQTSADS
IWNVYSSPDIPRLLRDVLLPGVFEKLDVIAGNGGVGTVLDIAFPLGAVPRRYKEKFVKINHEKRLKEVVMI
EGGYLDMGCTFYMDRIHVFEKTPNSCVIESSIIYEVKEEYAAKMAKLITTEPLESMAEVISGYVLKKRLQV
FGFEIKPKLRFNLLLCLIICLVIAGGMFVAGVPL

>SDINCS1 (SDIST1PF_rep_c489)
MRKEVRYEMEVPTSADSIWAVYSSHDIPRLLKEVLLPGVFEKLDVIEGDGGVGTVLDIAFPPGAVPRTYKE
KFVTINHEKRLKEVIMIEGGYLDMGCTFYMDRIHVLEKGPKSCIIASAIIYEVKEEFADVVVPLITTEPLA
SMAEVISNYVLKKQRRVRKELTYEMEVPTSADSIWAVYSSHDIPRLLKEVLLPGVFEKLDVIEGDGGVGTV
LDIAFPPGAVPRTYKEKFVKINHEKRLKEVVMIEGGYLDMGCTFYMDRIHVLEKGPNSCVIESAIIYEVKE
EFADVVVPLITTEPLASMAEVISNYVLKKQIHVFGYVIKPKLGLSLLLCFILCLVLLGVLLIGGVPL

>ECANCS1 (ECARTPF1_rep_c12486)
MIGGFLDMGCTFYMDRIHVVAKGPNSCIIKSTLIYEVKEEYADAMASLITVEPLASMAEVVANYVLHQQVR
VLGSVKRKELTHELEVAAPADAIWGVYSSPDIPRLLRDVLLPGVFEKLEVIQGNGGVGTVLEIVFHPGAIP

RRYKEKFVTINHKKRLKEVVMIGGYLDMGCTLYMDRIHVVSKGPNSCVIKSTLIYEVKAESADAMASTITI
DPLASMAQVISNYVLKNQMQVLGSVKRRELTHELEVAASADAIWGVYGSKRYSKASQGCFASWCFRKVRSH

>ECANCS2 (ECAContig18893)
MIGGYLDMGCTLYMDRIHVVEKGPNSCVIKSTLIYEVKAEYADAMASLITVDPLASMAQVISNYVLKNQGQ
VLGSIKRRELKHELEVAVSADAIWGVIGSKDIPRLLRDVLLPGVFEKLDVIEGDGGVGTVLEIVFPPGAVP
RKYREKFVKVDHEKRLKEVIMIGGYLDMGCTFYMDRIHVVAKGPNSCIIKSTLIYEVKEEYADAMASLITV
EPLASMAEVVANYVLHQQVRVLGSVKRKELTHELEVAASADAIWGVYSSPDIPRLLRDVLLPGVFEKLEVI
QGNGGVGTVLEIVFHPGAIPRRYKEKFVTINHKKRLKEVVMIGGYLDMGCTLYMDRIHVVSKGPNSCVIKS
TLIYEVKAESADAMASTITIDPLASMAQVISNYVLKNQMQVLGSVKRRELTHELEVAASADAIWGVYGSKD
IPRLLRDVLLPGVFEKLEVIEGDGGVGTVLQIVFPPGAIPRRYKEKFVKVDQKLRLKEVIMIGGYLDMGCT
FYMDRIHVVPKGLNSCVIKSTLIYEVKDEYADAMSSLITVEPLASMAEVVSNYVLNKKKLMITRKELTHEL
EVAASADAIWSVYSSPDIPRLLRDVLLPGVFEKLEVVQGNGGVGTVLEIVFPKGSVPRRYKEKFVKINDEK
KLKEVIMIEGGYLDLGCTFYMDKIHVLPKGPNSCVIESSLIYEVKEENAKAMASLITVEPLASMAEVVANY
VLKKQIRVLGYVVKPRVGYSVLVGLLLCLVLLGVLLLSGVNI

>AMENCS1 (AMEST1PF_rep_c2186)
MRKEVVYELEVPTSADSIWAVYSSPNIPTLLRDVLLPGVFEKLDVIEGNGGVGTVLNIVFPPGAVPRCYKE
KFINIDNKKRLKEVIMIEGGYLDMGCTFYMDRIHVIAETPNSCVIKSSIIYDVKKEYAEAMSKLITTIPLK
SMSEVIANYVLKNQSVIRKEVTYELQVPTSD

>AMENCS2 (AMEcomp935_c0_seq1)
MKFELVNELEVPASANDVWAIYSSPDFPKLLTKLVPGILESVEYVEGDGHLGTVIHLVYVPGSVPLSYKEK
FVTIDHEKRLKEAVHVEGGFLEMGVTFYMNSFEIIEKGSDCCIIRSMTKCEIEDKEIMNLISHISVANVTV
LAMTISKYVQQHKK

>TFLNCS2 (TFLRTPF1_rep_c456)
MKMEVVFVFFMILGTINCQKLILTGRPFLNRQGIINQVSTVTKGVHHELEVAASADDIWSVYSWPGLAKHL
PDLLPGAFEKLEIIGDGGVGTILDMTFTPGEFPHEYKEKFILVDNEHRLKKVQMIEGGYLDLGVTYYMDTI
QVIPTGTNSCVIKSSTEYHVKPEFVKIVEPLITTGPLAAMAEAISKLVLEHKYKSNSDEIDASKNNLKMVI
NM

>TFLNCS3 (TFLRTPF1_rep_c2110)
MKMEATVFVFLMFLGTINCQKLIMAGRPFLHHQGIINQEFTVTKVLHHELEVAASADDIWGVYSSPHLVFH
LTDLLPGAFEKVQVIGDGGVGTILDMTFAPGEFPHEYKEKFIVVDNEHRLKKVQMIEGGYLDLGVTYYMDT
IQVVPTGTNSCVIKSSTEYHVKPELLKIVEPLITTGPVAAMAEAISKLVLEYKYKSHSDEIHAGLNNNLKM
VINNI

>TFLNCS4 (TFLcomp2119_c0_seq1)
MRKELTHEMEVPASADAIWAVYGSPDIPRLLKEVLLPGVFEKLDVIEGDGGVGTVLDIAFPPGAVPRAYKE
KFMKVNHEKRLKEVEMIEGGYLDMGCTFYMDRIHVVEKGPNACVIESAIIYEVKDEFADVVVPLITTEPLA
SMAEVISNYVLKNQFRVFGYVIKPKLGLSLLLCFILCLVLLGGLLIGGVPL

>TFLNCS5 (TFLcomp21856_c0_seq1)
MRKELTNEMEVAASADEIWAVYSSPNLPKLIVQLLPAVFERIYILEGDGGVGTVLYILSPPGSVPRSYKEK
FITIDHEKRLKEVQEIEGGYLDMGVTFYMDTFYILEKGPDSCIIKSMTTYEIKDELADKVASLISIDSLVG
MAKAITKYVLDQKKAAVDSSA

>BTHNCS1 (BTHRT1PF_c15840)
MVVAASADDVWAIYSSHDLPKLIVKLLPSVFKSIEIVEGDGGLGTVLDVKYPPGSIPLHYREKFITIDNEK
RLKEVRQIEDGLLALGCTFYMDSFHILEKDCHHEFFHIHEKNCHKKCIIKSTTVYEVPDELAYKIEPLVTI
DSLVGMAHAISKYVLDKSC

>CCHNCS1 (CCHRT1PF_rep_c1173)
MYYFLEFFEKLDVIEGNGGVGTVLDIAFPPGAVPRSYKEKFVKVDHKNRLKEVVMIEGGYLDLGCTFYMDR
IHVLPKGANSCVIKSTLIYEIPDELVDSVGSLMSTEPLASMAKVISDYVLKQRKMTANKILRKELKTEMEV
ATSADSIWAVYGSPDIPRLLRDVLLPGVFEKLDVIEGNGGVGTVLDIAFPPGAVPRTYKEKFVKVDHKNRL
KEVVMIEGGYLDLGCTFYMDRIHVLPKGPNTCVIKSTLIYEVPDEFADAVGSLISVEPLASMAEVISGYVL

KQKKEAKKILRKELTHELEVPTSADSIWAVYGSPDIPRLLRDVLLPGVFEKLDIVEGNGGVGTVLDIAFPP
GAVPRSYKEKFVKVDHDKHLKEVVMIEGGYLDLGCTFYMDRIHVLPKGPNSCVIESSLIYEVREELADVVG
SLISIEPLASMAEVISSYVLKQQLRVFGVVVQPRVGLSLLLCLILCLVILGGLLIGGVSI

>CCHNCS2 (CCHRT1PF_rep_c7133)
MRKELRHELEVATSADSIWAVYGSPDIPRLLRDVLLPGVFEKLDVIQGNGGVGTVLDIAFPPGAVPRTYKE
KFVKVDHKNRLKEVVMIEGGYLDLGCTFYMDRIHVLPSGPNTCIIKSTLIYEVPDELAYSVASLISVEPLA
SMAEVISGYVLRQRKMTTNKILRKELTTEMEVPTSADSIWAVYGSPDIPRLLRDVLLPGVFERLDVIEGNG
GVGTVLDISFPPGAVPRSYKEKFVKVDHKNRLKEVVMIEGGYLDLGCTFYMDRIHVIPKGPNSCVIKSTLI
YEIPGELVDSVGSLMSTEPLASMAAVISDYVLKQRKMTANQILRKELTTEMELATSADSIWSVYGSPDIPR
LLRDVLLPGVFERLGCH

>CCHNCS3 (CCHRT1PF_rep_c1524)
MMRKELVHEKEVCASADAVWGVYSSPNIPTLLRDKLLPGMFKRLEILEGDGGVGTILLLEFNNPAIIPHTY
LEKFMKLDHEKRLLEVEVVKGGYLDLGCTFYMSRIHILEKGPNSCVIESTLIFEAPEELMEYVSQYANLES
LISMAEVISKYVLEQQFRVFGVVVKKLKLGLSTIVLLCIFIFLVIVLGGLWIEGVSI

>CCHNCS4 (CCHRT1PF_rep_c156)
MRKHLVNELEVVVVPADTLWAIYSTTQFPKLIVQLLPIVVQNIEIDGDGSLGTVLNVIFVPGSVPLSYKEKI
VTIDHEKRLKEVVQIEGGYLDLGCSFYMSSFQILEKGRDSCIIKSMVTYELAKDADPSVADLVTIAAHAAI
AQVISKYVLDKQVAAAP

>CCHNCS5 (CCHRT1PF_rep_c2691)
MRKELTNELEVAAPADAVWAVYSSPDLPKIIVELLPSVFEKIEIVEGDGGVGTVLYVVFPPGSVPLTYKEK
FVTIDHEKRLKEVLQIEGGYLDLGCTFYMDSFHILEKDCDSCIIKSITAYEVRDDVVDNVSSLISIDSLAN
MAEAISKYVLEKQEAATKHGHGDDRERTGLCWPFNCLG

>NDONCS1 (NDORT3PF_rep_c12880)
MRKGIVFLFLVFLGCEVSQGRQLLESRLFRKSTIQKVLHHELPVAASAQEVWDVYSSPELPKHLPEILPGA
FEKVVVTGDGGVGTVLEMVFPPGEVPRSYKEKFVLIDDEQLLKKVEMIEGGYLDMGCTFYMDTIQIVPTGP
DSCIIKSSTEYYVKPEFADKVVPLISTIPLQAMAEAISNIVLANKAKNKSIIIEI

>NDONCS2 (NDORT3PF_rep_c17645)
MVFPPGEVPRSYKEKFVLIDDEQLLKKVEMIEGGYLNDLDCVHIKRTSHVQISTFNHFDMGCTFYMDTIQI
VPTGPDSCIIKSSTEYYVKPEFADKVVPLISTIPLQAMAEAISNIVLDKTKDQRNKEVINTNTKNNKIHHR
YVATIVIIR

>NDONCS3 (NDORT3PF_rep_c11505)
MRSGIVFLVLFFLGCEISQGRQLLESRLFRKSTIRKVLHHELPVAASAQEVWDVYSSPELPKHLPEILPGA
FKKVVVTGDGGVGTVIEMVFPPGVVPHRYKEKFVLIDDEKFLKKVEMIEGGYLDMGCTFYMDTIQIVPTGP
DSCIIKSSTEYYVKPEFADKVVPLISTVPLQAMAEAIAKIVLEFKAKHKGFIEI

>NDONCS4 (NDORT3PF_rep_c14985)
MEVAASAGDIWAVYSSPDLPRLIVQLLPTVFEKIDIVEGDGGVGTVLHITFPPGSVPLTYKEKFVTIDNAN
RLKEVLQIEGGYLELGCTFYMDSFQIFEKGIDSCIIKSMTTYEVPDELADKVAPLISIDSLVPMAEAISKY
VIEKRH

>CTRNCS1 (CTRCC1PF_c5264)
MIKKELKHEMRVAASADDIWAVYSSPDLPNLILRLLPSVFDNIEIVEGNGGVGTVLHLTFPPGSVPLSYKE
KFVTINGNKRLKEVKQIQGGYLDMGCTFYMDSFHIEEKGCDSCVIVSKTEYEVPNEEIANQVELYISIDSL
ASMAQGHLGLCS

>HCANCS1 (HCARH2PF_rep_c19)
MKMAILFVFLMFLGKMNSEGLHLSGRPLLRAIISDKPNVIKVLKHELAVPASADKVWAVYSAPTLAFHLSD
LLPGAFEKVEVFGDGGVGTIIDMTFAPGEFPHEYKEKFILIDGKQRLKKVQMIEGGYLDLGVTYYMDTIHV
VPTGSNSCIIKSSTEYHVKPEAAKLVEPLITTEPLAAMAEVITKIVLENKSKSSEENQSSEAI

>NSANCS1 (NSART2PF_rep_c28)

MVQFSRESKQISIISDEEEGGEEETKEKKMMKVQVALAFLLILGAASCQELILQGRPLLGGARAWGTKSIK
KELKHEFKVAASADEVWSVYSAPELCKHLTDLLPGAFEDVEIIGDGGVGTILHMIFPPGEFPHEYKEKFVV
IDDKQRLKKVEMIEGGYLDIGVTYYMDTIHVVPTGSDSCVIKSSTEYHVKPEFEKIVEPLITTVPLAAMAE
AIAKIVLDNKTHSITI

>NSANCS2 (NSART2PF_rep_c877)
MVKIQLVLACLLLVVGAVNCQKLILQGRPLLGAWACGTIKKVLKHEFKVAASADEVWSVYSSPELCKHLTD
LLPGAFQDLEIIGDGGVGTILHMTFPPGEFPHEYKEKFVLIDDKRKLKKVEMIKGGYLDIGVTYYMDTIHV
VPTGSDSCVIKSSTEYHVRPECEKIVEPLITTEPLAAMAEAVSKIVLDAKIHSIITI

>MCANCS1 (MCAcomp5594_c0_seq1)
MIKKELKHELEVATSADEIWEVYSSPDLPILIVKLLPSVFEKIEILEGDGGVGTALRLTFPIGSVPLTYKE
KFVTINDWKRLKEVKQIEGGYLDMGCTFYMDSFHILRKGPKSCVIVSKTEYEVPNKEIASKVEPYISIDSL
RKMATAISDYVLNRATRKEVKHELEVAASADDVWEGYRSPDVGSLICPHVFEKIELVEGDGGVGTILQITH
PPGYVPHTYKEKYVTLDDHKRLLEVEQIEGGYLEMGCTFYMDSIHVVKKGDNSCVIVSKAKYEVPKELASQ
VEPYIAADAVANMARIISNNVLEKKKS

>XSINCS1 (XSIcomp133_c0_seq1)
MRMEVVLVVFLLFIGTVNCERMIFSGRPLLHRVTNEETVILYHELEVPASVDELWSVEGSPELGKNLPDLL
PGIFADFKITGDGGEGSILDMTFPPGQFPHHYREKFVFFDHKNHYKLVQMIDGDFFDLGVTYYMDTIRVVA
TGPDSCVIKSSTEYHVKVEFAKIVKPLIDTVPLAIMSEAIAKVVLEKKYKRSE

>PSONCS3 (PSO_STEM_rep_c3975)
MRKVIKYDMEVAVSADSVWAVYSSPDIPRLLRDVLLPGVFEKLDVIEGNGGVGTVLDIVFPPGAVPRSYKE
KFVNIDREKRLKEVIMIEGGYLDMGCTFYLDRIHVVEKTKSSCVIESSIVYDVKEECADAMSKLITTEPLK
SMAEVISNYVIQKESFSARNILSKQSVVKKEIRYDLEVPISADSIWSVYSCPDIPRLLRDVLLPGVFEKLD
VIEGDGGVGTVLDIVFPPGAVPRSYKEKFVNIDREKRLKEVIMIEGGYLDMGCTFYLDRIHVVEKSLSSCV
IESSIVYEVKEEYADAMSKLITTEPLKSMAEVISNYVIQRESFSARNILNKNSLVKKEIRYDLEVPTSADS
IWSVYSCPDIPRLLRDVLLPGVFQKLDVIEGNGGVGTVLDIVFPPGAVPRSYKEKFVNINHEKRLKEVIMI
EGGYLDMGCTSYLDRIHVVEKTSKSCIIKSSVVYEVKQECVEAMSKLITTEPLKSMAEVISNYAMKQQSVS
ERNIPKKQSLLRKEITYETEVQTSADSIWNVYSSPDIPRLLRDVLLPGVFEKLDVIAGNGGVGTVLDIAFP
LGAVPRRYKEKFVKINHEKRLKEVVMIEGGYLDMGCTFYMDRIHVFEKTPNSCVIESSIIYEVKEEYAGKM
AKLITTEPLESMAEVISGYVLKKRLQVFGFEIKPKLRFNLLLCLIICLVIAGGMFVAGVPL
